# Valorization of *Paris polyphylla* Byproducts: Integrated Multi-Omics and Molecular Docking Reveal the Anti-Melanogenic Mechanism of Plant-Derived Nanovesicles via the AKT/GSK3β/MITF Axis

**DOI:** 10.3390/cells15151420

**Published:** 2026-08-05

**Authors:** Peishi Feng, Li Tao, Xiaoli Chen, Han Yang, Yida Zhang, Ping Wang

**Affiliations:** 1 School of Pharmacy, Zhejiang University of Technology, Hangzhou 310014, China; fengpeishi@zjut.edu.cn (P.F.); 111123070006@zjut.edu.cn (L.T.); cxl857022587@126.com (X.C.); yanghan151@126.com (H.Y.); zyd18267328239@163.com (Y.Z.); 2 Zhejiang Provincial Key Laboratory of TCM for Innovative R & D and Digital Intelligent Manufacturing of TCM Great Health Products, Hangzhou 310014, China; 3 Zhejiang International Scientific & Technological Cooperation Base of Development and Utilization of Natural Products, Zhejiang University of Technology, Hangzhou 310014, China

**Keywords:** *Paris polyphylla* byproducts, Plant-Derived Nanovesicles (PDNVs), melanogenesis, multi-omics integration, molecular docking

## Abstract

**Highlights:**

**What are the main findings?**

- Plant-derived nanovesicles from *Paris polyphylla* stems/leaves (SL-EXO), unlike root-derived ones, safely and profoundly suppressed melanogenesis and effectively ameliorated the pigmentation-driving oxidative and senescent microenvironments in both B16F10 cells and in vivo zebrafish models.
- Integrated multi-omics and molecular docking revealed that SL-EXO utilizes chloroplast-derived biomimetic lipids to deliver specific flavonoid payloads, which physically inhibit AKT1 and arrest melanin synthesis by silencing the intracellular p-AKT/p-GSK3β/MITF signaling cascade.

**What are the implications of the main findings?**

- This study successfully decodes the “structure-efficacy” black box of plant-derived nanovesicles, demonstrating that their synergistic multi-target mechanism and liposomal architecture offer a vastly superior safety margin compared to traditional single-molecule depigmenting agent.
- It establishes a rigorous, multi-omics-guided molecular paradigm for the sustainable upcycling of discarded medicinal plant agricultural wastes into high-value, exceptionally safe natural nanotherapeutics for dermatological applications.

**Abstract:**

Valorizing agricultural byproducts into functional ingredients is highly desirable. Herein, plant-derived nanovesicles (PDNVs) from *Paris polyphylla* stems/leaves (SL-EXO) exhibited potent anti-melanogenic properties, whereas root-derived PDNVs were ineffective. In vitro, SL-EXO achieved 92.66% cell-free tyrosinase inhibition, while α-arbutin was 49.78%. In vivo, SL-EXO ameliorated the pigmentation-driving oxidative/senescent microenvironment in zebrafish and reduced macroscopic melanin by ~66%. Crucially, SL-EXO reversed α-MSH-induced hyperpigmentation in B16F10 cells while maintaining excellent biocompatibility up to 0.15 mg/mL, displaying a vastly superior safety margin compared to α-arbutin (which induced cytotoxicity at 0.075 mg/mL). To decode this, multi-omics profiling revealed that SL-EXO utilizes a chloroplast-derived biomimetic lipid architecture (enriched in MGDG/DGDG) to efficiently deliver potent flavonoid payloads. Molecular docking demonstrated exceptional predictive structural affinities (binding energies up to −10.9 kcal/mol) between these phytochemicals and AKT1. Finally, pharmacological rescue assays validated that SL-EXO arrests melanogenesis by targeting the AKT1 pathway, thereby downregulating the p-AKT/p-GSK3/MITF signaling cascade and silencing melanogenic genes. This study establishes a rigorous multi-omics paradigm for upcycling botanical wastes into exceptionally safe and efficacious natural anti-melanogenic nanotherapeutics.

## 1. Introduction

The phenomenon of skin hyperpigmentation results from excessive melanin production and/or abnormal distribution, leading to darkened skin. It not only increases aesthetic distress but also closely relates to skin aging and skin disorders [1,2]. The complex regulatory networks govern the physiological process of melanogenesis. Under environmental stimuli, such as ultraviolet (UV) irradiation, excessive intracellular accumulation of reactive oxygen species (ROS) leads to chronic oxidative stress and cellular senescence [3,4]. The microenvironmental conditions in pathological tissues provide the catalytic requirements for tyrosinase, a rate-limiting enzyme in melanin synthesis, and trigger continuous activation of upstream signaling pathways that upregulate MITF, the master transcriptional regulator of melanogenesis [5]. While synthetic and single-molecule depigmenting agents (e.g., hydroquinone and high-dose α-arbutin) demonstrate considerable in vitro enzyme-inhibitory efficacy, their practical clinical and cosmetic applications are frequently bottlenecked by severe limitations, including poor transdermal bioavailability, extreme chemical instability, and notorious dose-dependent cytotoxicity [6,7]. Consequently, the discovery of highly efficacious, safe, and naturally derived active ingredients equipped with efficient biological delivery systems remains an urgent imperative in dermatological sciences.

Recently, plant-derived nanovesicles (PDNVs) have emerged as a revolutionary frontier in natural product research. As nano-sized lipid bilayers naturally secreted by plant cells, PDNVs intrinsically circumvent the limitations of traditional plant extracts [8,9]. Their biomimetic liposomal architecture endows them with extraordinary biocompatibility and exceptional tissue-penetrating capabilities, allowing them to function as “smart nanocarriers” that safeguard and deliver diverse bioactive payloads (e.g., secondary metabolites, lipids, and functional proteins) directly into mammalian target cells [10]. Despite their immense potential, current research on PDNVs faces two critical gaps: First, the vast majority of studies focus on edible fruits or the primary medicinal parts of plants, leaving the immense potential of agricultural byproducts and medicinal plant wastes largely untapped [11,12]. Second, the tripartite relationship among tissue origin, nanovesicle composition, and pharmacological mechanism remains largely opaque, with comprehensive multi-omics decoding still lacking.

*Paris polyphylla*, a highly valued traditional medicinal plant, is globally cultivated for its rhizomes, which are rich in steroidal saponins. However, during agricultural harvesting, its massive non-medicinal parts, specifically the aerial stems/leaves and the subterranean fibrous roots, are routinely discarded as agricultural waste, causing severe resource squandering and environmental burden [13,14]. Given the distinct physiological environments of these tissues (e.g., UV-exposed photosynthetic aerial parts versus light-deprived roots), we hypothesize that the PDNVs derived from these byproducts possess vastly divergent phytochemical payloads and, consequently, distinct bioactivities.

To test this hypothesis and achieve the sustainable valorization of *P. polyphylla* waste, this study systematically isolated PDNVs from the discarded stems/leaves (SL-EXO) and fibrous roots (RP-EXO). Through an initial multidimensional screening (antioxidant, anti-tyrosinase, and in vivo zebrafish anti-inflammatory assays), a striking tissue-specific functional dichotomy was identified, prompting a deep-dive investigation into the highly potent SL-EXO. By integrating in vivo zebrafish models and in vitro B16F10 melanoma cell assays, we comprehensively evaluated the safety, microenvironment-modulating, and anti-melanogenic capabilities of SL-EXO. Ultimately, an integrated strategy combined metabolomics, lipidomics, proteomics, network pharmacology, molecular docking, and pharmacological rescue validation was orchestrated to decode the structural basis of SL-EXO and map its specific multi-target mechanism via the PI3K/AKT/GSK3β/MITF signaling axis. This work aims to establish a robust molecular paradigm for upcycling medicinal plant wastes into safe, high-value nanotherapeutics.

## 2. Materials and Methods

### 2.1. Materials and Reagents

The discarded fibrous roots and stems/leaves of *Paris polyphylla* were purchased from local farmers in Midu County, Dali City, Yunnan Province, China. Polyethylene glycol 6000 (PEG-6000) was obtained from Sinopharm Chemical Reagent Co., Ltd. (Shanghai, China). ABTS, tyrosinase, 2,7-dichlorodihydrofluorescein diacetate (DCFH-DA), hydrogen peroxide (H_2_O_2_), doxorubicin hydrochloride, and tricaine methanesulfonate (MS-222) were purchased from Shanghai Aladdin Biochemical Technology Co., Ltd. (Shanghai, China). 1-phenyl-2-thiourea (PTU) was obtained from Sigma-Aldrich (St. Louis, MO, USA). RIPA Lysis Buffer was provided by Yeasen Biotechnology Co., Ltd. (Shanghai, China).

### 2.2. Isolation and Purification of SL-EXO and RP-EXO

SL-EXO and RP-EXO were isolated and purified according to our previously established method [15]. Fresh plant tissues were briefly homogenized and then differentially centrifuged to remove debris. The enriched nanovesicles were obtained via precipitation with 10% PEG-6000. The enriched nanovesicles were then purified via electrodialysis (ED) and concentrated via ultrafiltration. Final protein concentrations were determined using a BCA assay. Detailed procedures are provided in the supplementary information Method S1.

Specifically, to overcome the non-specific co-precipitation inherent to crude PEG, an external electric field was applied to actively drive charged extravesicular free proteins and small molecules through the semi-permeable membrane, while the intact PDNVs were sterically retained. To prevent the membrane pores from being blocked by the vesicles, the fresh electrophoretic buffer was replaced, and the electrophoretic polarity was reversed every 30 min for a total of 5 cycles (2.5 h). Importantly, to counteract the Joule heating generated by the electric current and to prevent thermal degradation of the vesicles, the entire electrodialysis apparatus was strictly submerged in ice packs to maintain a consistently low temperature throughout the purification process.

### 2.3. Characterization of SL-EXO and RP-EXO

The size distributions, concentrations, and zeta potentials of SL-EXO and RP-EXO were determined using a NanoSight NS300 (Malvern, UK) and a Nano-ZS90 (Malvern, UK), respectively. PDNVs morphology was characterized using transmission electron microscopy (TEM, HT7800, Hitachi, Japan). For TEM preparation, a 10 μL PDNVs suspension was adsorbed onto a copper grid for 15 min, incubated at room temperature for 20 min, and fixed with 2% paraformaldehyde (10 μL). The grid was then negatively stained with 2% phosphotungstic acid (10 μL) for 90 s. Excess stain was blotted with filter paper, and the grid was air-dried in the dark for 30 min before imaging.

### 2.4. In Vitro Antioxidant Activity Assay (ABTS Assay)

The ABTS radical scavenging activity of the nanovesicles was evaluated following a standard protocol with minor modifications [16]. Briefly, the ABTS radical cation (ABTS+) stock solution was generated by mixing 0.4 mL of ABTS aqueous solution (7.4 mM) with 1.43 mL of potassium persulfate (K_2_S_2_O_8_) solution (2.6 mM). The mixture was incubated in the dark at room temperature for 12 h. Prior to the assay, the ABTS∙+ stock solution was diluted with PBS to obtain an initial absorbance of 0.70 ± 0.02 at 734 nm.

To determine the scavenging activity, 50 μL of the PDNVs sample (SL-EXO or RP-EXO) at varying protein concentrations (0.05, 0.1, 0.2, 0.3, 0.4, and 0.5 mg/mL) was thoroughly mixed with 200 μL of the diluted ABTS working solution in a 96-well plate. For the control group (A_0_), 50 μL of PBS was used to replace the sample. After incubation for 10 min, the absorbance of the mixture was measured at 734 nm using a microplate reader. All experiments were performed in independent triplicate.

The ABTS radical scavenging rate was calculated using the following equation:

(1) $$\mathrm{Free}\;\mathrm{radical}\;\mathrm{scavenging}=A_{0}-A/A_{0}\times 100\%$$

where $A_{0}$ is the absorbance of the control, and A is the absorbance of the sample.

### 2.5. Tyrosinase Inhibition Assay

The in vitro tyrosinase inhibitory activities of SL-EXO, RP-EXO, and the positive control α-arbutin (0.06–0.4 mg/mL) were evaluated using an L-tyrosine oxidation assay. Briefly, samples were incubated with L-tyrosine and tyrosinase at 37 °C. The absorbance of the enzymatic product was measured at 475 nm. Detailed protocols are provided in the Supplementary Information (Method S2, Table S1) [17].

### 2.6. In Vivo Anti-Inflammatory Assay in Transgenic Zebrafish

#### 2.6.1. Zebrafish Culture

Adult wild-type zebrafish or Transgenic neutrophil fluorescent zebrafish embryos Tg(lyz:eGFP) were obtained from Nanjing YSY Biotechnology Co., Ltd. (Nanjing, China). and maintained in recirculating systems at 28.5 ± 1 °C and pH 8.0 ± 0.5. To produce embryos, 10 breeding groups with a male-to-female ratio of 1:2 were established. Only embryos with a fertilization rate greater than 80% were used to ensure reproducibility.

All zebrafish experiments were conducted with the approval of the Ethics Committee of Jiangxi Medical College (approval number ZE20250413, dated 13 April 2025). All procedures were performed in accordance with institutional regulations and the 3Rs principles, and every effort was made to minimize animal suffering and the number of animals used.

#### 2.6.2. Zebrafish Survival Assay

Healthy zebrafish embryos were randomly distributed into a 12-well plate (10 embryos per well). Each well contained 2 mL of medium supplemented with samples at final concentrations of 0.00001, 0.0005, 0.001, 0.002, 0.004, and 0.008 mg/mL. A control group received a medium without any sample. Viability and morphological abnormalities were recorded daily for 120 h.

#### 2.6.3. Fluorescent Neutrophil Anti-Inflammatory Model

The in vivo anti-inflammatory effects of SL-EXO and RP-EXO (0.0001–0.001 mg/mL, based on protein concentration) were tested in transgenic neutrophil-fluorescent zebrafish embryos [Tg(lyz:eGFP)]. Ibuprofen was used as a positive control. Lipopolysaccharide (LPS, 0.5 μg/mL) was used to induce inflammation. After a 96 h Incubation, the attenuation of neutrophil recruitment was quantified using ImageJ (ImageJ 1.54d), measuring the decrease in green fluorescence intensity [18]. The supplementary information (Method S3) contains detailed protocols for culturing, grouping, and modeling.

### 2.7. Zebrafish Anti-Aging Model

#### 2.7.1. Zebrafish Culture

Refer to Section 2.6.1.

#### 2.7.2. Zebrafish Survival Assay

Refer to Section 2.6.2.

#### 2.7.3. Anti-Aging Assays

To evaluate the in vivo microenvironment-modulating capacity of SL-EXO, two chemical-induced zebrafish models were used, with resveratrol as the positive control. First, an oxidative stress model was induced by H_2_O_2_ to quantify intracellular ROS levels (via DCFH-DA), SOD activity, and MDA content. Second, a premature senescence model was established using doxorubicin to assess senescence-associated β-galactosidase (SA-β-gal) activity. Staining and fluorescence were quantified via ImageJ. Detailed modeling, grouping, and assay protocols are provided in the supplementary information (Method S4) [19,20].

### 2.8. Zebrafish Skin-Whitening Assay

#### 2.8.1. Zebrafish Culture

Refer to Section 2.6.1.

#### 2.8.2. Zebrafish Survival Assay

Refer to Section 2.6.2.

#### 2.8.3. Zebrafish Whitening Assay

Wild-type zebrafish embryos (starting at 12 hpf) were treated with SL-EXO or the positive control α-arbutin at concentrations of 0.0001, 0.0005, and 0.001 mg/mL. Macroscopic pigmentation was documented microscopically after 96 h of exposure. To quantify melanin content and tyrosinase activity, embryos treated for 48 h were homogenized, and lysates were subjected to alkaline solubilization (measured at 405 nm) and L-DOPA oxidation (measured at 475 nm), respectively. Detailed procedures are provided in the supplementary information (Method S5) [21].

### 2.9. Multi-Omics Profiling of SL-EXO and RP-EXO

#### 2.9.1. Metabolomics Analysis

The lyophilized samples were extracted in 70% aqueous methanol. The supernatant was analyzed using the UPLC-ESI-QTRAP-MS/MS system (ExionLC™ AD coupled to a SCIEX QTRAP, Framingham, MA, United States). It was fitted with the Agilent SB-C18 column. Identification and quantification of metabolites were performed using Multiple Reaction Monitoring (MRM) and the Metware Database (MWDB). Statistical screening for differential metabolites between groups was performed using PCA and OPLS-DA with a strict fold-change threshold (≥2 or ≤0.5). The Supplementary Information contains detailed extraction protocols and instrumental parameters (Method S6) [22,23,24].

#### 2.9.2. Proteomics Analysis

Total proteins were extracted, quantified via a BCA assay, reduced, alkylated, and digested overnight with trypsin. The resulting desalted peptides were separated using a Vanquish Neo UHPLC system and analyzed in data-independent acquisition (DIA) mode on an Orbitrap Astral mass spectrometer (Thermo Scientific). Raw data were processed using DIA-NN (v1.8.1) in library-free mode with the Match Between Runs (MBR) feature enabled, searching against the Uniprot database. Exhaustive sample preparation, instrumental, and bioinformatic parameters are detailed in the supplementary information (Method S7) [25,26,27].

#### 2.9.3. Lipidomics Analysis

Lyophilized samples were extracted using a methyl tert-butyl ether (MTBE) and methanol solvent mixture. The lipid extracts were analyzed on an ExionLC™ AD UPLC system equipped with a Thermo Accucore C30 column, coupled to a SCIEX QTRAP 6500+ mass spectrometer (SCIEX, Framingham, MA, United States). Lipids were identified against the Metware Database (MWDB) and quantified via the internal standard method using multiple reaction monitoring (MRM). Exhaustive sample preparation protocols and instrumental parameters are detailed in the Supplementary Information (Method S8) [28,29,30].

### 2.10. Network Pharmacology Analysis

Potential targets of the active constituents were predicted using SwissTargetPrediction, while pigmentation-related disease targets were retrieved from the GeneCards and DisGeNET databases. The intersecting genes were identified as potential therapeutic targets and used to construct a drug–component–target network via Cytoscape. Protein–protein interaction (PPI) networks were built utilizing the STRING database and topologically analyzed with the CytoNCA plugin. Finally, Gene Ontology (GO) and KEGG pathway enrichment analyses were performed using the DAVID. Detailed database parameters and inclusion criteria are provided in the Supplementary Information (Method S9) [31,32,33].

### 2.11. B16F10 Cell Whitening Experiment

#### 2.11.1. Cell Culture

The B16F10 murine melanoma cell line was sourced from the National Collection of Authenticated Cell Cultures (Shanghai, China) and cultured in DMEM supplemented with 10% FBS and 1% antibiotic–antimycotic at 37 °C, 5% CO_2_, in a humidified atmosphere. Cells between passages 10 and 25 were used for all experiments.

#### 2.11.2. Cell Viability Assay

B16F10 cells were treated with SL-EXO, RP-EXO, and positive control α-arbutin at different concentrations (0.0025–0.15 mg/mL) for 24 h and 48 h. Cell viability was assessed using the Cell Counting Kit-8 (CCK-8) assay, and absorbance was measured at 450 nm. The Supplementary Information section contains cell culture protocols and the formula for calculating viability (Method S10).

#### 2.11.3. Cellular Uptake

The cellular internalization of SL-EXO and RP-EXO and α-arbutin by B16F10 cells was visualized using confocal laser scanning microscopy (CLSM, Olympus Corporation, Tokyo, Japan). Vesicles were first labeled with the green lipophilic dye PKH67. B16F10 cells were then incubated with the labeled vesicles for 24 h. Subsequently, cells were fixed, permeabilized, and counterstained with Alexa Fluor 488-phalloidin (for F-actin) and DAPI (for nuclei) before imaging. A detailed staining protocol, including dye concentrations and incubation times, is provided in the Supporting Information (Method S11) [34].

#### 2.11.4. Determination of Melanin Content and Tyrosinase Activity in B16F10 Cells

For anti-melanogenic evaluation in B16F10 melanocytes, in which 200 nM α-MSH was pre-treated for 1 h, and the cells were exposed for 48 h to nanovesicles or α-arbutin at a concentration of 0.0025–0.025 mg/mL. The intracellular tyrosinase activity was measured by L-DOPA oxidation at 475 nm. The values were normalized to the total protein concentrations to account for viability. Cellular melanin content was evaluated using spectrophotometry at 405 nm after alkaline solubilization. Detailed lysis and assay protocols are present in the Supplementary Information (Method S12) [21].

### 2.12. Western Blot Analysis

To confirm the mechanisms within the cell, B16F10 cells were divided into four groups: Control, Model (α-MSH-stimulated), Drug (co-treated with 0.025 mg/mL SL-EXO), and Rescue (pre-treated with 10 μM 740 Y-P, a PI3K/AKT agonist, and then treated with SL-EXO). Proteins were extracted using RIPA buffer and quantified using the BCA assay. Standard SDS-PAGE and immunoblots analyzed target proteins. We visualized the bands by enhanced chemiluminescence (ECL) and quantified the results in ImageJ (1.54d), using β-actin as a loading control [35,36]. Supplementary Information outlines detailed protocols (Method S13) and raw Western blot data (Figure S6).

### 2.13. Quantitative Real-Time PCR (RT-qPCR) Analysis

Total RNA from treated B16F10 cells was extracted using TRIzol reagent and reverse-transcribed into cDNA. Quantitative real-time PCR (RT-qPCR) was subsequently performed to evaluate the mRNA transcription levels of MITF, TYR, and TRP-1. Relative gene expression was calculated using the 2^−ΔΔCt^ method, with β-actin serving as the internal reference. Detailed thermal cycling protocols and primer sequences (Table S9) are provided in the Supplementary Information (Method S14) [37].

### 2.14. Molecular Docking and Interaction Visualization

To predict the structural binding affinities, molecular docking simulations were performed between the hyper-accumulated metabolites of SL-EXO and the core target AKT1. Briefly, ligand and receptor structures were preprocessed, and docking was executed using AutoDock Vina (version 1.2.5). The optimal conformations were evaluated based on binding free energy (ΔG, kcal/mol) and visually rendered via PyMOL (PyMOL 2.6) and Discovery Studio. Detailed parameters are provided in the Supplementary Information (Method S15) [38].

### 2.15. Statistical Analysis

The experimental procedures were performed in triplicate and reported as mean ± SD. Statistical analyses and data plotting were performed using GraphPad Prism 10 (GraphPad Software, Boston, MA, USA) and Origin 2019b (OriginLab Corporation, Northampton, MA, USA). The statistical significance was labelled as * *p* < 0.05, ** *p* < 0.01, *** *p* < 0.001, **** *p* < 0.0001 in the study.

## 3. Results and Discussion

### 3.1. Isolation and Physicochemical Characterization of RP-EXO and SL-EXO

**Figure 1:**
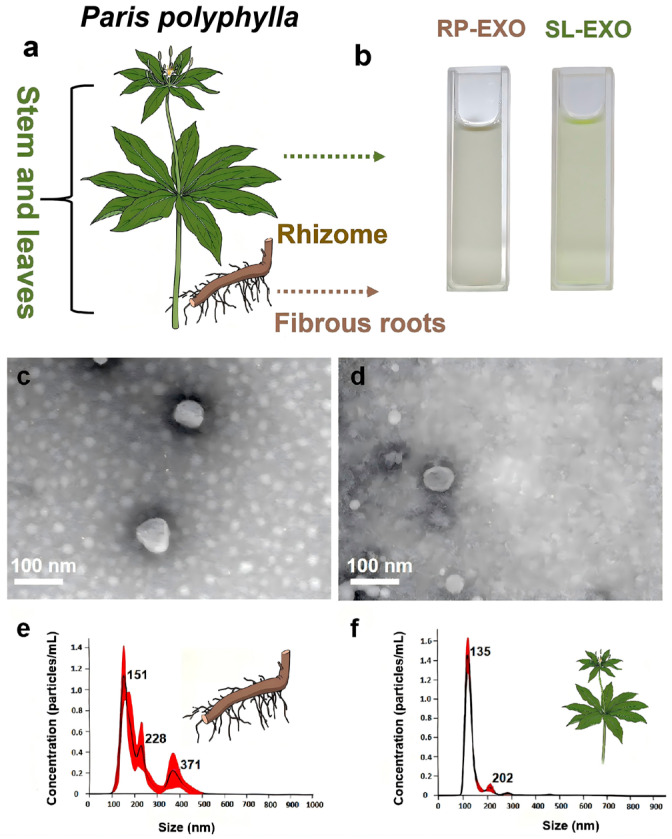
Physicochemical characterization of RP-EXO and SL-EXO. (**a**) Source tissues of *Paris polyphylla* for vesicle isolation. (**b**) Photographs of RP-EXO and SL-EXO colloidal suspensions. (**c**,**d**) TEM images demonstrating the vesicular structures of (**c**) RP-EXO and (**d**) SL-EXO. (**e**,**f**) Size distribution and particle concentration of (**e**) RP-EXO and (**f**) SL-EXO analyzed by NTA. In the NTA profiles, the red shaded area under each curve represents the size distribution of particles, with the peak indicating the most abundant particle size in the sample.

**Scheme 1:**
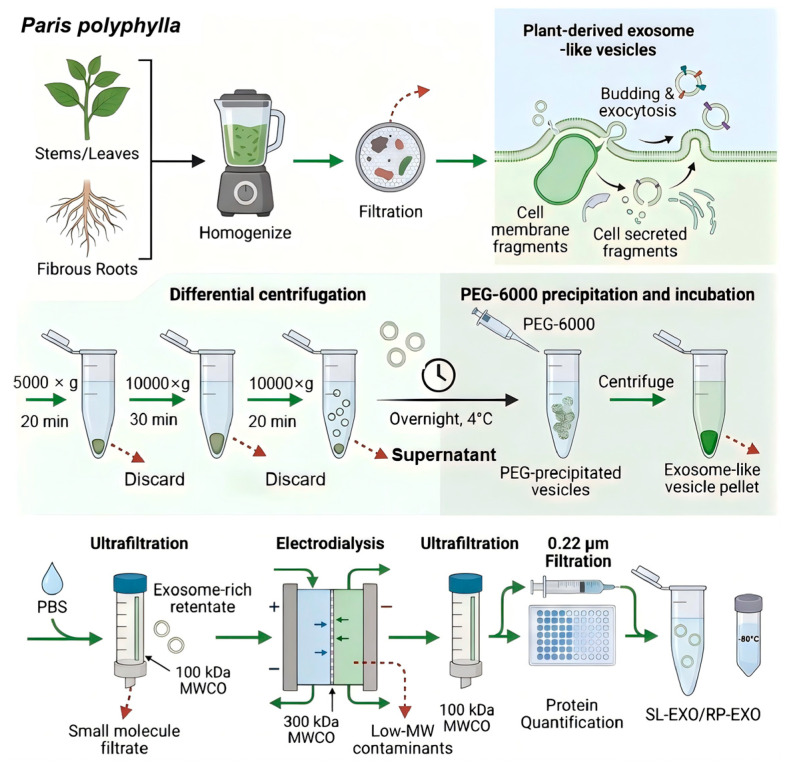
Schematic diagram of the preparation process of *Paris polyphylla* stems/leaves (SL-EXO) and fibrous roots (FR-EXO)-derived nanovesicles.

To achieve the valorization of agricultural waste from *Paris polyphylla*, plant-derived nanovesicles (PDNVs) were successfully isolated from the discarded non-medicinal parts, specifically the stems/leaves (SL-EXO) and fibrous roots (RP-EXO) (Figure 1a). As illustrated in Scheme 1, the preparation of the nanovesicles was conducted following our previously developed method, which combines polyethylene glycol (PEG) precipitation with electrophoretic dialysis [15]. The purified RP-EXO and SL-EXO suspensions appear macroscopically colloidal (Figure 1b). Significantly, the SL-EXO suspension was yellowish-green while the RP-EXO suspension was almost colorless. This visual variation may have originated from differences in the inherent phytochemistry of tissues. Aerial parts (stems and leaves) are pigmented in photosynthetic pigments (e.g., chlorophyll remnants) and have high levels of flavonoid compounds. These may be partially co-assembled or enwrapped within lipid bilayers during vesicular assembly, a phenomenon expected for leaf-derived nanovesicles.

To physically validate the purification efficiency of our PEG coupled with electrodialysis (PEG + ED) combinatorial method, the total protein concentration of the retentate was dynamically monitored during the purification process. As shown in Figure S1a–c, the protein concentration progressively decreased and plateaued by the fifth 30 min cycle, confirming the successful removal of co-precipitated extravesicular free proteins. Furthermore, we compared the PEG + ED isolated PDNVs (SL-EXO) with those isolated via gold-standard ultracentrifugation (UC). The NTA size distribution of the UC-obtained PDNVs exhibited a relatively broader peak with a mean diameter of ~160 nm (Figure S1e), which may indicate vesicle aggregation or coalescence induced by high-speed shear forces. In contrast, our PEG + ED method produced a sharper and more uniform peak (~135 nm), suggesting superior preservation of vesicle structural integrity, while concurrently achieving a substantially higher yield and requiring significantly less processing time than UC.

Transmission electron microscopy (TEM) was used to visualize the ultrastructural morphology of isolated nanovesicles. As illustrated in Figure 1c,d. Both RP-EXO and SL-EXO had intact vesicles on their membrane. The vesicles mainly exhibited a classic “cup-shaped” or spherical morphology. The cup-like indentation is frequently observed and results from vesicle deflation and dehydration induced by negative staining required for transmission electron microscopy (TEM) preparation, confirming the integrity of these isolated PDNVs, which are lipid bilayer structures [39].

**Figure 2:**
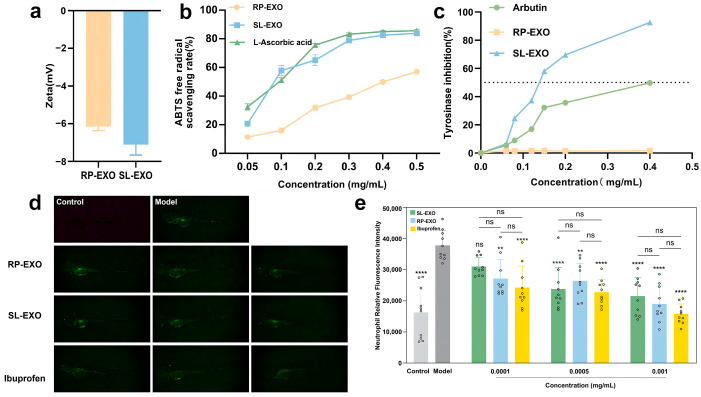
Physicochemical stability and initial in vitro/in vivo efficacy screening of tissue-specific nanovesicles (SL-EXO and RP-EXO). (**a**) Zeta potential measurements of SL-EXO and RP-EXO. (**b**) In vitro ABTS radical scavenging capacity of SL-EXO and RP-EXO at varying concentrations (0.05–0.5 mg/mL), with L-ascorbic acid serving as the positive control. Green triangles represent ascorbic acid, blue squares represent SL-EXO, and orange circles represent RP-EXO. (**c**) In vitro tyrosinase inhibitory activity profiles across different concentrations (0.06–0.40 mg/mL), utilizing α-arbutin as the reference standard. (**d**) Representative in vivo fluorescence images of transgenic neutrophil-fluorescent zebrafish embryos. (**e**) Quantitative analysis of the relative inflammatory fluorescence intensity at the injury site corresponding to the zebrafish models in (**d**). Data are presented as the mean ± SD (*n* ≥ 3). Statistical significance is denoted as ** *p* < 0.0 and **** *p* < 0.0001 compared to the corresponding model.

To quantitatively evaluate the size distribution and particle concentration in an aqueous environment, Nanoparticle Tracking Analysis (NTA) was performed (Figure 1e,f). NTA revealed that the average particle sizes of SL-EXO and RP-EXO were 140.6 ± 2.6 nm and 186.0 ± 5.8 nm, respectively, and distinct size distribution patterns between the two groups. The final concentrations of the isolated nanovesicles were determined to be 1.44 × 10^11^ particles/mL for SL-EXO and 9.81 × 10^10^ particles/mL for RP-EXO (Figure S2). Both nanovesicles exhibited negative surface charges, with Zeta potentials of −7.10 mV for SL-EXO and −6.15 mV for RP-EXO (Figure 2a). Additionally, from a physical chemistry perspective, the measured Zeta potentials of SL-EXO (approximately −6 to −7 mV) indicate relatively weak electrostatic repulsion in pure aqueous suspension, which may theoretically predispose the vesicles to aggregation during long-term storage. While the current study primarily focuses on establishing the extraction methodology and decoding the molecular anti-melanogenic mechanisms, unlocking the true industrial potential of SL-EXO requires further formulation science. Future studies will focus on incorporating SL-EXO into finalized cosmetic matrices (e.g., emulsions or creams) and systematically evaluating their long-term colloidal stability, rheology, and sustained bioactivity under industrial storage conditions.

The two types of tissue vary in size and homogeneity, a noticeable characteristic. The increased heterogeneity and greater average size of RP-EXO, compared to SL-EXO, could be due to the more complex physiological environment and thicker cell walls of root tissues. Such differences in subcellular compartments, along with an endomembrane trafficking system distinct from that of leaves, may promote secretion of a broader range of multivesicular bodies. Alternatively, root-specific secondary metabolites (such as abundant saponins or mucilage in *P. polyphylla* roots) may induce slight vesicle aggregation during isolation [40].

It should be noted that the NTA-measured particle sizes (e.g., 140.6 ± 2.6 nm and 186 ± 5.8 nm) were greater than those observed in the TEM images (<100 nm). This situation is biological and logical because NTA essentially measures the hydrodynamic diameter of these fully hydrated vesicles that move in solution (including the hydration layer and all corona on the surfaces), while TEM measurements are made under vacuum, reflecting the dehydrated, shrunken state of the particles.

However, despite this significant purification enhancement of PEG coupled with the electrodialysis (PEG + ED) combinatorial method, no current methodology (including our PEG + ED approach) can guarantee 100% absolute purity. We must objectively acknowledge that trace amounts of tightly bound extravesicular contaminants might still co-exist. Furthermore, our ED method presents a specific technical limitation: the applied electric current inevitably generates Joule heating during dialysis. To prevent thermal degradation of the vesicles, the entire ED apparatus must be strictly cooled (e.g., submerged in ice packs) to maintain low temperatures.

### 3.2. Initial Efficacy Screening: Antioxidant, Anti-Tyrosinase, and Anti-Inflammatory Correlative Bioactivities

Skin hyperpigmentation is a multifactorial pathological process primarily driven by chronic oxidative stress, elevated tyrosinase catalytic activity, and subclinical inflammatory cascades (often manifesting as post-inflammatory hyperpigmentation, PIH) [41]. Therefore, a comprehensive initial screening evaluating free radical scavenging, direct enzyme inhibition, and in vivo immunomodulation is the paramount first step in identifying potential hypopigmenting candidates.

We initially evaluated the biochemical activities of the tissue-specific PDNVs. The ABTS radical scavenging assay (Figure 2b) immediately revealed a sharp contrast. At the highest concentration (0.5 mg/mL), RP-EXO displayed slow antioxidant kinetics, reaching only 57.00%. In a stark contrast, SL-EXO showed exceptional and dose-dependent antioxidant activity. SL-EXO at 0.1 mg/mL reached a percent scavenging of 57.80%, demonstrating robust free-radical quenching capabilities. Eventually, at 0.5 mg/mL, SL-EXO displayed a percent scavenging of 83.83 ± 0.95%. L-ascorbic acid, utilized here strictly as a methodological positive control to validate assay sensitivity, yielded an expected scavenging plateau (85.63 ± 0.91% at 0.5 mg/mL), thereby confirming the reliability of our testing system.

This remarkable ability of the SL-EXO to quench ROS entails a robust capacity to intercept oxidative cascades required for tyrosinase activation. An in vitro tyrosinase inhibition assay was performed incorporating α-arbutin as the reference standard to ensure assay functionality (Figure 2c) to directly confirm this observation. The findings were decidedly clear-cut: RP-EXO showed practically total absence of anti-tyrosinase activity, throughout the tested range (which stagnated below 2.0%). Conversely, SL-EXO showed remarkable, dose-dependent inhibitory efficacy that far exceeded that of α-arbutin, the gold standard. For example, the inhibition rate of SL-EXO at 0.15 mg/mL was 58.08%, indicating substantial enzymatic suppression. At the highest concentration evaluated (0.40 mg/mL), the SL-EXO system delivered almost total inhibition of tyrosinase activity (92.66%). Simultaneously, the pure chemical standard α-arbutin produced a predictable concentration-dependent inhibition curve (reaching 49.78% at 0.40 mg/mL), validating the experimental paradigm. It is important to note that direct molar-equivalent comparisons between a multi-component botanical suspension (quantified by bulk protein mass) and a single-molecule entity (α-arbutin) are analytically divergent; nevertheless, the data unequivocally confirm the profound intrinsic bioactivity of SL-EXO.

From the point of view of plant physiology, the aerial parts (stems/leaves) of *Paris polyphylla* are constantly exposed to UV radiation, which has prompted the evolution of these species to produce certain antioxidants (such as flavonoids and polyphenols) to prevent photo-oxidative damage. It is presumed that these metabolites are embedded into SL-EXO, which acts as nanoscopic biomolecular depots to simultaneously quench ROS and repel the active site of tyrosinase. Mechanistically, it is crucial to determine whether this pronounced cell-free anti-tyrosinase effect arises from specific active-site inhibition or nonspecific interference. While the complex nanovesicle structure (lipids and proteins) may exert some non-specific steric hindrance or protein denaturation effects in the in vitro assay buffer, the primary inhibitory driver is anticipated to be highly specific. As extensively documented in the literature, the phytochemicals hyper-accumulated in aerial plant tissues, particularly flavonoid glycosides and phenolic acids, function as potent competitive inhibitors [42]. Their aromatic rings and abundant hydroxyl (-OH) groups allow them to directly chelate the binuclear copper (Cu^2+^) center residing within the tyrosinase active pocket, structurally paralyzing the enzyme’s catalytic capability. The roots (RP) that live in the dark are missing these anti-melanogenic molecules [43].

Beyond direct enzyme inhibition, mitigating the inflammatory microenvironment is crucial for preventing melanocyte hyperactivation [41]. To this end, a transgenic fluorescent zebrafish model of neutrophils was employed, with ibuprofen as the positive control (Figure 2d,e). Following injury, the model group exhibited massive neutrophil recruitment to the inflammatory site, with relative fluorescence intensity spiking to 37,809 (compared to 15,833 in the control).

Interestingly, following nanovesicle interventions, both SL-EXO and RP-EXO demonstrated discernible and dose-dependent immunomodulatory effects. At the highest dosage (0.001 mg/mL), RP-EXO and SL-EXO successfully suppressed the inflammatory fluorescence intensity down to 19,039 and 21,530, respectively, both approaching the therapeutic efficacy of ibuprofen (15,822). This suggests that fundamental structural components common to plant nanovesicles (such as specific membrane lipids or ubiquitous plant sterols) may possess intrinsic anti-inflammatory properties, enabling both root- and leaf-derived vesicles to curtail neutrophil migration.

While RP-EXO exhibited commendable anti-inflammatory properties, it failed to meet the prerequisites for direct tyrosinase inhibition and ROS scavenging. SL-EXO, on the other hand, came out as a versatile “all-rounder”. SL-EXO is a strong treatment for hyperpigmentation as it can disrupt the tri-pillar microenvironment of hyperpigmentation by simultaneously (1) counteracting oxidative stress, (2) strongly inhibiting tyrosinase activity, and (3) effectively resolving inflammation. Thus, the deep mechanistic omics investigation followed by the in vivo cellular evaluation is unequivocally justified by the emergence of SL-EXO as the primary bioactive candidate through a rigorous multidimensional screening analysis.

### 3.3. SL-EXO Ameliorates the Pigmentation-Driving Oxidative and Senescent Microenvironment In Vivo

Accumulating dermatological evidence indicates that prolonged oxidative stress and cellular senescence are not merely signs of aging, but are intrinsic drivers of melanocyte hyperactivation. Senescent cells secrete senescence-associated secretory phenotypes (SASP), while excess ROS directly provides the oxidative condition required for tyrosinase catalysis [44]. Therefore, rectifying this pathological microenvironment is a prerequisite for profound depigmentation. To validate the in vivo microenvironment-modulating capability of SL-EXO, a chemical-induced oxidative stress and premature aging zebrafish model was utilized, with the well-documented polyphenol resveratrol serving as the positive benchmark.

**Figure 3:**
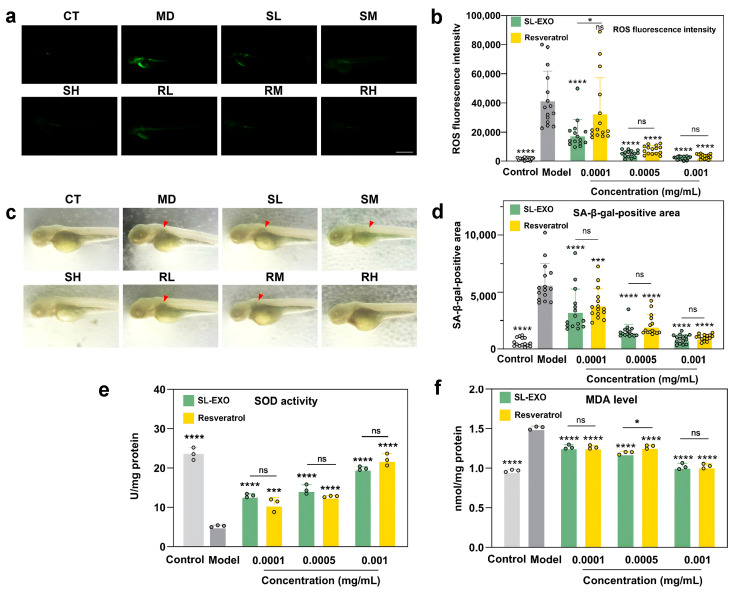
In vivo amelioration of the oxidative and senescent microenvironment in zebrafish by SL-EXO. (**a**) Representative fluorescence images showing intracellular reactive oxygen species (ROS) accumulation in zebrafish embryos across different treatment groups. (**b**) Quantitative analysis of the whole-body ROS fluorescence intensity corresponding to (**a**). (**c**) Representative stereomicroscopic images of senescence-associated β-galactosidase (SA-β-gal) staining in zebrafish embryos. The red arrow indicates the β-galactosidase staining region in zebrafish embryos. The intense blue color indicates cellular senescence. Scale bar (white line) = 250 μm. (**d**) Quantitative statistical analysis of the SA-β-gal-positive staining area corresponding to (**c**). (**e**) Determination of in vivo superoxide dismutase (SOD) enzymatic activity and (**f**) quantification of malondialdehyde (MDA) levels in the whole-body lysates of zebrafish. In all assays, a chemical-induced oxidative stress/aging model was utilized, with Resveratrol serving as the positive therapeutic control. Data are presented as the mean ± SD (*n* ≥ 3). Statistical significance is denoted as * *p* < 0.05, *** *p* < 0.001, and **** *p* < 0.0001 compared to the Model group. The T-shaped lines represent error bars (SD). The dots represent individual replicate data points. The ‘ns’ indicates no significant difference between the two compared groups (*p* > 0.05).

Cellular Overproduction of ROS is the primary reason for the damage caused by oxidation Fluorescence imaging (Figure 3a) and subsequent quantification (Figure 3b) confirmed that the model group experienced a systemic and robust upsurge in ROS fluorescence intensity (the mean value rose to around 42,000 with respect to the baseline value of 1500 in the control group), confirming a serious oxidative stress condition in the model. Obviously, this spinoff SL-EXO intervention acts in a dose-dependent manner to dramatically inhibit ROS accumulation. At the highest dosage (0.001 mg/mL), SL-EXO suppressed ROS fluorescence intensity to near-basal levels (mean ≈ 2100), demonstrating quenching efficacy comparable to, and in some cases slightly superior to, the corresponding resveratrol dose (mean ≈ 3300). At the same time, we measured senescence-associated β-galactosidase (SA-β-gal), a universal histochemical marker of senescence (Figure 3c,d). The model embryos showed strong, extensive blue staining along the body axis (mean positive area ≈ 5700), indicating pronounced premature senescence. Treatment with SL-EXO significantly and dose-dependently conditioned the SA-β-gal-positive area. Most importantly, SL-EXO cleared all senescent cells (positive area decreased to ≈880) at 0.001 mg/mL, consistent with purified resveratrol, a positive control.

To further elucidate the biochemical nature of the aforementioned visual phenotypes, we determined the levels of superoxide dismutase (SOD, the first line endogenous antioxidant enzyme) and malondialdehyde (MDA, a principal biomarker of lipid peroxidation). In line with the imaging data, the model group exhibited a severe reduction in SOD activity (55.28 U/mg protein) and a significant increase in MDA levels (1.51 nmol/mg protein) (Figure 3e,f). Redox homeostasis in the body was restored by SL-EXO treatment. The elevated SOD activity (19.92 U/mg protein) by SL-EXO at a high dose of 0.001 mg/mL was near the normal level (23.63 U/mg protein) and that of resveratrol (22.13 U/mg protein). Consequently, the content of MDA under the influence of SL-EXO was reduced to 1.02 nmol/mg protein.

The strong in vivo antioxidant and anti-senescence activity of SL-EXO provides a clear confirmation of our previous in vitro ABTS results. In vivo, small molecules that work for free are often rapidly metabolized and have poor bioavailability. Such problems do not seem to arise with SL-EXO because it is an evolutionarily slow, specially designed, smart nanocarrier. The biomimetic lipid bilayer of PDNVs protects catalpol, delphinidin, and chromium picolinate from premature degradation and enables their highly efficient transcellular penetration across zebrafish mucosal barriers. Moreover, SL-EXO effectively removes ROS and reduces senescent cells, thereby clearing systemic oxidative stress and SASP signals. This mechanism basically disrupts the pathological microenvironment needed for melanogenesis. Thus, a strong case can be made for the next step, namely, the assessment of its direct in vivo and in vitro depigmenting efficacy.

### 3.4. In Vivo Anti-Melanogenic Efficacy: Phenotypic Eradication and Biochemical Validation in Zebrafish

The pigmented characters of zebrafish and its phenotypes suggest it has human tyrosinase orthologs. The transparency and rapid post-birth development of these organisms make them a perfect model for testing skin-whitening agents [45]. In light of the convincing evidence in Section 3.3 that SL-EXO effectively neutralizes the microenvironment that drives pigmentation via oxidative and senescent mechanisms, we further investigated the ultimate therapeutic effect of SL-EXO on physiological melanin synthesis. The commercial α-arbutin used was positive control.

**Figure 4:**
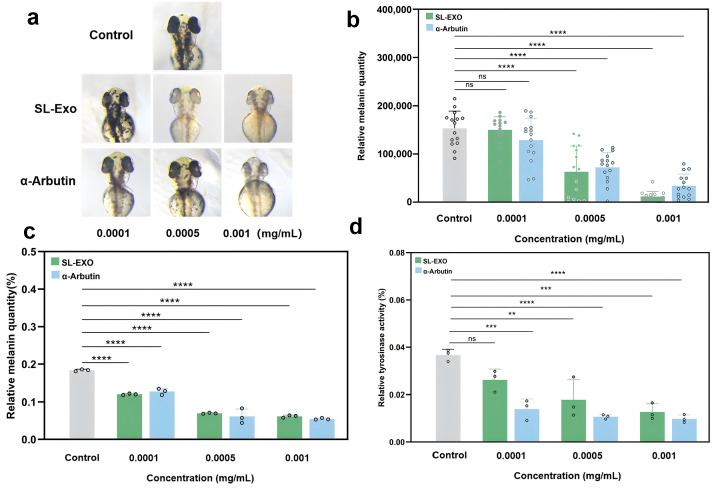
In vivo suppression of melanogenesis by SL-EXO in the zebrafish model. (**a**) Representative macroscopic stereomicroscopic images of zebrafish embryos. (**b**) Quantitative image analysis of the relative melanin pixel density corresponding to the visual observations in (**a**). (**c**) Biochemical quantification of the relative total intracellular melanin content extracted from whole-body zebrafish lysates. (**d**) Determination of the relative in vivo tyrosinase catalytic activity from zebrafish tissue homogenates. In all in vivo depigmentation assays, α-arbutin was employed as the commercial positive control. Data are expressed as the mean ± SD (*n* ≥ 3). Statistical significance is indicated as ** *p* < 0.01, *** *p* < 0.001, and **** *p* < 0.0001 compared to the untreated Control group.

Macroscopic and stereomicroscopic observations were first employed to monitor in vivo melanin accumulation. As depicted in Figure 4a, the control zebrafish embryos exhibited dense, dark pigment spots extensively distributed along their cranial, dorsal, and yolk sac regions. Following continuous aqueous exposure, a profound, dose-dependent abrogation of body pigmentation was visually evident in the SL-EXO-treated groups. Quantitative image analysis of the relative melanin pixel density (Figure 4b) corroborated these striking visual findings. In the control group, the relative melanin quantity averaged approximately 153,151. Remarkably, at the highest dosage (0.001 mg/mL), SL-EXO almost completely eradicated the macroscopic melanin deposits, plummeting the pixel density to roughly 11,690. This phenomenal visual clearance was not only statistically highly significant but also visually outcompeted the pigment-clearing effect observed in the equivalent α-arbutin group (mean density ≈ 33,630). This suggests an extraordinarily efficient transepithelial delivery of SL-EXO in vivo.

To eliminate potential optical biases arising from melanin aggregation versus actual synthesis, total intracellular melanin and in vivo tyrosinase catalytic activity were biochemically extracted and quantified from whole-body lysates. Consistent with the morphological observations, SL-EXO treatment triggered a drastic, concentration-dependent decline in total melanin content (Figure 4c). At 0.001 mg/mL, SL-EXO suppressed the absolute melanin content to 0.061 (relative value), representing an approximate 66% absolute reduction compared to the basal control level (0.183), thereby achieving an overall depigmentation efficacy perfectly comparable to the purified α-arbutin (0.054).

Crucially, the mechanism underlying this pigmentary regression was validated in vivo by the tyrosinase activity assay (Figure 4d). The intrinsic tyrosinase activity in the SL-EXO-treated (0.001 mg/mL) zebrafish dropped to 0.0127, corresponding to an inhibition of 65.4% compared to the control (0.0367). Although the free small-molecule α-arbutin showed only slight inhibition of the enzyme in vivo (0.0098), SL-EXO demonstrated superior macroscopic clearance (Figure 4b) and thus a broader mechanism.

The translation of outstanding cell-free anti-tyrosinase activity into such remarkable in vivo depigmentation underscores the multifaceted superiority of plant-derived nanovesicles. The exceptional in vivo performance of SL-EXO can be attributed to two main dimensions:

Physicochemically, the biomimetic liposomal architecture of SL-EXO serves as a highly efficient delivery vehicle. It protects the encapsulated bioactive phytochemicals from degradation in the surrounding aqueous medium and facilitates superior trans-mucosal penetration across the zebrafish skin and gills, ensuring a high local concentration at the melanocyte target sites.

Biologically, the anti-melanogenesis mechanism of SL-EXO is highly synergistic. In contrast to a unique competitive tyrosinase inhibitor, α-arbutin, SL-EXO is a dual-action agent. It acts both on tyrosinase’s catalytic function (Figure 4d) and on diminishing the intracellular oxidative and senescent environment (as revealed by our ROS and SA-β-gal data). Melanin polymerization requires an oxidized state and, therefore, such a modulation will essentially paralyze the melanogenic machinery.

The tissue-specific efficacy of SL-EXO, compared with the inactivity of RP-EXO, raised the question of which specific molecules mediate this effect. To counter this, a multi-omics strategy was employed to interrogate SL-EXO content; the results are shown in subsequent sections.

### 3.5. Metabolomic Profiling Unveils the Phytochemical Payloads Dictating the Efficacy Dichotomy

The striking phenotypic differences detected in both in vitro enzyme assays and in vivo zebrafish models have brought our inquiry to a decisive molecular turning point. Accordingly, UPLC-ESI-MS/MS metabolomic profiling was applied to both SL-EXO and RP-EXO, with the dual objectives of uncovering the underlying material foundation and identifying the specific bioactive constituents present in the PDNVs.

**Figure 5:**
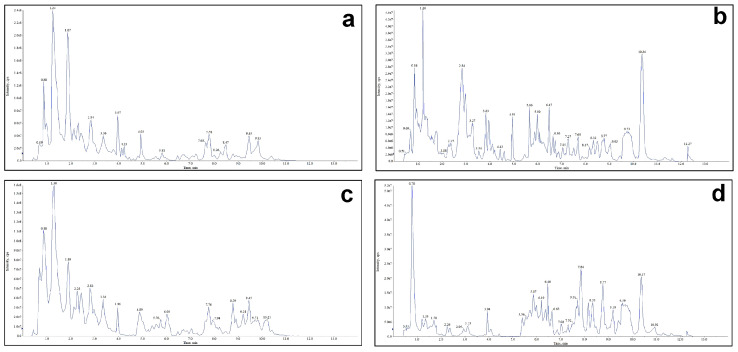
Global metabolic profiling of the tissue-specific nanovesicles via UPLC-ESI-MS/MS. Representative total ion chromatograms (TIC) for SL-EXO operating in (**a**) positive and (**b**) negative ion modes. Representative TIC for RP-EXO operating in (**c**) positive and (**d**) negative ion modes. “e” represents “× 10^”, i.e., “8e3” is equivalent to “8 × 10^3^”.

**Figure 6:**
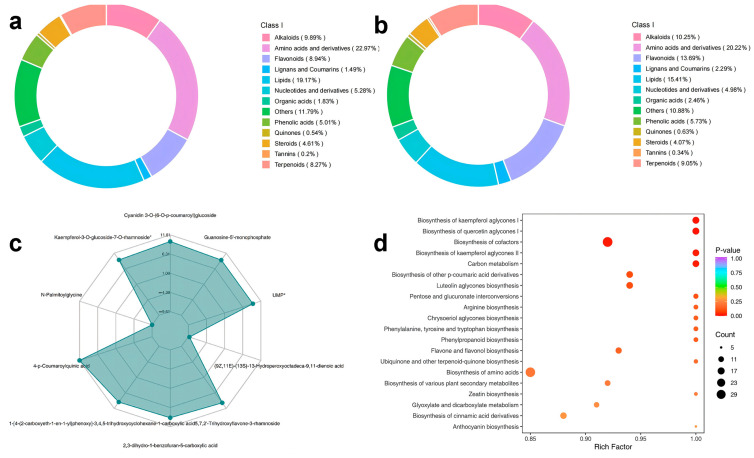
Differential metabolomic analysis uncovering the phytochemical payloads responsible for the efficacy dichotomy. Circular charts illustrating the comprehensive classification and relative proportion of identified metabolite categories within (**a**) RP-EXO and (**b**) SL-EXO. (**c**) A radar chart displaying the Top 10 differential metabolites with the maximum absolute Log2FC values between SL-EXO and RP-EXO. *: There are isomers present in this substance. (**d**) KEGG pathway enrichment bubble plot of the differentially accumulated metabolites (SL-EXO vs. RP-EXO). The color gradient indicates the *p*-value significance, while bubble size corresponds to the number of enriched metabolites. The analysis reveals a robust enrichment of secondary metabolite biosynthesis cascades in SL-EXO.

Total ion chromatograms (TICs) obtained in positive and negative ion modes indicated that the instrument performance remained stable and that the tissue-derived vesicles generated distinct, complex peak profiles (Figure 5a–d). The detected metabolites were classified into various phytochemical classes to assess their chemical architecture systematically (Figure 6a,b and Figure S3a). Despite both nanovesicles sharing a basic metabolic framework (e.g., amino acids, lipids, nucleotides, and other components essential for basic vesicular construction), their secondary metabolites were markedly different (Tables S2 and S3). Most importantly, the most effective natural antioxidants, named flavonoids, were represented at a content of 8.94% in RP-EXO from roots, while very high at 13.69% in SL-EXO from stem/leaf. Likewise, SL-EXO showed a notably higher proportion of phenolic acids and terpenoids. This differential allocation aligns perfectly with plant physiology: the aerial stems and leaves are continuously exposed to solar UV radiation, thereby driving the evolutionarily upregulation of the biosynthesis of photoprotective and antioxidant secondary metabolites. During biogenesis, these metabolites are selectively encapsulated into SL-EXO, endowing it with superior bioactivities.

To pinpoint the exact molecular effectors, a differential expression analysis was conducted (Figures S3b and S4). A radar chart (Figure 6c, Table S4) was generated from the top 10 metabolites with the highest absolute Log2FC values. Strikingly, 8 of the top 10 discriminative molecules were massively upregulated in SL-EXO, primarily flavonoid glycosides and phenolic acid derivatives.

The hyper-accumulated payloads in SL-EXO explicitly included Cyanidin 3-O-(6-O-p-coumaroyl)glucoside, Kaempferol-3-O-glucoside-7-O-rhamnoside, 5,7,2′-Trihydroxyflavone-3-rhamnoside, and 4-p-Coumaroylquinic acid. Addressing the aforementioned mechanistic inquiry, the specific tyrosinase-inhibitory capacity of SL-EXO is firmly corroborated by the presence of these exact molecules. In dermatological pharmacology, kaempferol derivatives and coumaric acids are renowned specific competitive inhibitors. Their molecular architecture—rich in aromatic rings and multiple hydroxyl (-OH) functional groups—enables them to directly and specifically chelate the binuclear copper (Cu^2+^) active site of the tyrosinase enzyme, independent of any generic vesicular interference. Their aromatic ring structures and multiple hydroxyl (-OH) functional groups allow them to efficiently scavenge ROS and directly chelate the binuclear copper (Cu^2+)^ active site of the tyrosinase enzyme. The enrichment of these specific compounds within SL-EXO provides molecular evidence for its extraordinary performance in the ABTS and tyrosinase inhibition assays observed in Section 3.2. Interestingly, nucleotide derivatives such as Guanosine-5′-monophosphate (GMP) and UMP were also significantly upregulated in SL-EXO, which may further contribute to cellular energy homeostasis and tissue repair.

To decode the underlying material basis responsible for the striking functional dichotomy between SL-EXO and RP-EXO at a systemic level, a KEGG pathway enrichment analysis of the differentially accumulated metabolites was executed (Figure 6d). The bubble plot revealed a massive and highly significant enrichment (high Rich Factor and low *p*-value) of secondary metabolite biosynthesis pathways in SL-EXO compared to RP-EXO.

The most prominent divergent pathways were robustly clustered around phenolic and flavonoid biosynthesis, including the biosynthesis of quercetin aglycones, kaempferol aglycones, luteolin aglycones, as well as broader phenylpropanoid and flavone/flavonol biosynthesis cascades. These phytochemical families are renowned for their exceptional redox-modulatory capacities. The specific hyper-accumulation of these flavonoid and phenolic pathways perfectly corroborates the robust ROS-scavenging efficacy and superior cell-free tyrosinase inhibition of SL-EXO. Furthermore, the significant enrichment in the “Biosynthesis of amino acids” and “Carbon metabolism” pathways suggests that the stems and leaves, as the primary photosynthetic and metabolically active aerial organs, endow SL-EXO with a richer, more structurally diverse biochemical repertoire than the fibrous roots.

Collectively, these metabolomic insights confirm that SL-EXO serves as a highly evolved natural biomimetic nanocarrier, inherently encapsulating a high-density payload of anti-melanogenic phytochemicals. This explicit molecular inventory paves the way for the subsequent network pharmacology analysis to map these specific ligands against human pigmentation targets.

### 3.6. Lipidomic Architecture: Chloroplast-Derived Biomimetic Vehicles for Superior Delivery

While metabolomics identified the active phytochemical payloads, the lipid bilayer of plant-derived nanovesicles plays an equally indispensable role. The lipid membrane not only delivers and shields the payloads but also controls the colloidal stability, cell internalization, and transdermal penetration efficiency of the nanovesicle. We performed a comparative lipidomic analysis to decode the structural advantages of SL-EXO.

**Figure 7:**
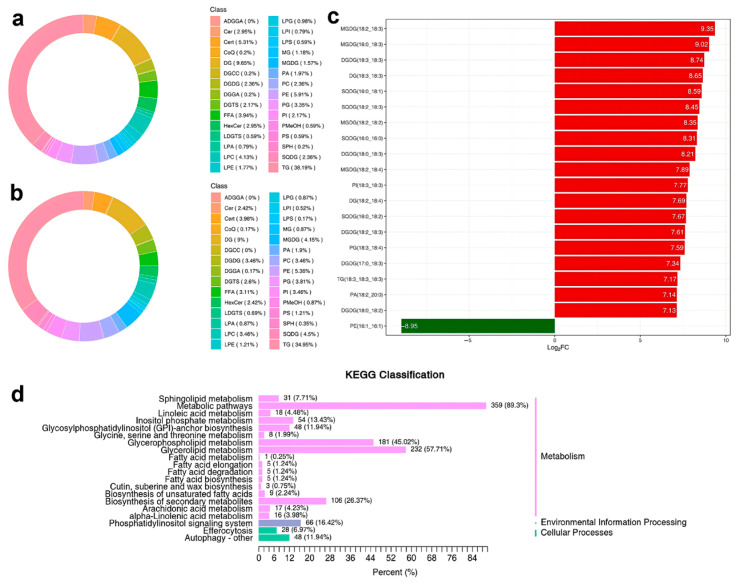
Comparative lipidomic profiling revealing the chloroplast-derived biomimetic architecture of SL-EXO. Circular charts demonstrating the comprehensive subclass composition and relative proportions of identified lipids within (**a**) RP-EXO and (**b**) SL-EXO. (**c**) A bar chart depicting the differential lipids between SL-EXO and RP-EXO (Log2FC absolute values). Red bars represent up-regulated metabolites, green bars represent down-regulated metabolites. (**d**) KEGG pathway classification of the differential lipids (SL-EXO vs. RP-EXO). The vertical axis shows the names of the KEGG metabolic pathways, and the horizontal axis represents the number and percentage of differential lipids annotated to each respective pathway.

Figure 7a,b and Table S5 display the lipid subclass profile of RP-EXO and SL-EXO on a global scale. The main structure of both nanovesicles comprises mainly neutral lipids like triglycerides (TG, ~34–38%) and diacylglycerols (DG, ~9%) as well as structural phospholipids like phosphatidylethanolamine (PE) and phosphatidylcholine (PC).

However, a significant tissue-specific disparity was observed in the glycolipid and sulfolipid fractions (Table S6). The relative amounts of monogalactosyldiacylglycerol, digalactosyldiacylglycerol, and sulfoquinovosyldiacylglycerol (MGDG, DGDG, and SQDG) in RP-EXO were quite low (1.57%, 2.36%, and 2.36%, respectively). On the contrary, these lipid classes grew tremendously in SL-EXO (4.15%, 3.46%, and 4.50%, respectively). Biologically, MGDG, DGDG, and SQDG are the signature, highly-conserved lipid constituents exclusive to the thylakoid membranes of plant chloroplasts [46]. The lipidomic analysis would confirm that SL-EXO most likely originates from the highly active light-driven endomembrane systems of the photosynthetic aerial stems and leaves, rather than from root-derived RP-EXO.

A differential Log2FC analysis was performed to emphasize the lipidomic differences (Figure 7c and Figure S5). The bar graph revealed a surprising upregulation of various galactolipids in SL-EXO. The uppermost 18 differentially enriched lipids (shown in red) exhibited large fold changes, with Log2FC values consistently exceeding 7.0. Remarkably, certain unsaturated galactolipids composed the top of this list, particularly MGDG(18:2_18:3), having a startling Log2FC of 9.35, as well as MGDG(16:0_18:3) (Log2FC = 9.02) and DGDG(18:3_18:3) (Log2FC = 8.74). On the other hand, only one phospholipid, PE(16:1_16:1), was significantly downregulated (Log2FC = −8.95) in SL-EXO.

The hyper-accumulation of MGDG and DGDG species endows SL-EXO with pharmacological advantages. In dermatological applications, plant galactolipids possess molecular structures highly analogous to the ceramides found in the human stratum corneum [47]. They act as exceptional natural surfactants and edge-activators, granting the nanovesicles remarkable deformability and membrane-fusion capabilities. This unique biomimetic lipid architecture perfectly explains our earlier observations: it provides a mechanistic rationale for both the superior trans-mucosal absorption seen in the in vivo zebrafish model (Figure 4) and the excellent, non-toxic biocompatibility observed in B16F10 melanoma cells, allowing SL-EXO to deliver high-dose phytochemicals without causing osmotic stress or cell death.

Beyond structural roles, specific lipids actively participate in cellular signaling. The KEGG classification of the differential lipids (Figure 7d) showed that 57.71% mapped to “Glycerolipid metabolism” and 45.02% to “Glycerophospholipid metabolism,” reflecting active vesicular membrane remodeling.

More intriguingly, the pathway analysis highlighted a notable enrichment in “Linoleic acid metabolism” (4.48%) and “alpha-Linolenic acid metabolism” (3.98%). As indicated by the fatty acid tails of the top upregulated MGDG/DGDG species (e.g., 18:2 and 18:3), SL-EXO is intrinsically rich in linoleic and linolenic acid moieties. In cutaneous biology, linoleic acid is a functional polyunsaturated fatty acid and a major component of skin surface free fatty acids; together with ceramides and cholesterol, it constitutes the stratum corneum lipid matrix and critically contributes to barrier integrity, lipid phase regulation, and local anti-inflammatory activity [48,49].

Collectively, the lipidomic data indicate that SL-EXO is more than an inert carrier; its chloroplast-derived, galactolipid-rich membrane serves a dual role: functioning as a biomimetic transdermal delivery vehicle, while its structural fatty acids synergize with flavonoid cargoes to exert potent anti-melanogenic effects.

### 3.7. Proteomic Landscape: The Structural Scaffold and Biosynthetic Fingerprint of SL-EXO

While lipids construct the vesicular vehicle and metabolites serve as the bioactive payloads, the membrane and luminal proteins constitute the indispensable structural scaffold and functional machinery of plant-derived nanovesicles [50]. To complete our multi-omics triad and elucidate the extreme efficacy dichotomy between the two tissue sources, a comprehensive proteomic profiling was executed.

**Figure 8:**
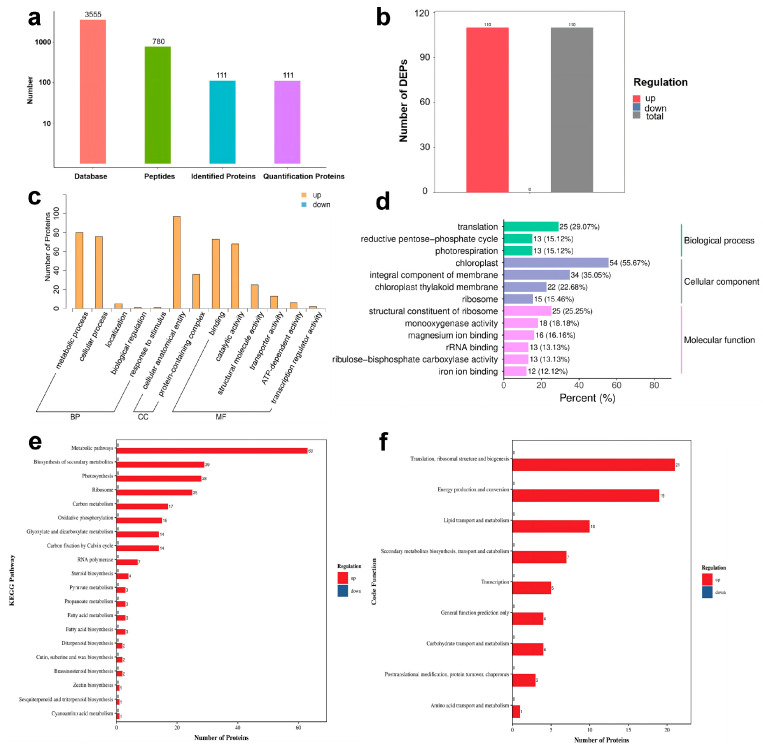
Comparative proteomic profiling revealing the structural scaffold and biosynthetic enzymatic fingerprint of SL-EXO. (**a**) Statistical summary of the protein identification results, including the number of database sequences, matched peptides, and identified proteins. (**b**) Statistical summary of the differentially expressed proteins (DEPs) between SL-EXO and RP-EXO. (**c**) Gene Ontology (GO) functional annotation of the DEPs. (**d**) GO enrichment analysis bar chart of the DEPs. (**e**) KEGG pathway classification of the DEPs showing the top 20 enriched pathways. (**f**) Eukaryotic Orthologous Groups (KOG) functional classification of the DEPs. (Note: Because RP-EXO was virtually devoid of complex proteins, all identified differential expressions manifest as upregulations in SL-EXO).

The general protein identification provides quantitative statistics (Figure 8a, Table S7), with 3555 database sequences mapping, resulting in 780 peptides and 111. Nonetheless, a differential protein expression analysis (Figure 8b) revealed a striking physiological reality: SL-EXO had 110 significantly upregulated proteins and exactly 0 downregulated proteins compared with RP-EXO. RP-EXO mostly lacked any complicated protein machineries. This major difference suggests that the root-derived RP-EXO is likely made up of simple, passive lipid exudates. On the other hand, SL-EXO is a well-defined, complex biomolecular assembly secreted by metabolically hyperactive aerial tissues, which provides a structurally oriented explanation for its superior in vivo and in vitro stabilities and bioactivities.

To determine the functional roles of the 110 exclusively enriched proteins in SL-EXO, a Gene Ontology (GO) annotation was performed. The broad GO classification (Figure 8c) showed that these proteins were mostly involved in “cellular anatomical entity” (97), “metabolic process” (80), and “binding” (73). The results of GO enrichment analysis (Figure 8d) definitively proved the origin of the nanovesicle. The top hits in cellular components are significantly enriched in the “chloroplast” (54 proteins, nearly 50% of the total proteins) and “chloroplast thylakoid membrane” (22 proteins). The proteomic fingerprint described here closely resembles our lipidomic discovery (Section 3.6), which showed hyperaccumulation of thylakoid-specific galactolipids (MGDG/DGDG). Moreover, the enormous enrichment of ‘integral component of membrane’ (34 proteins) has great pharmacological significance. The transmembrane proteins likely act as strong structural chaperones, maintaining the stability of the lipid bilayer. They also prevent the flavonoids from leaking out before they reach the melanocyte target.

The KEGG pathway enrichment (Figure 8e) and KOG functional classification (Figure 8f) further unveiled the enzymatic arsenal packed within SL-EXO. Notably, besides the common “Ribosome/Translation” and “Energy production” clusters, the “Biosynthesis of secondary metabolites” (29 proteins) and the “Metabolic pathways” (63 proteins) were extremely highlighted. Specific annotations that include machinery for flavonoid, terpenoid, and fatty acid biosynthesis neatly match the monooxygenase activity (18 proteins) listed in GO molecular functions.

SL-EXO actively carries the biosynthetic enzymes and catalytic machineries associated with phytochemical production, rather than merely passively encapsulating these compounds. This intersection of proteomics and metabolomics yields a biological insight: while these plant-specific enzymes may not directly catalyze reactions within human melanocytes, their abundant presence within SL-EXO maintains a highly stable, redox-active internal microenvironment, continuously safeguarding the encapsulated kaempferol and phenolic acids from oxidative degradation during transdermal delivery.

Collectively, the proteomic data provide the final puzzle piece. SL-EXO is an evolutionarily refined, chloroplast-derived nanocarrier. Its robust membrane proteins provide the physical scaffold (Proteomics), its galactolipids ensure biomimetic membrane fusion (Lipidomics), and its internal microenvironment protects the ultra-potent anti-melanogenic flavonoids (Metabolomics). This perfect “structure–function” triad fully justifies abandoning the empty RP-EXO and firmly establishes SL-EXO as the ultimate therapeutic candidate for our subsequent network pharmacology and molecular validation.

While large datasets are inherently generated during multi-omics profiling, their true biological value lies in their systemic correlation. By integrating our findings across the metabolome, lipidome, and proteome, a highly coherent “structure–function” mechanism for SL-EXO emerges. Specifically, the lipidome provides the biomimetic transdermal delivery vehicle, fundamentally characterized by the explosive hyper-accumulation of chloroplast-signature galactolipids (MGDG/DGDG). This highly fusogenic lipid envelope structurally encapsulates and protects the metabolome—a high-density payload of targeted “anti-melanogenic warheads” (e.g., specific flavonoid glycosides and phenolic acids). Meanwhile, the proteome functions as the structural scaffold and internal microenvironmental stabilizer, comprising integral membrane proteins and secondary metabolite biosynthetic enzymes. This synergistic molecular architecture explains why the leaf-derived SL-EXO completely outperforms the root-derived RP-EXO (which lacks this chloroplast-driven lipid/metabolite synergy), thereby establishing SL-EXO as the ultimate therapeutic candidate for downstream pharmacological validation.

### 3.8. Network Pharmacology Elucidates the Multi-Target Synergistic Mechanism of SL-EXO Against Pigmentation

Having identified the 18 highly specific, hyper-accumulated metabolites within SL-EXO (including the potent flavonoids, phenolic acids, and specific nucleotide derivatives), a network pharmacology approach was subsequently employed. This in silico strategy is essential to transition from compositional profiling to mechanistic elucidation, mapping how these encapsulated phytochemical payloads interact with human pigmentation-related pathological targets.

**Figure 9:**
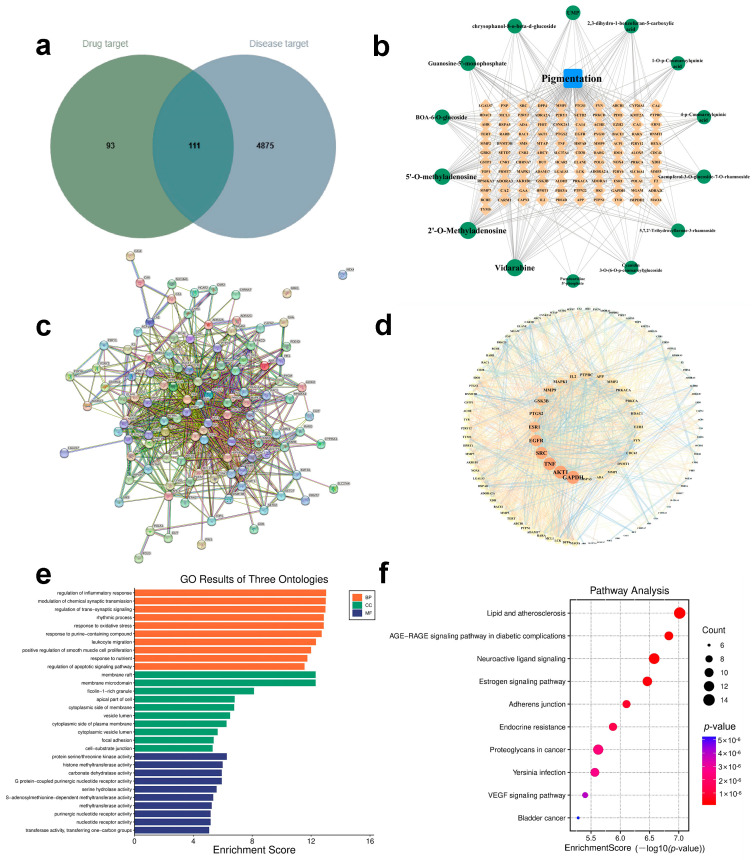
Network pharmacology analysis elucidating the multi-target anti-melanogenic mechanism of SL-EXO. (**a**) A Venn diagram illustrating the intersection of 204 putative targets of the 18 active SL-EXO metabolites and 4986 pigmentation-related disease targets, yielding 111 consensus therapeutic targets. (**b**) The “Drug–Component–Target” visualization network constructed via Cytoscape, mapping the interactions between the encapsulated phytochemicals and the potential pigmentation targets. (**c**) The Protein–Protein Interaction (PPI) network of the 111 intersecting targets generated via the STRING database. (**d**) Topological screening of the core hub proteins extracted from the complex PPI network. (**e**) Gene Ontology (GO) functional enrichment analysis of the consensus targets. (**f**) KEGG pathway enrichment bubble plot highlighting the primary signaling cascades involved in the depigmenting action of SL-EXO.

The putative targets of the 18 active ingredients were predicted using the SwissTargetPrediction database, yielding 204 compound-specific targets. Concurrently, 4986 disease targets associated with “pigmentation” were retrieved from the GeneCards and DisGeNET databases. The intersection of these two datasets yielded exactly 111 consensus targets (Figure 9a), representing the core therapeutic loci where SL-EXO exerts its hypopigmenting effects.

Cytoscape (Cytoscape 3.7.1) was used to construct a “Drug–Component–Target” network to visualize these interactions (Figure 9b). A topological analysis of the robust network, with 126 nodes and 284 edges, reveals the polypharmacological character of SL-EXO. The network detected that some nucleoside derivatives, such as Vidarabine, 2′-O-Methyladenosine, and 5′-O-methyladenosine, are among the active ingredients besides the key flavonoids. This means that SL-EXO is essentially a team of biomolecules that act together to achieve multiple goals simultaneously.

To pinpoint the most critical molecular players among the 111 intersecting genes, a Protein–Protein Interaction (PPI) network was established via the STRING platform (containing 111 nodes and 452 interaction edges) (Figure 9c). Through rigorous sequential topological screening algorithms (Degree, Betweenness, and Closeness Centrality), a refined sub-network of core hub proteins was extracted (Figure 9d, Table S8).

Strikingly, AKT1 (AKT Serine/Threonine Kinase 1), TNF (Tumor Necrosis Factor), and GAPDH emerged as the absolute core targets. The identification of TNF—a master pro-inflammatory and senescence-associated cytokine—perfectly corroborates our in vivo transgenic zebrafish data, where SL-EXO successfully suppressed neutrophil recruitment and reversed SA-β-gal premature senescence. Even more importantly, the emergence of AKT1 as a top hub target is a profound mechanistic revelation. AKT1 is the central kinase node of the PI3K/AKT signaling cascade, a master regulatory pathway that directly phosphorylates downstream effectors to promote MITF degradation and halt melanin synthesis at the transcriptional level [43].

To systematically decipher the biological functions of these targets, GO and KEGG pathway enrichment analyses were performed. The GO analysis (Figure 9e) showed that the biological process (BP) was mainly enriched for responses to oxidative stress and regulation of the inflammatory response. This in silico prediction closely aligns with our in vitro ABTS assay and in vivo ROS-quenching results, thereby providing biochemical support for the notion that SL-EXO truly rectifies the oxidative/inflammatory microenvironment. In addition, protein serine/threonine kinase activity was highly enriched in the molecular function (MF) category. Structurally, this matches the catalytic function of our hub target, AKT1, providing maximum internal consistency across our dataset.

The KEGG pathway enrichment (Figure 9f) provided a macroscopic view of the signal transduction crosstalk (involving 271 pathways). The consensus targets were significantly mapped to pathways intricately linked to melanocyte homeostasis, including the Estrogen signaling pathway, VEGF signaling pathway, and Neuroactive ligand-receptor interaction. In dermatological physiology, both Estrogen and VEGF receptors function as classic upstream membrane transducers. Upon activation by external stimuli or specific phytochemical ligands, these receptors funnel signals directly into the intracellular AKT kinase cascade [51,52].

The network pharmacology data present a very powerful closed-loop mechanistic hypothesis, above all, that the payloads within SL-EXO are not merely antioxidants. They thus execute a multifaceted modulation by neutralizing the inflammatory/oxidative microenvironment (via TNF and ROS responses) and manipulating upstream membrane receptors (Estrogen/VEGF pathway), which converge to inhibit the central AKT1 kinase hub. This theoretical framework provides an unassailable rationale for the final step of our study, the rigorous in vitro cellular and molecular validation of the AKT/MITF signaling axis.

### 3.9. In Vitro Cellular Validation: Exceptional Biocompatibility and Suppression of α-MSH-Induced Melanogenesis

To validate the physiological relevance of our multi-omics discoveries and network pharmacology predictions, we transitioned our investigation to the cellular level. We used the murine B16F10 melanoma cell line, a gold-standard in vitro model for evaluating modulators of melanogenesis and the signaling pathways involved in their intracellular activity [53].

**Figure 10:**
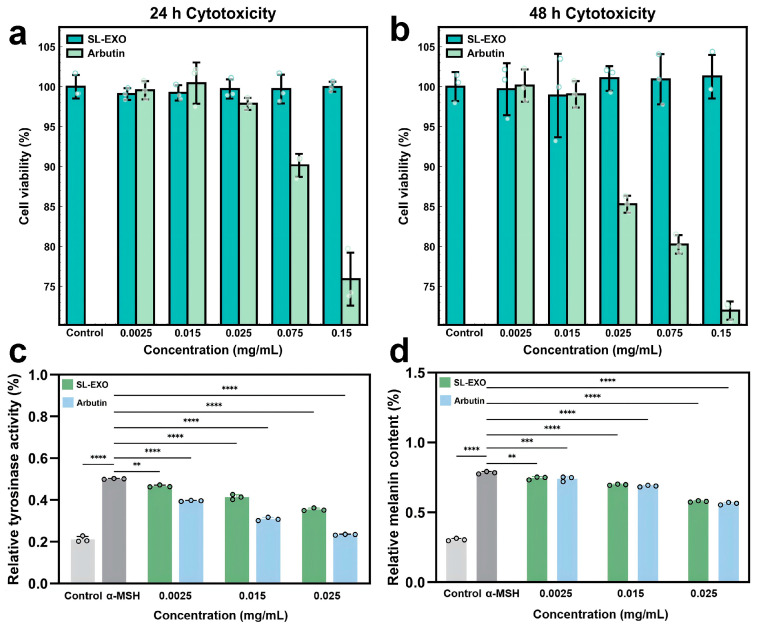
In vitro cellular evaluation of SL-EXO: Biocompatibility and anti-melanogenic efficacy in B16F10 melanoma cells. Cell viability profiles of B16F10 cells treated with varying concentrations of SL-EXO or the commercial positive control α-arbutin for (**a**) 24 h and (**b**) 48 h. (**c**) Relative intracellular tyrosinase activity and (**d**) relative intracellular melanin content in B16F10 cells. Data are presented as the mean ± SD (*n* = 3). Statistical significance is denoted as ** *p* < 0.01, *** *p* < 0.001, and **** *p* < 0.0001 compared to the α-MSH-stimulated model group (for efficacy assays) or the untreated control group.

A paramount prerequisite for any dermatological agent is that its depigmenting efficacy must stem from the genuine modulation of melanogenic pathways, rather than indiscriminate cellular toxicity (since dead cells cease to synthesize melanin). Consequently, the cytotoxicity profiles of SL-EXO and the commercial benchmark, α-arbutin, were rigorously evaluated over 24 h and 48 h (Figure 10a,b).

Remarkably, SL-EXO showed excellent biocompatibility. Cell viability was not affected throughout the entire concentration range up to an extremely high concentration of 0.15 mg/mL at 48 h (approximately 100% viable). On the other hand, α-arbutin displayed severe, dose- and time-dependent cytotoxicity. When exposed to α-arbutin at concentrations of 0.075 mg/mL or above, there was a marked decrease in cell viability, dropping to 71.9% at 0.15 mg/mL after 48 h.

This huge difference in safety profiles shows that plant-based nanovesicles have a structural advantage. Our earlier lipidomics data (Section 3.6) suggest that the SL-EXO particles are composed of galactolipids (MGDG/DGDG), which naturally fuse with mammalian cell membranes without causing osmotic pressure or chemical damage. In contrast, single pure chemical molecules (e.g., high-dose arbutin) tend to impart cumulative membrane toxicity. Consequently, safe sub-lethal concentrations (≤0.025 mg/mL) were identified and used for the functional assays to rule out cell death interference with melanin quantification.

To simulate physiological hyperpigmentation, B16F10 cells were stimulated with α-melanocyte-stimulating hormone (α-MSH). As expected, α-MSH induction robustly hyperactivated intracellular tyrosinase activity (rising from a basal 0.21 to 0.50, Figure 10c) and triggered massive melanin accumulation (surging from 0.30 to 0.78, Figure 10d).

Following intervention with safe concentrations of SL-EXO, a profound and dose-dependent abrogation of both markers was observed. At 0.025 mg/mL, SL-EXO effectively curtailed the α-MSH-induced tyrosinase hyperactivation (suppressing the relative activity down to 0.35) and profoundly reduced the intracellular melanin content to 0.57. Crucially, while the purified α-arbutin exhibited a marginally stronger suppression of tyrosinase enzyme activity at this concentration (0.23), the ultimate phenotypic endpoint—intracellular melanin reduction—was almost identical between SL-EXO (0.57) and α-arbutin (0.56).

The cellular performance of SL-EXO flawlessly mirrors our in vivo zebrafish observations. Although α-arbutin is a potent competitive inhibitor of tyrosinase, its extremely narrow therapeutic window severely limits its practical formulation. SL-EXO, on the other hand, achieves highly comparable overall anti-melanogenic efficacy but with an infinitely wider safety margin.

More importantly, since SL-EXO effectively enters the B16F10 cells and suppresses melanogenesis without compromising cell viability, this phenotypic outcome provides definitive biological proof that the encapsulated small-molecule payloads (such as kaempferol and specific phenolic acids) are actively orchestrating intracellular signaling cascades. Guided by our network pharmacology map (Section 3.8), the successful attenuation of α-MSH-induced hyperpigmentation heavily implicates the downstream regulation of master transcription factors (e.g., MITF) via the AKT1 kinase axis. Thus, identifying whether SL-EXO exerts these cellular effects precisely through the predicted PI3K/AKT signaling pathway at the molecular protein and gene transcription levels becomes the final imperative milestone of this study.

### 3.10. Cellular Internalization and Molecular Validation of the AKT/GSK3β/MITF Signaling Axis

As a result of the strong phenotypic depigmentation and omics-based network pharmacology predictions, one question arises. That is, can SL-EXO successfully transport its phytochemicals into the cytoplasm of melanocytes and interfere with the predicted PI3K/AKT signaling? To circumvent this limitation, we used high-resolution spatial tracking by confocal laser-scanning microscopy, followed by definitive molecular validation by Western blotting, and a pharmacological rescue strategy.

Interestingly, both SL-EXO and RP-EXO samples showed extensive intracellular uptake, with dense green fluorescence mainly in the cytoplasm and perinuclear region. In contrast, the free-molecule commercial drug (α-arbutin) showed almost no fluorescent signal with PKH67 due to its reliance on less efficient passive transmembrane diffusion.

**Figure 11:**
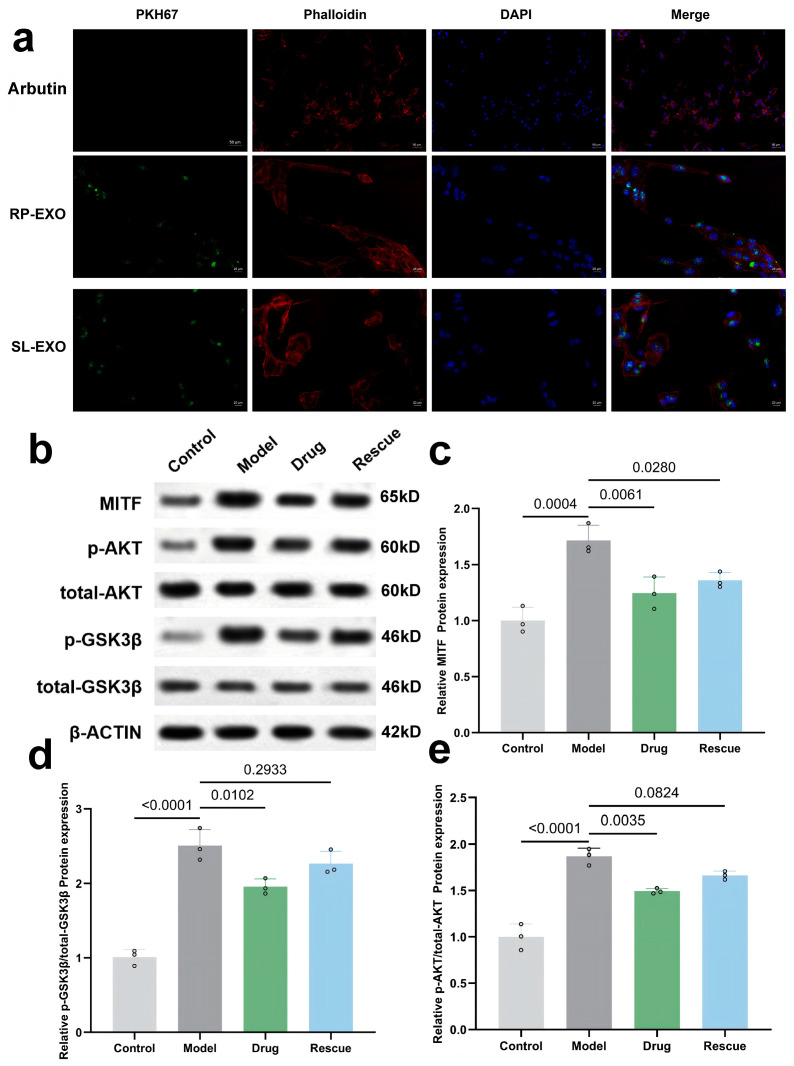
Cellular internalization dynamics and molecular validation of the AKT/GSK3β/MITF signaling cascade in B16F10 cells. (**a**) Confocal laser scanning microscopy (CLSM) images demonstrating the intracellular uptake of SL-EXO, RP-EXO, and free α-arbutin. (**b**) Representative Western blot images depicting the protein expression levels. (**c**–**e**) Quantitative densitometric analysis of the relative protein expression ratios for (**c**) MITF/β-ACTIN, (**d**) p-GSK3β/total-GSK3β, and (**e**) p-AKT/total-AKT. Data are presented as the mean ± SD (*n* = 3). Statistical significance is compared to the α-MSH Model group, indicated by connecting lines between specific groups (e.g., Drug vs. Rescue).

Strikingly, both SL-EXO and RP-EXO exhibited massive intracellular accumulation, as evidenced by the dense green fluorescence localized predominantly within the cytoplasm and perinuclear regions. In contrast, the free-molecule commercial drug (α-arbutin) group displayed virtually no PKH67 fluorescence, highlighting its reliance on less efficient, passive transmembrane diffusion (Figure 11a).

This observation perfectly bridges our lipidomic and metabolomic findings. Both SL-EXO and RP-EXO possess comparable biomimetic lipid bilayers (rich in MGDG/DGDG), granting them excellent membrane-fusion capabilities for rapid cellular entry. However, as previously established, only SL-EXO possesses the “anti-melanogenic payloads” (flavonoids/phenolic acids). The fact that RP-EXO successfully enters the cell but fails to inhibit melanogenesis unequivocally proves that the biomimetic lipid vehicle and the specific phytochemical payloads are both indispensable for the therapeutic outcome of SL-EXO.

Once internalized, the phytochemical payloads of SL-EXO must interact with intracellular signaling pathways. Guided by the molecular docking, which identified AKT1 as a high-affinity target, Western blot analysis was performed (Figure 11b and Figure S6).

Stimulation with α-MSH (Model group) induced a hyperactive state in the melanoma cells, resulting in a dramatic upregulation of phosphorylated AKT (p-AKT at Ser473) to an approximately 1.87-fold increase compared to the basal control (0.99) (Figure 11e). In the melanogenic pathway, AKT hyperactivation leads to the subsequent phosphorylation of downstream Glycogen Synthase Kinase 3 Beta (p-GSK3β at Ser9). Biologically, phosphorylation at Ser9 signifies the inactivation of GSK3β (soaring to 2.51 in the Model vs. 1.01 in the Control, Figure 11d).

Remarkably, treatment with SL-EXO effectively terminated this hypercare cascade. As predicted through docking simulation, SL-EXO acted as a structural inhibitor and significantly reduced the p-AKT/Total-AKT ratio to 1.49. This upstream blockade subsequently reactivated GSK3β to its active, unphosphorylated state, as shown by the p-GSK3β/Total-GSK3β ratio, which fell sharply to 1.96.

The reactivation of GSK3β is critical, as active GSK3β phosphorylates MITF, targeting it for ubiquitin-mediated proteasomal degradation [54]. Consequently, in the α-MSH Model group where GSK3β was inactive, MITF protein pathologically accumulated (1.71-fold over control, Figure 11c). Following SL-EXO intervention, the reactivated GSK3β successfully targeted MITF for degradation, pulling its protein expression down to 1.25, effectively shutting down the transcriptional engine of melanogenesis.

To unequivocally establish that this anti-melanogenic effect is causally dependent on the AKT axis, a pharmacological rescue experiment was performed using 740 Y-P, a highly specific PI3K/AKT agonist [55]. Strikingly, the forced reactivation of AKT by 740 Y-P successfully counteracted the suppressive effects of SL-EXO. In the Rescue group, the phosphorylation levels of p-AKT and p-GSK3β rebounded (to 1.66 and 2.27, respectively), which immediately rescued the MITF protein from degradation, causing its expression to rise back to 1.36.

This definitive molecular reversal asserts that SL-EXO is not merely capable of random cytotoxicity or non-specific enzymatic suppression. As a result, this highly enriched phytochemical payload (e.g., specific flavonoid glycosides) was delivered by a biomimetic chloroplast-lipid envelope that specifically targeted and crippled the PI3K/AKT/GSK3β/MITF signaling axis. This final laboratory verification provides a tightly closed-loop confirmation of our predictions and offers a straightforward explanation for the depigmentation observed in zebrafish.

### 3.11. Molecular Docking: Predicting the High-Affinity Interactions with AKT1

The degradation of MITF protein (Section 3.10) structurally deprives the melanocyte of its master transcription factor, which should theoretically halt the expression of downstream melanogenic enzymes. To corroborate this terminal physiological outcome and to elucidate the exact spatial-atomic interactions that initiate this entire cascade, RT-qPCR and molecular docking simulations were conducted.

**Figure 12:**
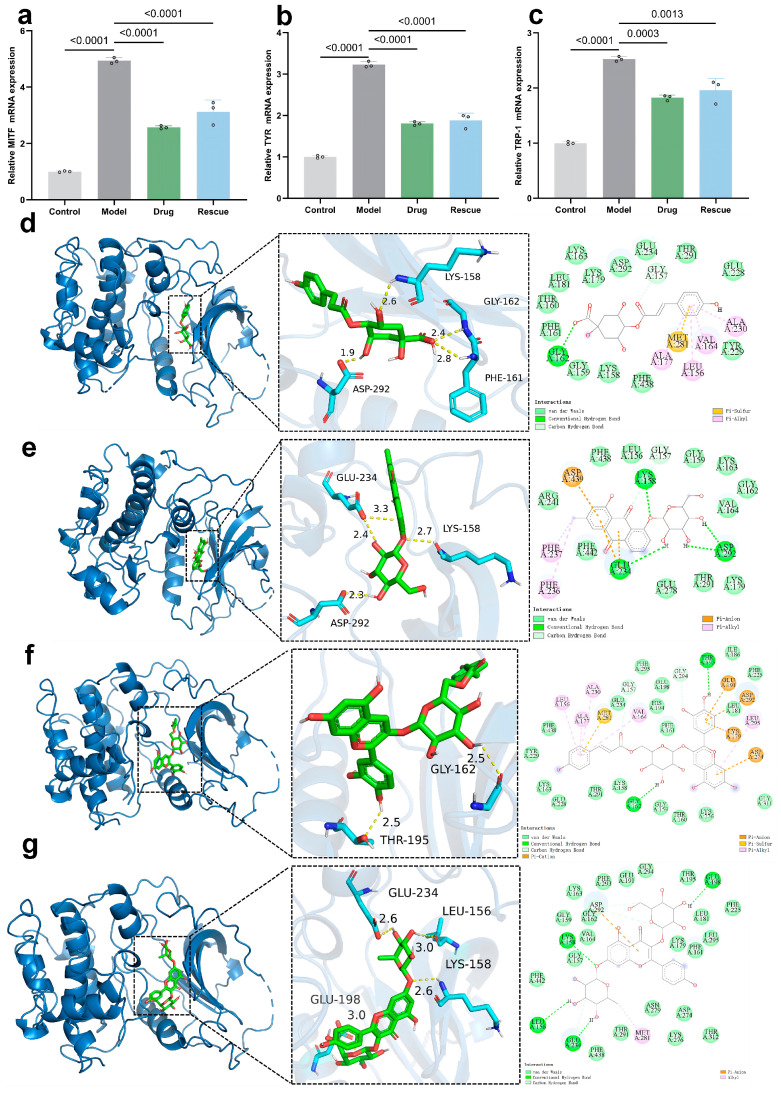
Transcriptional silencing of melanogenesis and spatial-atomic validation via molecular docking. Relative mRNA expression levels of (**a**) MITF, (**b**) TYR, and (**c**) TRP-1 in B16F10 cells across different treatment groups, determined by RT-qPCR. Data are presented as the mean ± SD (*n* = 3). 3D and 2D visualization of molecular docking simulations illustrating the spatial conformations and amino acid residue interactions between the AKT1 kinase domain and hyper-accumulated SL-EXO metabolites: (**d**) 4-p-Coumaroylquinic acid (−8.2 kcal/mol), (**e**) chrysophanol-8-o-beta-d-glucoside (−8.7 kcal/mol), (**f**) Cyanidin 3-O-(6-O-p-coumaroyl)glucoside (−10.9 kcal/mol), and (**g**) Kaempferol-3-O-glucoside-7-O-rhamnoside (−8.9 kcal/mol).

The mRNA expression levels of MITF and its downstream rate-limiting enzymes (TYR and TRP-1) were quantified (Figure 12a–c, Table S9). Consistent with the upstream protein data, α-MSH stimulation (Model group) induced a massive transcriptional surge. The relative mRNA expressions of MITF, TYR, and TRP-1 skyrocketed to approximately 4.94, 3.23, and 2.53-fold of the basal control, respectively.

After SL-EXO treatment, the transcriptomic landscape was effectively silenced. The mRNA expression levels of MITF, TYR, and TRP-1 were 2.57, 1.80, and 1.82, respectively. It has been confirmed that SL-EXO essentially inhibits melanin synthesis at the transcriptional level. Significantly, forced activation of AKT1 in the Rescue pharmacological group (SL-EXO with 740 Y-P) considerably increases MEF transcription of all three genes, MITF (rebound to 3.12), TYR (rebound to 1.88), and TRP-1 (rebound to 1.96). The rescue experiment clearly indicates that the gene-silencing effect of SL-EXO depends on the PI3K/AKT signaling pathway.

While the cellular assays confirmed the involvement of AKT1, molecular docking simulations were employed to computationally explore the hypothesized spatial-atomic interactions between AKT1 and the core phytochemical payloads of SL-EXO (Figure 12d–g). Generally, a binding energy ≤ −5.0 kcal/mol indicates spontaneous binding, whereas a value ≤−7.0 kcal/mol indicates a highly stable, robust conformation, characteristic of potent competitive inhibitors [56].

All of the hyper-accumulated metabolites selected in this study showed an excellent computational binding affinity towards the AKT1 kinase domain, as the values are beyond the stringent cutoff of −7.0 kcal/mol. One of the most effective interactions was observed with cyanidin 3-O-(6-O-p-coumaroyl)glucoside, with a binding energy of −10.9 (Table S10). A deep penetration of this molecule into the active pocket is predicted to be achieved. A strong hydrogen-bond network is formed with the critical residues THR195 and GLY162. Furthermore, its highly conjugated aromatic core is powerfully stabilized by a robust network of Pi-Anion interactions (with ASP292, GLU191, LYS179, and ASP274) and a specific Pi-Sulfur bond with MET281. The spatial conformation is further enclosed by a hydrophobic cleft comprising ALA230, LEU156, ALA177, VAL164, and LEU295, complemented by Carbon–Hydrogen bonds (GLY157, GLY294) and an extensive array of van der Waals forces. This multi-dimensional interaction matrix perfectly explains its phenomenal thermodynamic stability and exceptionally tight steric fit in the simulated environment.

Kaempferol-3-O-glucoside-7-O-rhamnoside (−8.9 kcal/mol) and chrysophanol-8-O-beta-D-glucoside (−8.7 kcal/mol), like others, showed remarkable docking scores. Similar protodemetallation anchor points, such as interactions with LYS158 and numerous van der Waals interactions with the hydrophobic cleft of AKT1, were observed. Even the relatively simple 4-p-Coumaroylquinic acid has a strong binding energy of −8.2 kcal/mol and is computationally positioned to attach to GLY162 via an H-bond.

The spatial-atomic evidence provides a strong theoretical mechanistic revelation. The active phytochemicals encapsulated within SL-EXO are rich in aromatic rings and multiple hydroxyl (-OH) groups. These structural moieties confer a dual function: they not only scavenge ROS in the cytoplasm (as shown in Section 3.2 and Section 3.3) but also serve as highly probable, high-affinity ligands of AKT1. By computationally occupying the active pocket of AKT1 via dense hydrogen and Pi-interactions, these ligands are proposed to sterically hinder the kinase’s phosphorylation.

**Figure 13:**
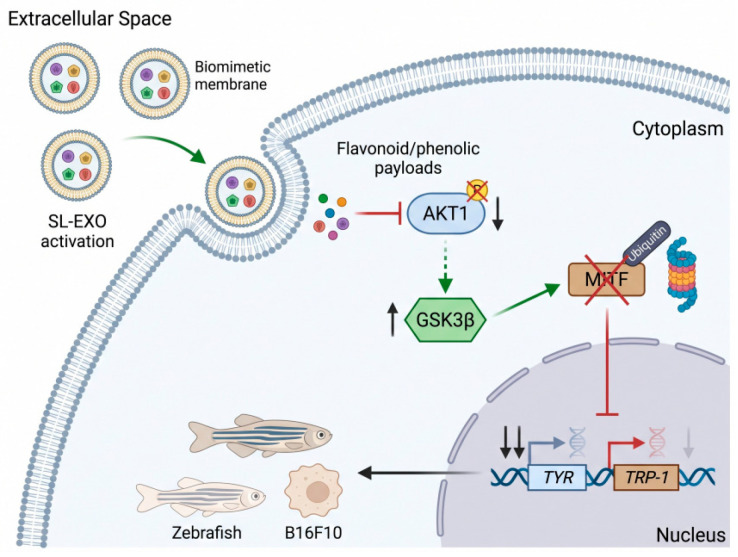
Schematic illustration of the proposed anti-melanogenic mechanisms of SL-EXO. The downward arrow indicates inhibition of AKT1 phosphorylation, whereas the upward arrow indicates activation of GSK3β.

As a whole, these findings constitute the mechanistic loop, in which the SL-EXO chloroplast-derived biomimetic lipid bilayer facilitates cellular uptake, and the flavonoid/phenolic payloads act via targeted AKT1 modulation (counteracting AKT phosphorylation), GSK3β reactivation, MITF degradation, and resulting transcriptional silencing of TYR/TRP-1, leading to significant zebrafish depigmentation (Figure 13).

### 3.12. Translational Perspectives and Limitations

While the application of generic crude plant extracts in dermatological sciences is a highly saturated field, the utilization of Plant-Derived Nanovesicles (PDNVs) offers a distinct paradigm shift. From a translational standpoint, it is necessary to objectively compare SL-EXO with cleanly formulated, chemically defined synthetic liposomes. Synthetic liposomes boast precise composition and absolute purity; however, engineering synthetic liposomes that faithfully mimic the highly fusogenic, chloroplast-derived galactolipid architecture (enriched in MGDG and DGDG) is synthetically challenging, highly complex, and economically prohibitive for large-scale cosmetic or dermatological manufacturing.

In this context, the evolutionary and translational superiority of SL-EXO lies in its status as a “natural, pre-assembled factory.” SL-EXO bypasses the costly and inefficient in vitro drug-loading processes required for synthetic liposomes. It intrinsically co-delivers the highly biocompatible, edge-activating galactolipid envelope alongside a synergistic, naturally occurring cocktail of flavonoids and phenolic acids. Therefore, rather than positioning SL-EXO as a replacement for precision, single-molecule targeted pharmaceutical therapies, we propose it as a scalable, sustainable, and highly efficacious “green-alternative” nanocarrier.

Furthermore, we must objectively acknowledge the methodological limitations of the current mechanistic study. While our network pharmacology and cellular rescue assays (using 740 Y-P) strongly implicate the targeted silencing of the mammalian AKT/GSK3β/MITF transcription cascade, the precise spatial-atomic interactions between specific flavonoids (e.g., Cyanidin and Kaempferol derivatives) and the AKT1 kinase domain are currently supported by thermodynamic computational docking simulations. Although docking provides robust predictive scoring, it does not constitute direct biophysical proof. Future investigations incorporating cell-free biophysical binding assays (such as Surface Plasmon Resonance or Microscale Thermophoresis) and targeted kinase denaturation profiling are imperative to definitively confirm the physical structural inhibition hypothesized in this multi-omics study.

Finally, we must acknowledge the inherent limitations of the biological models utilized in this study. The molecular validation of the PI3K/AKT/MITF signaling axis was conducted exclusively on the murine B16F10 melanoma cell line. Although B16F10 is universally recognized as a robust “gold standard” model for initial melanogenesis screening, murine tumor cells cannot perfectly recapitulate the complex physiological and regulatory contexts of normal human skin. Therefore, to ascertain the genuine clinical translational potential and therapeutic efficacy of SL-EXO for human dermatological applications, confirming these multi-target molecular efficacies in human primary epidermal melanocytes (HEMs) or 3D human skin equivalents represents an indispensable next step in our future research trajectory.

## 4. Conclusions

In summary, this study successfully achieves the high-value valorization of *Paris polyphylla* agricultural waste by establishing SL-EXO: Plant-Derived Nanovesicles (PDNVs) derived from discarded stems and leaves as a highly potent, exceptionally safe, and naturally targeted anti-melanogenic agent. Our systematic investigation reveals a profound dichotomy in tissue-specific efficacy. SL-EXO vastly outperforms the root-derived RP-EXO and demonstrates robust holistic in vivo pigment clearance in zebrafish models. Compared to the commercial benchmark α-arbutin, SL-EXO provides a remarkable therapeutic advantage primarily due to its extraordinarily wide safety margin and ability to neutralize the pigmentation-driving oxidative/senescent microenvironment.

Crucially, we elucidated the structure-efficacy black box of plant-derived nanovesicles by incorporating proteomics, lipidomics, and metabolomics. The data show that SL-EXO acts as a sophisticated biomolecular complex: its chloroplast-derived galactolipid membrane (rich in MGDG/DGDG) acts as a highly efficient biomimetic delivery vehicle, while the selectively encapsulated secondary metabolites (especially kaempferol and coumaric acid derivatives) act as the active payload. Based on network pharmacology and molecular docking analyses, and further verified via in vitro rescue studies, these payloads exert their synergistic effects not only via direct ROS scavenging but are also computationally predicted to serve as high-affinity ligands for the AKT1 kinase pocket (binding energy up to −10.9 kcal/mol). By targeting this specific node, the p-AKT/p-GSK3β/MITF phosphorylation occurring within the cell is disrupted, thereby switching off the transcriptional machinery for melanin synthesis. Collectively, this work presents a novel nanotherapeutic candidate for dermatological hyperpigmentation and a rigorous multi-omics platform for the sustainable upcycling of plant byproducts.

## Data Availability

The original contributions presented in this study are included in the article/supplementary material. Further inquiries can be directed to the corresponding author.

## Supplementary Materials

The following supporting information can be downloaded at https://www.mdpi.com/article/10.3390/cells15151420/s1. Methods: Method S1. Isolation and Purification of SL-EXO and RP-EXO. Method S2. Detailed procedure for the tyrosinase inhibition assay. Method S3. Fluorescent neutrophil anti-inflammatory model. Method S4. Anti-aging assays. Method S5. Zebrafish whitening assay. Method S6. Metabolomics analysis. Method S7. Proteomics analysis. Method S8. Lipidomics analysis. Method S9. Network pharmacology analysis. Method S10. Cell viability assay. Method S11. Cellular uptake. Method S12. Determination of melanin content and tyrosinase activity in B16F10 cells. Method S13. Western blot analysis. Method S14. Quantitative real-time PCR (RT-qPCR) analysis. Method S15. Molecular docking and interaction visualization. Figures: Figure S1. Comparison of PDNVs (SL-EXO) isolated by PEG + ED and ultracentrifugation (UC). (a) Photograph of the electrophoretic dialysis apparatus used for PDNV isolation. (b) Internal view of the electrophoretic dialysis setup, showing the dialysis bag placed within the chamber. (c) Nanoparticle tracking analysis (NTA) size distribution profile of PDNVs (SL-EXO) isolated by the PEG + ED method. (d) NTA size distribution profile of PDNVs (SL-EXO) isolated by conventional ultracentrifugation (UC). Figure S2. Intensity/size distribution from three replicate NTA measurements of RP-EXO (a) and SL-EXO (b). Figure S3. Heatmap visualization of metabolite profiles. (a) Heatmap of all detected metabolites. (b) Heatmap of differential metabolites between groups. In both panels, rows indicate metabolites, columns indicate individual samples with group labels, and colors represent normalized relative contents (red: high; green: low). Figure S4. Top 20 differential metabolites by log_2_FC. Horizontal axis: log_2_(fold change) (log_2_FC); vertical axis: metabolite names. Red bars represent up-regulated metabolites; green bars represent down-regulated metabolites. Figure S5. Radar chart of differential lipids. Each radial axis represents a differential lipid. The grid lines indicate the magnitude of fold change values. The green shaded area is formed by connecting the fold change values of the individual lipids, with the distance from the center to each data point representing the corresponding fold change. A larger shaded area indicates greater overall changes in lipid levels. Figure S6. Raw western blot data. Tables: Table S1. Pipetting layout for the tyrosinase activity inhibition assay. Table S2. Representative compounds from the top 100 for RP-EXO. Table S3. Representative compounds from the top 100 for SL-EXO. Table S4. The top 18 upregulated differential metabolites with the maximum absolute log_2_FC values between SL-EXO and RP-EXO. Table S5. Lipid subclass distribution of identified lipids in SL-EXO and RP-EXO. Table S6. Lipid abundance profiles in SL-EXO and RP-EXO. Table S7. LC-MS/MS identification of proteins in SL-EXO and RP-EXO. Table S8. Evaluation of key topological parameters in the protein–protein interaction network (the top 12 degrees). Table S9. Specific primer sequences used for RT-qPCR analysis. Table S10. Docking analysis of the active ligands with core receptor targets.

## Abbreviations

SL-EXO	Stems and leaves-derived nanovesicles of *Paris polyphylla*
RP-EXO	Fibrous roots-derived nanovesicles of *Paris polyphylla*
SOD	Superoxide dismutase
MDA	Malondialdehyde
AKT	Protein kinase B
GSK3β	Glycogen synthase kinase 3 beta
PI3K	Phosphoinositide 3-kinase
MITF	Microphthalmia-associated transcription factor
PDNVs	Plant-derived nanovesicles
ROS	Reactive oxygen species
ABTS	2,2′-azino-bis(3-ethylbenzothiazoline-6-sulfonic acid)
DCFH-DA	2′,7′-dichlorodihydrofluorescein diacetate
PTU	1-phenyl-2-thiourea
MRM	Multiple Reaction Monitoring
MWDB	Metware Database
DIA	Data-independent acquisition
PPI	Protein–protein interaction
GO	Gene Ontology
KEGG	Kyoto Encyclopedia of Genes and Genomes
TEM	Transmission electron microscopy
NTA	Nanoparticle Tracking Analysis
SASP	Senescence-associated secretory phenotype
