# Supplementary material for: Valorization of Paris polyphylla Byproducts: Integrated Multi-Omics and Molecular Docking Reveal the Anti-Melanogenic Mechanism of Plant-Derived Nanovesicles via the AKT/GSK3β/MITF Axis
Source: Cells. 2026 Aug 5;15(15):1420. doi: 10.3390/cells15151420 (PMC13465535; doi:10.3390/cells15151420)
Supplement: Supplementary file 1 [file cells-15-01420-s001.zip › cells-4444684-supplementary.pdf]

Supplementary Information

Valorization of *Paris polyphylla* Byproducts: Integrated Multi-Omics and Molecular Docking Reveal the Anti-Melanogenic Mechanism of Plant-Derived Nanovesicles via the AKT/GSK3β/MITF Axis

Peishi Feng [1,2,3], Li Tao [1,2,3] , Xiaoli Chen [1,2], Han Yang [1,2,3], Yida Zhang [1,2] and Ping Wang [1,2,3,*]

[1] School of Pharmacy, Zhejiang University of Technology, Hangzhou 310014, China
[2] Zhejiang Provincial Key Laboratory of TCM for Innovative R & D and Digital Intelligent Manufacturing of TCM Great Health Products, Hangzhou 310014, China
[3] Zhejiang International Scientific & Technological Cooperation Base of Development and Utilization of Natural Products, Zhejiang University of Technology, Hangzhou 310014, China.
* Correspondence: wangping45@zjut.edu.cn; Tel.: +86-13634108877

** Corresponding author Ping Wang, E-mail address: wangping45@zjut.edu.cn*

Materials and methods

Method S1:Isolation and Purification of SL-EXO and RP-EXO

SL-EXO and RP-EXO were isolated and purified according to our previously established method. Briefly, the fresh plant tissues (stems/leaves and fibrous roots) were homogenized (BARBOSA, Germany) and filtered through a 200-mesh sieve. The filtrate was subjected to two consecutive centrifugation steps at 10,000 × g for 30 min each, and the pellets were discarded to remove large fibers and cellular debris. Subsequently, PEG-6000 was added to the supernatant to a final concentration of 10% (w/v), and the mixture was incubated overnight at 4 °C. The crude vesicles were precipitated by centrifugation at 10,000 × g for 30 min, resuspended in PBS, and centrifuged again at 10,000 × g for 20 min to collect the vesicle-containing supernatant. This supernatant was concentrated to 5-10 mL using a 100 kDa centrifugal ultrafiltration unit and then subjected to electrodialysis (300 kDa MWCO, 300 mA) for further purification. Following electrodialysis, the samples were re-concentrated using the 100 kDa ultrafiltration unit. Finally, the protein concentrations of the purified SL-EXO and RP-EXO were determined using a BCA assay, and the aliquots were stored at -80 °C for subsequent experiments.

Method S2. Detailed procedure for the tyrosinase inhibition assay

The positive control α-arbutin, and tne SL-EXO and RP-EXO were each diluted in PBS to obtain a series of concentrations: 0.06, 0.08, 0.12, 0.15, 0.2, and 0.4 mg/mL. In a 96-well microplate, four groups were set: solvent blank (T_a), solvent reaction (T_b), sample blank (T_c), and sample reaction (T_d). The T_a group served as the solvent blank, to which neither L-tyrosine solution nor sample solution was added. The T_b group was the solvent reaction group, receiving L-tyrosine solution but no sample solution. The T_c group was the sample blank, receiving sample solution but no L-tyrosine solution. The T_d group was the sample reaction group, to which both L-tyrosine solution and sample solution were added. Three replicate wells were prepared for each group.

According to the reagent volumes listed in Table S1, L-tyrosine solution, sample solution (or solvent for the corresponding controls), and PBS buffer were added sequentially to each well and mixed thoroughly. After incubation at 37 °C for 10 min, 20 µL of tyrosinase solution was added to each well. The mixture was incubated at 37 °C for 5 min ± 5 s, after which the plate was immediately placed in a microplate reader, and absorbance was measured at 475 nm.

Note: The time interval from the addition of tyrosinase solution to the absorbance measurement was kept strictly consistent for all wells (5 min ± 5 s).

The tyrosinase activity inhibition rate was calculated according to the following equation:

$$\text{Inhibition rate (\%)} = (1\text{-}A_d\text{-}A_c\ /A_b\ \text{-}A_a)\times 100\%$$

Where A_a and A_b represent the mean absorbances of the solvent blank wells (T_a) and solvent reaction wells (T_b), respectively, and A_c and A_d are the absorbances of the sample blank well (T_c) and sample reaction well (T_d), respectively.

Method S3. Fluorescent neutrophil anti-inflammatory model

Transgenic neutrophil fluorescent zebrafish embryos Tg(lyz:eGFP) were cultured in E3 medium at 28.5 °C. At 12 hpf, unfertilized and malformed embryos were removed, and the remaining healthy embryos were divided into 10 groups. For each group, 10 embryos were transferred into one well of a 6-well plate, with three replicate wells per group, for a total of 30 wells. Each well received 5 mL of the corresponding treatment solution. The 10 experimental groups were: SL-EXO at 0.0001, 0.0005, and 0.001 mg/mL; RP-EXO at 0.0001, 0.0005, and 0.001 mg/mL; the positive control ibuprofen at the same three concentrations; and a blank control without any drug. All concentrations of SL-EXO and RP-EXO are expressed as protein concentration. To induce an inflammatory phenotype, lipopolysaccharide (LPS) was added to every well at a final concentration of 0.5 μg/mL. $CuSO_4$ was not used as an inducer because LPS specifically activates macrophages and neutrophils via the TLR4 pathway, eliciting an inflammatory response that closely mimics human bacterial infections, whereas $CuSO_4$ causes heavy-metal toxicity with non-specific tissue damage. The plates were incubated at 28.5 °C in the dark for 96 h, and 50% of the medium was replaced daily. At 108 hpf, 15 embryos from each group were randomly selected and imaged using a fluorescence microscope. Neutrophils were labeled with green fluorescence; fluorescence intensity positively correlates with neutrophil activation, increasing under inflammatory conditions. A reduction in fluorescence, therefore, indicates an anti-inflammatory effect. Fluorescence intensity was quantified with ImageJ software and used to evaluate the anti-inflammatory efficacy of each group. Statistical analysis and graphing were performed using GraphPad Prism.

Method S4. Anti-aging assays

The anti-aging evaluation comprised four assays: ROS level, SOD activity, MDA content, and senescence-associated β-galactosidase (SA-β-gal) staining.

Oxidative stress model for ROS, SOD, and MDA measurements

For antioxidant capacity evaluation, an oxidative stress model was generated by treating the embryos with 0.22 mmol/L H_2O_2. Eight groups were established: Control (untreated); MD (H_2O_2 alone, model group); SL (0.0001 mg/mL SL-EXO with H_2O_2); SM (0.0005 mg/mL SL-EXO with H_2O_2); SH (0.001 mg/mL SL-EXO with H_2O_2); RL (0.0001 mg/mL resveratrol with H_2O_2); RM (0.0005 mg/mL resveratrol with H_2O_2); RH (0.001 mg/mL resveratrol with H_2O_2). Each group contained 45 embryos, which were further divided into three subgroups (15 embryos per subgroup) for the separate determination of ROS, SOD, and MDA.

At 72 hpf, the larvae were stained with the DCFH-DA probe for ROS detection, and the mean fluorescence intensity was quantified using ImageJ. SOD activity and MDA content were measured using commercial assay kits according to the manufacturers' protocols.

Senescence-associated β-galactosidase staining

Pigmentation was suppressed by treating embryos with PTU. A DNA-damage-induced senescence model was established using 0.5 μmol/L doxorubicin, which activates the senescence-associated secretory phenotype (SASP). The following eight groups were set: Control (untreated); MD (doxorubicin alone, model group); SL (0.0001 mg/mL SL-EXO with doxorubicin); SM (0.0005 mg/mL SL-EXO with doxorubicin); SH (0.001 mg/mL SL-EXO with doxorubicin); RL (0.0001 mg/mL resveratrol with doxorubicin); RM (0.0005 mg/mL resveratrol + doxorubicin); RH (0.001 mg/mL resveratrol with doxorubicin). At 96 hpf, embryos were collected, fixed, and stained for SA-β-gal activity. The blue-stained area was quantified with ImageJ to assess cellular senescence.

Method S5. Zebrafish whitening assay

Seven groups were established: blank control (untreated), SL-EXO at 0.0001, 0.0005, and 0.001 mg/mL, and the positive control α-arbutin at the same three concentrations. Wild-type zebrafish embryos were used for all whitening assessments. For morphological imaging, embryos were treated with the corresponding drugs from 12 hpf onward and cultured for 96 h, after which 15 embryos per group were randomly selected and photographed under a microscope. For the determination of melanin content and tyrosinase activity, embryos were similarly exposed to the drugs from 12 hpf for 48 h. Ten larvae per group were then collected, blotted dry, and

homogenized in 200 μL of lysis buffer containing 1% (v/v) protease inhibitor cocktail. The homogenate was centrifuged at 4000 rpm for 10 min. The pellet was resuspended in 250 μL of 1 M NaOH containing 10% DMSO and incubated at 85 °C for 2 h to solubilize melanin; 100 μL of the resulting solution was transferred to a 96-well plate, and the absorbance was measured at 405 nm. For tyrosinase activity, 20 μL of the supernatant was mixed with 180 μL of L-DOPA solution (1 mg/mL) and incubated in the dark at 37 °C for 2 h; 100 μL of the reaction mixture was then transferred to a 96-well plate and the absorbance was read at 475 nm. Three independent replicates were performed for the melanin and tyrosinase assays.

Method S6. Metabolomics Analysis

Sample preparation and extraction

Biological samples were freeze-dried using a lyophilizer (Scientz-100F) and then ground to a fine powder with a grinder (MM 400, Retsch) at 30 Hz for 1.5 min. A 30 mg aliquot of the powder was weighed (MS105DM balance) and mixed with 1500 μL of −20 °C pre-cooled 70% aqueous methanol containing the internal standard (for samples weighing less than 30 mg, the extractant volume was scaled proportionally, i.e., 1500 μL per 30 mg). The mixture was vortexed for 30 s every 30 min, for a total of 6 cycles. After centrifugation at 12,000 rpm for 3 min, the supernatant was collected and filtered through a 0.22 μm microporous membrane into an injection vial for UPLC-MS/MS analysis.

UPLC conditions

The extracts were analyzed on an ExionLC™ AD UPLC system coupled to a triple quadrupole-linear ion trap (QTRAP) mass spectrometer (SCIEX). Chromatographic separation was performed on an Agilent SB-C18 column (1.8 μm, 2.1 mm × 100 mm). The mobile phase consisted of water containing 0.1% formic acid (solvent A) and acetonitrile containing 0.1% formic acid (solvent B). The gradient program was as follows: 0 min, 95% A/5% B; 9 min, 5% A/95% B; held at 5% A/95% B for 1 min; returned to 95% A/5% B in 1.1 min and held for 2.9 min. The flow rate was 0.35 mL/min, the column oven temperature was 40 °C, and the injection volume was 2 μL. The effluent was directed into the ESI source of the QTRAP-MS.

ESI-QTRAP-MS/MS parameters

The ESI source was operated with the following parameters: source temperature, 500 °C; ion

spray voltage, 5500 V (positive ion mode) / −4500 V (negative ion mode); ion source gas I, gas II, and curtain gas were set at 50, 60, and 25 psi, respectively; collision-activated dissociation (CAD) gas was set to high. MRM scans were acquired using nitrogen as the collision gas (medium setting). The declustering potential (DP) and collision energy (CE) were optimized for individual MRM transitions. A predefined set of MRM transitions was monitored in each period, based on the metabolites eluting within that time window.

Metabolite identification and quantification

Metabolites were identified by matching the MS/MS spectra against an in-house database (MWDB, Metware Database). During the identification process, isotopic signals, adduct ions (K^+, Na^+, NH_4^+), and fragment ions derived from larger molecules were excluded. Quantification was performed using the multiple reaction monitoring (MRM) mode of the triple quadrupole mass spectrometer. In MRM mode, the first quadrupole selects the precursor ion of the target metabolite, which is then fragmented in the collision cell, and the third quadrupole filters a characteristic product ion, thus eliminating interferences and improving accuracy and reproducibility. After data acquisition, chromatographic peaks of all metabolites were integrated, and the peak areas of the same metabolite across different samples were corrected to ensure consistency.

Screening of differential metabolites

Given the high-dimensional and massive nature of metabolomics data, a combination of univariate and multivariate statistical analyses was employed. Univariate methods included hypothesis testing and fold-change (FC) analysis. Multivariate analyses comprised principal component analysis (PCA) and orthogonal partial least squares-discriminant analysis (OPLS-DA). Based on the OPLS-DA model (with at least three biological replicates), the variable importance in projection (VIP) was used to initially identify metabolites that differed between groups. Subsequently, the P-value/FDR (for at least two biological replicates) and fold change were applied to further refine the selection. For this study, metabolites were considered significantly different if they met the following criterion: fold change ≥ 2 or ≤ 0.5 (i.e., at least a two-fold up- or down-regulation between the compared groups).

Method S7. Proteomics Analysis

Sample preparation

Protein extraction

Samples were removed from −80 °C and thawed on ice. Phenylmethylsulfonyl fluoride (PMSF) was added to a final concentration of 1 mM, and the mixture was centrifuged at 4500 g for 10 min at 4 °C. The supernatant was collected, and the protein concentration was determined using a BCA assay kit (Beyotime Biotechnology, Shanghai, China). If the protein concentration was too low, the supernatant was transferred to a 10 kDa ultrafiltration device (Beckman Colter Inc., Brea, CA) and concentrated by repeated ultrafiltration; the retentate was then collected, and the protein concentration was re-measured with the BCA kit.

Protein digestion and desalting

An aliquot containing 100 µg of protein was diluted to 200 µL with 8 M urea. Dithiothreitol (DTT) was added to a final concentration of 5 mM, and the sample was reduced at 37 °C for 45 min. Subsequently, iodoacetamide was added to a final concentration of 11 mM, and alkylation was carried out in the dark at room temperature for 15 min. The solution was then diluted with 800 µL of 25 mM ammonium bicarbonate, and 2 µL of trypsin (Promega, V5280) was added. Digestion was performed overnight at 37 °C. After digestion, the pH of the peptide mixture was adjusted to 2–3 with 20% trifluoroacetic acid (TFA), and the peptides were desalted using C18 spin columns (Millipore, Billerica, MA). The peptide concentration was determined using the Pierce™ Quantitative Peptide Assay (Thermo Fisher Scientific).

LC-MS/MS analysis

Nano-LC separation

Peptide samples were separated on a Vanquish Neo UHPLC system (Thermo Scientific). Mobile phase A was 0.1% formic acid in water, and mobile phase B was 0.1% formic acid in acetonitrile. The trap-and-elute configuration employed a PepMap Neo Trap Cartridge (300 µm × 5 mm, 5 µm) and an Easy-Spray™ PepMap™ Neo UHPLC analytical column (150 µm × 15 cm, 2 µm). The column temperature was maintained at 55 °C. A total of 200 ng of peptides was loaded, and the flow rate was set at 2.5 µL/min. The effective gradient length was 22 min, with a total run time of 24 min.

Orbitrap Astral mass spectrometry

Data-independent acquisition (DIA) was performed on an Orbitrap Astral high-resolution mass

spectrometer (Thermo Scientific) coupled to the nano-LC system. Mass spectra were acquired in positive ion mode over the *m/z* range 380–980. MS1 spectra were recorded at a resolution of 240,000 (at 200 *m/z*) with a normalized AGC target of 500% and a maximum injection time of 5 ms. MS2 spectra were acquired in DIA mode using 299 isolation windows of 2 Th each. HCD fragmentation was performed with a collision energy of 25%, and the normalized AGC target for MS2 was set to 500% with a maximum injection time of 3 ms.

Mass spectrometry data analysis

Principle of database search

Mass spectrometric analysis yields the mass-to-charge ratios (*m/z*) and intensities of peptides (MS1 spectra) and their fragment ions (MS2 spectra). Owing to the complexity of the spectral data, database searching is required to interpret peptide sequences. Prior to the search, a theoretical MS2 spectral library is constructed from the protein sequences in the selected database. The experimental MS2 spectra are then matched against this library, and peptide sequences are assigned after scoring and filtering. Proteins are inferred from the identified unique peptides.

Data processing

Database searching is computationally intensive and requires specialized software. In this p roject, the DIA data were analyzed using DIA-NN (v1.8.1) in library-free mode. The searc h was performed against the uniprotkb_taxonomy_id_49669_2026_02_01_pp.fasta database (containing 3,554 protein sequences). The deep-learning-based spectrum prediction option w as enabled to generate an in silico spectral library. Additionally, the Match Between Runs (MBR) feature was activated to construct a spectral library from the DIA data, which was subsequently used for re-analysis to obtain both qualitative and quantitative protein results. Precursor and protein-level identifications were filtered

Method S8. Lipidomics Analysis

Sample preparation

All samples were freeze-dried and ground to a fine powder. After thawing, 0.02000 ± 0.00100 g of the ground dry sample was weighed into a 2 mL centrifuge tube, and one steel ball (approximately 4 mm diameter) was added. One milliliter of lipid extraction solvent (methyl tert-butyl ether : methanol = 3:1, v/v) was added, and the mixture was shaken at 2500 rpm for 15 min at

room temperature. Then, 300 μL of ultrapure water was added, vortexed for 1 min, and the tube was left standing at 4 °C for 10 min. After centrifugation at 12,000 rpm for 10 min at 4 °C, 200 μL of the supernatant was transferred to a 1.5 mL centrifuge tube and concentrated to dryness under vacuum at −20 °C for 2 h. The residue was reconstituted in 200 μL of lipid reconstitution solvent (acetonitrile : isopropanol = 1:1, v/v), vortexed for 3 min, and centrifuged at 12,000 rpm for 3 min at 4 °C. Finally, 120 μL of the supernatant was transferred to a glass insert in an autosampler vial for LC-MS/MS analysis.

UPLC and MS conditions

The analytical system consisted of an ExionLC™ AD UPLC system coupled to a QTRAP® 6500+ tandem mass spectrometer (SCIEX). Chromatographic separation was performed on a Thermo Accucore™ C30 column (2.6 μm, 2.1 mm × 100 mm i.d.). The mobile phase consisted of (A) acetonitrile/water (60/40, v/v) containing 0.1% formic acid and 10 mmol/L ammonium formate, and (B) acetonitrile/isopropanol (10/90, v/v) containing 0.1% formic acid and 10 mmol/L ammonium formate. The gradient program was as follows: 0 min, 80% A/20% B; 2 min, 70% A/30% B; 4 min, 40% A/60% B; 9 min, 15% A/85% B; 14 min, 10% A/90% B; 15.5 min, 5% A/95% B; held at 5% A/95% B until 17.3 min; returned to 80% A/20% B at 17.5 min and maintained until 20 min. The flow rate was 0.35 mL/min, the column temperature was 45 °C, and the injection volume was 2 μL.

Mass spectrometric detection was performed using an electrospray ionization (ESI) source operated at 500 °C with an ion spray voltage of 5500 V in positive mode and −4500 V in negative mode. The nebulizer gas (GS1), auxiliary gas (GS2), and curtain gas (CUR) were set at 45, 55, and 35 psi, respectively. Each ion pair was monitored in multiple reaction monitoring (MRM) mode using optimized declustering potential (DP) and collision energy (CE).

Lipid identification and quantification

Lipids were identified by matching retention times and precursor-product ion pairs against an in-house database (MWDB). Quantification was carried out in MRM mode, where the first quadrupole selects the precursor ion of the target lipid, the collision cell fragments the precursor, and the third quadrupole filters a characteristic product ion, thereby improving selectivity and reproducibility. After data acquisition, chromatographic peaks were integrated, and quantification

was performed using the internal standard method.

Method S9. Network Pharmacology Analysis

Screening of active component targets

Structural files for selected chemical constituents were obtained from the PubChem database (https://pubchem.ncbi.nlm.nih.gov/) and saved in SDF format. These structure files were then uploaded to the SwissTargetPrediction database (http://www.swisstargetprediction.ch/) to retrieve potential targets of the chemical constituents. Targets with a prediction probability ≥ 0 were retained. After removing duplicates, the target names were standardized to official gene symbols using the UniProt database (https://www.uniprot.org/), yielding the active component targets.

Collection and screening of pigmentation targets and construction of the active component–target network

Disease targets related to pigmentation were collected by searching the GeneCards database (https://www.genecards.org) and the DisGeNET database using the keyword "pigmentation". In GeneCards, targets with a relevance score ≥ 1 were selected, while in DisGeNET, targets with a score ≥ 0 were retained. The active component targets obtained in 2.10.1 were then mapped onto the pigmentation-related disease targets, and the overlapping targets were considered potential therapeutic targets for pigmentation. A Venn diagram was generated using an online tool to visualize the overlap. Subsequently, a drug-component-target network was constructed using Cytoscape 3.7.1.

Construction of the protein–protein interaction (PPI) network of pigmentation targets

The overlapping targets were imported into the STRING online platform (https://string-db.org) with the organism set to Homo sapiens and a confidence score > 0.4 to construct a PPI network. The resulting data were loaded into Cytoscape 3.7.1, and the BisoGenet and CytoNCA plugins were used to analyze the drug and disease targets. Core network targets were identified through topological analysis, including degree, betweenness centrality, and closeness centrality. The node size in the network was scaled according to the degree value.

GO enrichment and KEGG pathway analysis

Gene Ontology (GO) enrichment and Kyoto Encyclopedia of Genes and Genomes (KEGG)

pathway enrichment analyses were performed on the potential targets using DAVID (https: //david.ncifcrf.gov/). The identifier was set to "OFFICIAL_GENE_SYMBOL" and the speci es to Homo sapiens. The results were visualized using an online bioinformatics platform (http://www.bioinformatics.com.cn/).

Method S10. Cell viability assay

When reaching 80-90% confluence, B16F10 cells were trypsinized, resuspended in fresh medium, and passaged at a 1:3 ratio. For the assay, cells were seeded into 96-well plates at a density of 1×10^5 cells/mL (100 μL/well) and allowed to attach overnight. Stock solutions of SL-EXO, RP-EXO, and α-arbutin were freshly prepared in complete medium at five concentrations: 0.0025, 0.015, 0.025, 0.075, and 0.15 mg/mL (expressed as protein concentration for PDNVs). After removing the culture supernatant, each well received 100 μL of the corresponding sample-containing medium; a normal control group received an equal volume of complete medium. Three replicate wells were set per concentration. After 24 h or 48 h of incubation (37 °C, 5% CO_2), the medium was discarded, and 100 μL of medium containing 10% CCK-8 was added to each well. After a further 2 h of incubation at 37 °C, the absorbance at 450 nm was measured using a microplate reader. Cell viability (%) was calculated relative to PBS-treated controls using the formula:

$$\text{Cell viability (\%)} = A_{sample} - A_{blank} / A_{control} - A_{blank}$$

where A_{sample}, $A_{control}$ and A_{blank} represent the absorbance values of PDNVs-treated wells, PBS-treated wells, and cell-free blank wells, respectively.

Method S11. Cellular uptake

PKH67 (2 μL) was diluted in 10 μL PBS to prepare the working solution. Then, 50 μL PDNVs suspension or α-arbutin was incubated with PKH67 for 10 min at RT (room temperature) in the dark. Unbound dye was removed by centrifugation at 12,000 × g for 10 min using a 100 kDa filter.

B16F10 cells were seeded in 24-well plates in DMEM with 10% FBS. After reaching 60% confluence, cells were serum-starved overnight in FBS-free DMEM to synchronize them. Labeled PDNVs or α-arbutin were added and incubated at 37 °C with 5% CO_2 for 24 h.

Post-incubation, cells were washed with PBS, fixed with 4% paraformaldehyde for 15 min at RT, and permeabilized with 0.2% Triton X-100 for 10 min at RT. Then, cells were incubated with Alexa Fluor 488-phalloidin (2 drops/mL in PBS) for 30 min at 37 °C in the dark, followed by PBS washes.

After aspirating phalloidin, nuclei were stained with 1 μg/mL DAPI for 5 min. Excess DAPI was washed with PBS, and coverslips were mounted with antifade medium. Internalization of PDNVs and the cytoskeleton was visualized by confocal microscopy (excitation/emission: 488/525 nm for PKH67, 405/461 nm for DAPI).

Method S12. Determination of melanin content and tyrosinase activity in B16F10 cells

The experimental groups were as follows: blank control (DMEM containing 0.1% DMSO, without α-MSH); model control (DMEM containing 200 nM α-MSH and 0.1% DMSO); positive control groups (α-MSH with α-arbutin at 0.0025, 0.015, and 0.025 mg/mL); sample treatment groups (α-MSH with SL-EXO or RP-EXO at 0.0025, 0.015, and 0.025 mg/mL, expressed as protein concentration). Except for the blank control, all groups were pre-treated with 200 nM α-MSH in DMEM containing 0.1% DMSO for 1 h (37 °C, 5% CO_2) to stimulate melanogenesis. After pre-treatment, the medium was replaced with fresh medium containing the corresponding samples, and the cells were incubated for an additional 48 h.

For tyrosinase activity, cells were washed twice with ice-cold PBS, lysed with ice-cold PBS containing 1% Triton X-100 (50-100 μL/well) on ice for 30 min, and centrifuged at 12,000 rpm for 10 min at 4 °C. A portion of the supernatant was mixed with an equal volume of L-DOPA solution (1 mg/mL) and incubated in the dark at 37 °C for 2 h. The absorbance was then measured at 475 nm. The protein concentration in the supernatant was determined using a BCA assay kit to normalize tyrosinase activity. For melanin content, parallel wells were prepared, and after the 48-h treatment, cells were lysed with 250 μL of 1 M NaOH containing 10% DMSO at 80 °C for 1 h to dissolve melanin. The lysate was cooled to room temperature and centrifuged at 12,000 rpm for 10 min to remove insoluble debris. The absorbance of the supernatant was measured at 405 nm

Method S13. Western Blot Analysis

Cell Treatment and Grouping

To elucidate the underlying molecular mechanisms, B16F10 cells were divided into four

experimental groups: Control group(cells cultured under normal conditions); Model group(cells stimulated with α-MSH to induce melanogenesis); Drug (SL-EXO) group(cells co-treated with α-MSH and 0.025 mg/mL SL-EXO); and Rescue group(cells pre-treated with 10 μM 740 Y-P (a specific PI3K/AKT agonist) for 1 h prior to the co-treatment of α-MSH and 0.025 mg/mL SL-EXO).

Protein Extraction and Quantification

Following the respective treatments, the cells were harvested and lysed in cold RIPA lysis buffer supplemented with 1% PMSF and a protease inhibitor cocktail on ice to prevent protein degradation. The cell lysates were then centrifuged at 12,000 rpm for 15 min at 4 °C to remove cellular debris. The total protein concentration of the resulting supernatant was quantified using a BCA protein assay kit.

SDS-PAGE and Immunoblotting

SDS-PAGE by 7.5% or 12% polyacrylamide gels at a constant voltage of 150V for 50 min, followed by separation of equal amounts of total protein (30 μg per lane) plus 3μL of a prestained protein marker. The resolved proteins were subsequently transferred at a constant current of 240 mA for 50 min to the methanol-activated membranes. Membranes were washed 3 times with TBST (5 min each) to prevent nonspecific binding and blocked with blocking buffer for 2 h at room temperature. The membranes were incubated with specific antibodies overnight at 4 °C at a dilution of 1:1000. Following three TBST washes, membranes were incubated with 1:5000 HRP-conjugated secondary antibodies for 1 h at room temperature. Following three additional washes with TBST, the immunoreactive protein bands were visualized using an enhanced chemiluminescence (ECL) detection reagent. The luminescent signals were captured using a gel imaging system, and the exposure settings were adjusted to achieve optimal background contrast. Relative densitometric quantification of the protein bands was performed using ImageJ, with β-actin as the internal loading control.

Method S14. Quantitative Real-Time PCR (RT-qPCR) Analysis

Total RNA Extraction

TRIzol dry reagent was used to extract total RNA from treated B16F10 cells according to the standard phase-separation protocol. In short, cell lysates mixed with chloroform (200 μL for 1

mL TRIzol) were centrifuged at 12,000 rpm for 15 min at 4 °C. The upper aqueous phase was carefully collected, and RNA was precipitated by adding an equal volume of isopropanol (incubated at 4 °C for 10 min). After centrifugation at 12000 rpm for 10 min at 4 °C, we washed the RNA pellet with 75% ethanol and air-dried, then dissolved it in 20 μL RNase-free water. The RNA was quantified for concentration and purity using a microvolume spectrophotometer.

Reverse Transcription and qPCR

The purified RNA was converted to complementary DNA (cDNA) using a commercial reverse transcription kit. The thermal cycling conditions for cDNA synthesis were 37 °C for 2 min, 50 °C for 15 min, and an enzyme inactivation step at 85 °C for 2 min. Subsequently, qPCR was performed on a Roche fluorescence quantitative PCR system using a 384-well plate. The specific forward and reverse primer sequences for the target genes (MITF, TYR, TRP-1) and the internal control (β-actin) are provided in Table S9. All qPCR reactions were done in independent triplicates. The transcription levels of relative mRNA were calculated using $2^{-\Delta\Delta Ct}$, normalized to β-actin.

Method S15. Molecular Docking and Interaction Visualization

Preparation of Ligands and Protein Receptors

The 2D chemical structures of the selected small-molecule ligands (i.e., the hyper-accumulated metabolites in SL-EXO) were retrieved from the PubChem database. The corresponding 3D structures were subsequently constructed and energy-minimized using Chem3D (version 23.1.1) to achieve geometrically stable conformations, which were then saved in the .mol2 format.

For the protein receptor, the high-resolution crystal structure of the core hub target (AKT1, (PDB ID: 4GV1, Resolution: 1.49 Å) was obtained from the RCSB Protein Data Bank (PDB). Prior to docking, the raw protein structure was rigorously prepared in PyMOL (version 2.6). This preprocessing included the removal of co-crystallized water molecules, redundant ligands, and extraneous ions (e.g., phosphate groups) to isolate the pure receptor, which was subsequently exported in the standard .pdb format. Furthermore, we performed a re-docking validation protocol using the co-crystallized native ligand present in the original PDB structure. The Root Mean Square Deviation (RMSD) between the docked pose and the experimental native conformation was calculated to be 0.188 Å. This confirms the validity and geometric accuracy of our docking

parameters. These validation details have now been formally incorporated into the Supplementary Methods.

Molecular Docking Simulation and Visualization

The preprocessed protein and ligands were further refined using AutoDock Tools. The preparation process involved adding polar hydrogen atoms, assigning Gasteiger charges, and defining rotatable bonds for the ligands.

Molecular docking simulations were executed utilizing AutoDock Vina (version 1.2.5). A customized grid box was precisely configured—defining the optimal coordinates and dimensions—to enclose the entire active site pocket of the protein receptor. The best docking conformations were screened and ranked based on their binding free energy scores (ΔG, kcal/mol). Finally, to elucidate the specific spatial-atomic interactions (e.g., hydrogen bonds, hydrophobic interactions, and Pi effects), the optimal ligand-receptor docking complexes were visually rendered as 2D interaction diagrams and 3D structural models using Discovery Studio (version 2019) and PyMOL (version 2.6). The coordinates of the grid box center were set to X = -26.576, Y = 2.821, and Z =16.228. The dimensions of the search space were configured as follows:

PDB ID	Protein target	Compounds/ligand	Center of the box	Dimensions of the box (Å)
4GV1	AKT1	4-p-Coumaroylquinic acid	x= -26.576<br>y= 2.821<br>z= 16.228	x= 42.75<br>y= 42.75<br>z= 43.5
		BOA-6-O-glucoside<br>Cyanidin 3-O-(6-O-p-coumaroyl)glucoside<br>Kaempferol-3-O-glucoside-7-O-rhamnoside<br>Uridine Monophosphate<br>Cogformer		x= 33.75<br>y= 33.75<br>z= 33.75
		chrysophanol-8-o-beta-d-glucoside		x= 47.25<br>y= 47.25<br>z= 47.25

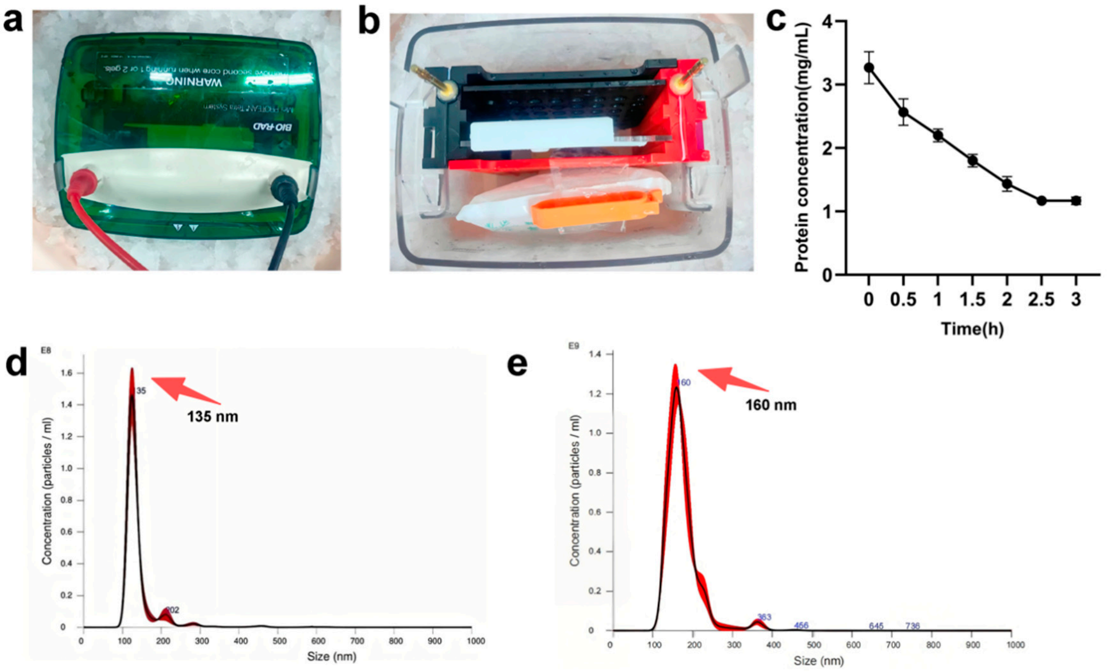

Figure S1. Comparison of PDNVs (SL-EXO) isolated by PEG+ED and ultracentrifugation (UC). (a) Photograph of the electrophoretic dialysis apparatus used for PDNV isolation. (b) Internal view of the electrophoretic dialysis setup, showing the dialysis bag placed within the chamber. (c) Nanoparticle tracking analysis (NTA) size distribution profile of PDNVs (SL-EXO) isolated by the PEG+ED method. (d) NTA size distribution profile of PDNVs (SL-EXO) isolated by conventional ultracentrifugation (UC).

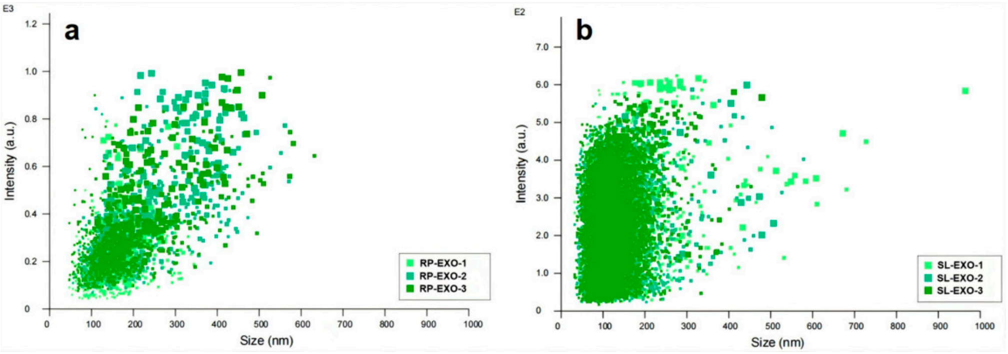

Figure S2. Intensity/Size distribution from three replicate NTA measurements of RP-EXO (a) and SL-EXO (b).

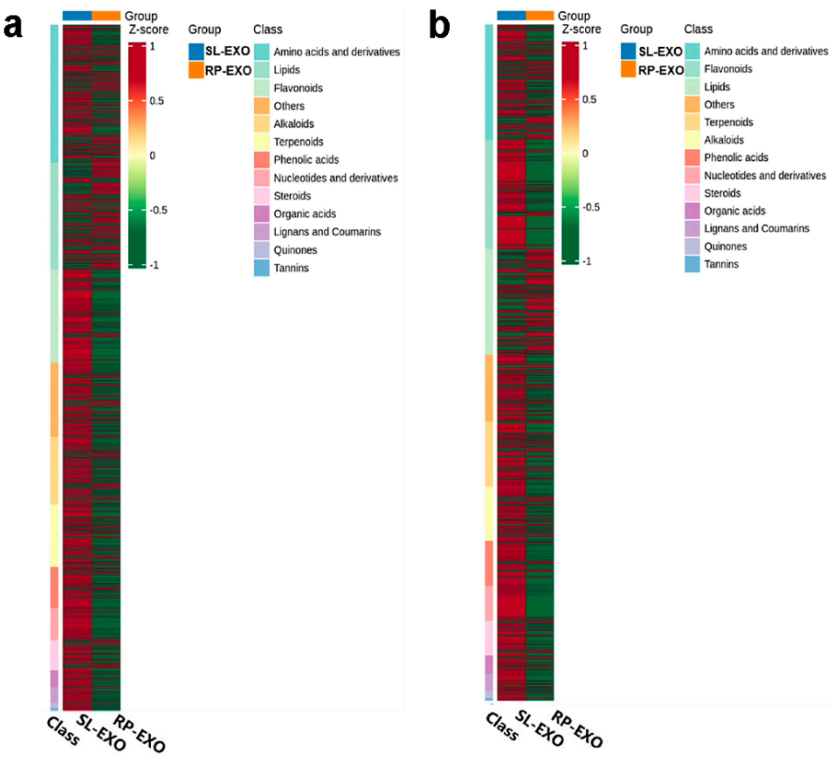

Figure S3. Heatmap visualization of metabolite profiles. (a) Heatmap of all detected metabolites. (b) Heatmap of differential metabolites between groups. In both panels, rows indicate metabolites, columns indicate individual samples with group labels, and colors represent normalized relative contents (red: high, green: low).

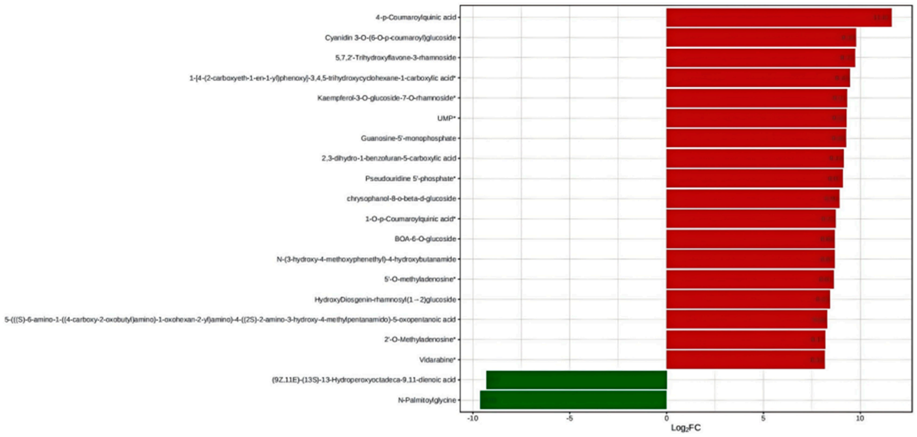

Figure S4. Top 20 differential metabolites by log_2FC. Horizontal axis: log_2(fold change) (log_2FC); vertical axis: metabolite names. Red bars represent up-regulated metabolites, green bars represent down-regulated metabolites.

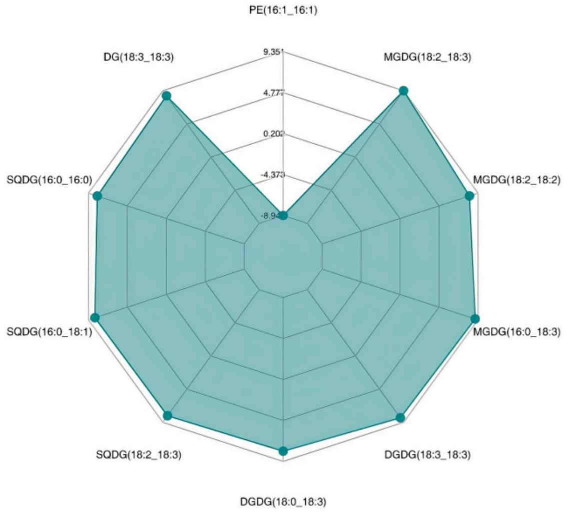

Figure S5. Radar chart of differential lipids. Each radial axis represents a differential lipid. The grid lines indicate the magnitude of fold change values. The green shaded area is formed by connecting the fold change values of the individual lipids, with the distance from the center to each data point representing the corresponding fold change. A larger shaded area indicates greater overall changes in lipid levels.

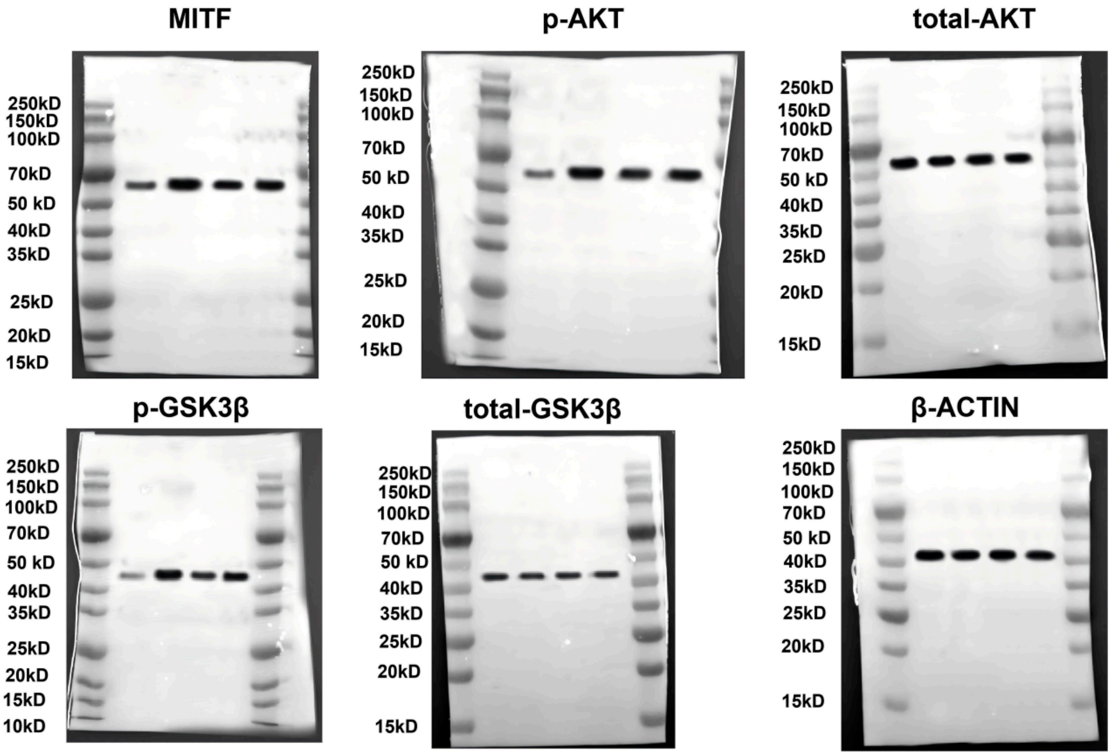

Figure S6. Raw western blot data.

Table S1. Pipetting layout for the tyrosinase activity inhibition assay.

Reagent	Solvent blank well (T_a)	Solvent reaction well (T_b)	Sample blank well (T_c)	Sample reaction well (T_d)
L-Tyrosine solution (μL)	0	40	0	40
Sample solution (μL)	0	0	40	40
Solvent (PBS buffer) (μL)	40	40	0	0
PBS buffer (μL)	70	30	70	30
Tyrosinase solution (μL)	20	20	20	20
Total (μL)	130	130	130	130

Table S2. Representative Compounds from the Top 100 for RP-EXO.

**Number**	**Compounds**	**Formula**	**RP-EXO**
1	cis-3-Hydroxy-L-proline*	$C_5H_9NO_3$	183166042.5
2	cis-4-Hydroxy-D-proline*	$C_5H_9NO_3$	168866346.2
3	N(6),N(6)-Dimethyl-L-lysine	$C_8H_{18}N_2O_2$	151976265.5
4	2-Amino-4-dihydroxy octadecyl galactoside	$C_{24}H_{49}NO_8$	147324550.6
5	6-(6-aminohexanoylamino)hexanoate*	$C_{12}H_{24}N_2O_3$	112569004.1
6	LysoPC 18:3(2n isomer)*	$C_{26}H_{48}NO_7P$	95228182.04
7	D-Allo-Isoleucine*	$C_6H_{13}NO_2$	75982775.96
8	L-Isoleucine*	$C_6H_{13}NO_2$	75721575.79
9	L-Leucine*	$C_6H_{13}NO_2$	74696093.73

Number	Compounds	Formula	RP-EXO
10	Citrate	$C_6H_8O_7$	73648481.54
11	Senkyunolide Q*	$C_{16}H_{22}O_4$	73093222.55
12	L-Arginine	$C_6H_{14}N_4O_2$	72699159.81
13	L-Glutamine	$C_5H_{10}N_2O_3$	72025359.77
14	L-Lysine*	$C_6H_{14}N_2O_2$	70523182.41
15	L-Norleucine*	$C_6H_{13}NO_2$	70139232.04
16	Ile-gly	$C_8H_{16}N_2O_3$	68676855.86
17	Methylillicinone F*	$C_{16}H_{22}O_4$	60086173.96
18	Glycyl-leucine*	$C_8H_{16}N_2O_3$	59580084.06
19	Leu-Pro	$C_{11}H_{20}N_2O_3$	57980889.08
20	Isoleucylleucine*	$C_{12}H_{24}N_2O_3$	56683938.01
21	Choline	$C_5H_{14}NO+$	53497412.04
22	L-Leucyl-L-Leucine*	$C_{12}H_{24}N_2O_3$	52680771.6
23	L-Glycyl-L-isoleucine*	$C_8H_{16}N_2O_3$	49577499.91
24	2,5-diaminohexanoic acid*	$C_6H_{14}N_2O_2$	49041478.41
25	(3β,8β,14α,21β)-26,27-dinoronocerane-3,8,14,21-tetrol	$C_{29}H_{52}O_4$	48738914.7
26	8-{4-Hydroxy-3,3-Dimethylbicyclo[4.1.0]Heptan-2-Yl}-6-Methyloct-5-En-2-one	$C_{18}H_{30}O_2$	47114868.35
27	Punicic acid	$C_{18}H_{30}O_2$	47045967.24
28	Leucyl-Isoleucine	$C_{12}H_{24}N_2O_3$	46821716.45
29	Brisbenone	$C_{27}H_{40}O_3$	46746544.93
30	Octadeca-9,12,15-trienoic acid	$C_{18}H_{30}O_2$	42716956.17
31	1-aminobenzocyclobutene	C_8H_9N	42020578.53
32	Histidylleucine	$C_{12}H_{20}N_4O_3$	39837687.7
33	Leucylalanine	$C_9H_{18}N_2O_3$	38414574.95
34	Estrane-3,17-diol	$C_{18}H_{30}O_2$	37309965.15
35	2-methyl-1-nitronaphthalene	$C_{11}H_9NO_2$	37045317.31
36	Inosine*	$C_{10}H_{12}N_4O_5$	36965870.02
37	D-2-Aminobutyrate	$C_4H_9NO_2$	36924704.24
38	8-(5-Hydroxy-2,6,6-Trimethylcyclohex-2-En-1-Yl)-6-Methyloct-5-En-2-one	$C_{18}H_{30}O_2$	36734595.02
39	L-Leucyl-L-alanine	$C_9H_{18}N_2O_3$	36057863.8
40	L-Histidine*	$C_6H_9N_3O_2$	35846853.58
41	Phe-Ile	$C_{15}H_{22}N_2O_3$	34672258.49
42	(7S)-bicyclo[4.2.0]octa-1,3,5-trien-7-amine	C_8H_9N	34025730.57
43	Leu Ser Ile	$C_{15}H_{29}N_3O_5$	33147721.46

Number	Compounds	Formula	RP-EXO
44	LysoPC 16:1*	$C_{24}H_{48}NO_7P$	32038039.53
45	4-(1,2-Dihydroxypropan-2-yl)cyclohex-1-ene-1-carboxylic acid	$C_{10}H_{16}O_4$	31480742.85
46	Bicyclo[4.2.0]octa-1,3,5-trien-7-amine, (R)-	C_8H_9N	31250407.95
47	Ala-ile*	$C_9H_{18}N_2O_3$	30690333.06
48	2-(2,3-dihydroxypropoxy)-3-(((2-(dimethylamino)ethoxy)(hydroxy)phosphoryl)oxy)propyl (11Z,14Z)-octadeca-11,14-dienoate*	$C_{28}H_{54}NO_9P$	30614505.39
49	Allopurinol riboside	$C_{10}H_{12}N_4O_5$	30425073.82
50	(9S)-Hydroxyoctadecadienoic acid	$C_{18}H_{32}O_3$	30357793.02
51	sn-Glycero-3-phosphocholine	$C_8H_{20}NO_6P$	30187149.7
52	LysoPC 16:1(2n isomer)*	$C_{24}H_{48}NO_7P$	29641650.67
53	Hexadecanal	$C_{16}H_{32}O$	29561129.57
54	LysoPC 18:0	$C_{26}H_{54}NO_7P$	27921332.13
55	(2-aminoethoxy)({2-hydroxy-3-[(11E)-octadec-11-enoyloxy]propoxy})phosphinic acid*	$C_{23}H_{46}NO_7P$	27839917.57
56	N-(2-hydroxyethyl)-5,8,11,14-eicosatrienoic amide	$C_{22}H_{37}NO_2$	27763080.77
57	beta-alanyl-l-leucine*	$C_9H_{18}N_2O_3$	26954346.11
58	Diisobutyl phthalate*	$C_{16}H_{22}O_4$	26786592.93
59	LysoPE 18:1*	$C_{23}H_{46}NO_7P$	26528110.43
60	LysoPC 20:1	$C_{28}H_{56}NO_7P$	26228072.5
61	9-Oxo-10,12-Octadecadienoic Acid	$C_{18}H_{30}O_3$	25840065.49
62	3-Hydroxy-3,7,11-trimethyldodeca-1,6E,10-trien-9-yl isobutyrate	$C_{18}H_{30}O_3$	25452807.72
63	2-Aminohexadecane-1,4-diol	$C_{16}H_{35}NO_2$	25046759.66
64	(2-aminoethoxy)[2-hydroxy-3-(octadeca-9,12,15-trienoyloxy)propoxy]phosphinic acid*	$C_{23}H_{42}NO_7P$	24678371.94
65	naphthisoxazol A*	$C_{11}H_9NO_2$	24588000.04
66	L-Alanyl-L-leucine	$C_9H_{18}N_2O_3$	24422804.7
67	LysoPE 18:3*	$C_{23}H_{42}NO_7P$	24347119.88
68	LysoPE 16:1(2n isomer)*	$C_{21}H_{42}NO_7P$	24223834.48
69	LysoPE 18:3(2n isomer)*	$C_{23}H_{42}NO_7P$	24206150.48
70	Lauryldiethanolamine	$C_{16}H_{35}NO_2$	24161995.98
71	Isoinosine*	$C_{10}H_{12}N_4O_5$	23449991.43
72	Dodecanoic acid	$C_{12}H_{24}O_2$	23352358.87
73	LysoPE 16:1*	$C_{21}H_{42}NO_7P$	23239047.04
74	9,10,11-Trihydroxy-12-octadecenoic acid	$C_{18}H_{34}O_5$	21910857.98

Number	Compounds	Formula	RP-EXO
75	Ent-3β-Acetoxyisopimar-15-8β-ol*	$C_{22}H_{36}O_3$	21287805.12
76	1-(2,3-dihydroxypropoxy)-3-(((2-(dimethylamino)ethoxy)(hydroxy)phosphoryl)oxy)propan-2-yl (11Z,14Z)-octadeca-11,14-dienoate*	$C_{28}H_{54}NO_9P$	20522645.36
77	Viteagnusin B*	$C_{22}H_{36}O_3$	19910651.3
78	Hexadecylsphingosine	$C_{16}H_{35}NO_2$	19514646.11
79	L-Tryptophanamide	$C_{11}H_{13}N_3O$	19148289.7
80	LysoPC 20:2*	$C_{28}H_{54}NO_7P$	18385538.69
81	L-Alanyl-L-Phenylalanine	$C_{12}H_{16}N_2O_3$	17920551.36
82	2(R)-HOT	$C_{18}H_{30}O_3$	17769365.7
83	LysoPC 20:2(2n isomer)*	$C_{28}H_{54}NO_7P$	17527129.05
84	Tryptophan	$C_{11}H_{12}N_2O_2$	17322924.82
85	Phe-Ser	$C_{12}H_{16}N_2O_4$	17286372.87
86	LysoPC 18:0(2n isomer)	$C_{26}H_{54}No7P$	16694922.01
87	17-Hydroxyspirost-5-en-3-yl-2-O-[6-deoxymannosyl(1→2)]glucoside (Polyphyllin VI)	$C_{39}H_{62}O_{13}$	14940173.03
88	Deoxyarbutin	$C_{11}H_{14}O_3$	14897547.6
89	(2-aminoethoxy)({3-hydroxy-2-[(11E)-icos-11-enoyloxy]propoxy})phosphinic acid	$C_{25}H_{50}NO_7P$	14627493.53
90	Dextropimaric acid	$C_{18}H_{28}O_2$	14344280.12
91	LysoPE 18:1(2n isomer)*	$C_{23}H_{46}NO_7P$	14295347.45
92	Ser-Val-Leu	$C_{14}H_{27}N_3O_5$	14208797.15
93	9-(Arabinosyl)hypoxanthine	$C_{10}H_{12}N_4O_5$	13782286.35
94	6-Deoxyfagomine	$C_6H_{13}NO_2$	13319369.48
95	(9Z,11E)-(13S)-13-Hydroperoxyoctadeca-9,11-dienoic acid	C18H32O4	13186947.2
96	3-(Pyrazol-1-yl)-L-alanine*	$C_6H_9N_3O_2$	12618305.16
97	2-amino-2-(1-methyl-1H-pyrazol-4-yl)acetic acid	$C_6H_9N_3O_2$	12363157.31
98	LysoPE 18:2*	$C_{23}H_{44}NO_7P$	12213025.41
99	(2R)-2-azaniumyl-3-(4-chlorophenyl)propanoate	$C_9H_{10}C_lNO_2$	11997948.03
100	Epigoitrin	C_5H_7NOS	11857684.3

[1]*: There are isomers present in this substance.

Table S3. Representative Compounds from the Top 100 for SL-EXO

Number	Compounds	Formula	SL-EXO
1	cis-3-Hydroxy-L-proline*	$C_5H_9NO_3$	170977255.9
2	cis-4-Hydroxy-D-proline*	$C_5H_9NO_3$	156832400

3	6-(6-aminohexanoylamino)hexanoate*	$C_{12}H_{24}N_2O_3$	127205815.7
4	1-O-p-Coumaroylquinic acid*	$C_{16}H_{18}O_8$	123173307.7
5	LysoPC 18:3(2n isomer)*	$C_{26}H_{48}NO_7P$	120209260
6	Guanine*	$C_5H_5N_5O$	114729252.2
7	etheno-dC	$C_{11}H_{13}N_3O_4$	103590624.6
8	(2R,3R,4S)-2-(6-aminopurin-9-yl)-4-(hydroxymethyl)oxolan-3-ol*	$C_{10}H_{13}N_5O_3$	100901903.7
9	Cytidine	$C_9H_{13}N_3O_5$	94925707.04
10	Deoxyadenosine*	$C_{10}H_{13}N_5O_3$	93867272
11	Senkyunolide Q*	$C_{16}H_{22}O_4$	86150163.25
12	Isoleucylleucine*	$C_{12}H_{24}N_2O_3$	82052823.58
13	Glycyl-leucine*	$C_8H_{16}N_2O_3$	78991121.42
14	L-Leucine*	$C_6H_{13}NO_2$	77925885.97
15	L-Isoleucine*	$C_6H_{13}NO_2$	77814228.2
16	Methylillicinone F*	$C_{16}H_{22}O_4$	77776884.19
17	L-Leucyl-L-Leucine*	$C_{12}H_{24}N_2O_3$	76337339.7
18	D-Allo-Isoleucine*	$C_6H_{13}NO_2$	75797121.41
19	4,6-Dihydroxy-8-methyl-4,6,10-triethyltetradeca-2,7,11-trienoic acid	$C_{21}H_{36}O_4$	71246902.49
20	1-[4-(2-carboxyeth-1-en-1-yl)phenoxy]-3,4,5-trihydroxycyclohexane-1-carboxylic acid*	$C_{16}H_{18}O_8$	70762685.71
21	L-Norleucine*	$C_6H_{13}NO_2$	69424528.99
22	L-Tyrosine*	$C9H_{11}NO_3$	68789784.64
23	L-Glycyl-L-isoleucine*	$C_8H_{16}N_2O_3$	67578815.5
24	4-Hydroxybenzoate	$C_7H_6O_3$	62775451.34
25	chrysophanol-8-o-beta-d-glucoside	$C_{21}H_20O_10$	61830824.35
26	N(6),N(6)-Dimethyl-L-lysine	$C_8H_{18}N_2O_2$	60925081.3
27	5,7,2'-Trihydroxyflavone-3-rhamnoside	$C_{21}H_20O_10$	60160425.19
28	Choline	$C_5H_{14}NO+$	57507509.01
29	3-Hydroxy-L-phenylalanine*	$C_9H_{11}NO_3$	53955821.49
30	L-Tyrosinamide	$C_9H_{12}N_2O_2$	53148730.85
31	1-aminobenzocyclobutene	C_8H_9N	49860018.75
32	beta-alanyl-l-leucine*	$C_9H_{18}N_2O_3$	48330876.49
33	2-methyl-1-nitronaphthalene	$C_{11}H_9NO_2$	48244186.49
34	4-p-Coumaroylquinic acid	$C_{16}H_{18}O_8$	47391917.97
35	Leu-Pro	$C_{11}H_20N_2O_3$	46666277.63
36	Turanose	$C_{12}H_{22}O_{11}$	46085645.27

37	Ala-ile*	$C_9H_{18}N_2O_3$	45704424.02
38	L-Alanyl-L-leucine	$C_9H_{18}N_2O_3$	44774094.52
39	Histidylleucine	$C_{12}H_{20}N_4O_3$	41617434.26
40	Leucylalanine	$C_9H_{18}N_2O_3$	41450617.74
41	L-Leucyl-L-alanine	$C_9H_{18}N_2O_3$	38253899.87
42	4-(1,2-Dihydroxypropan-2-yl)cyclohex-1-ene-1-carboxylic acid	$C_{10}H_{16}O_4$	37870710.29
43	D-2-Aminobutyrate	$C_4H_9NO_2$	36420034.49
44	L-Glutamine	$C_5H_{10}N_2O_3$	34818136.84
45	(7S)-bicyclo[4.2.0]octa-1,3,5-trien-7-amine	C_8H_9N	34762612.72
46	L-Valine	$C_5H_{11}NO_2$	34749272.76
47	Allopurinol riboside	$C_{10}H_{12}N_4O_5$	34692065.68
48	Inosine*	$C_{10}H_{12}N_4O_5$	34498205.36
49	Bicyclo[4.2.0]octa-1,3,5-trien-7-amine, (R)-	C_8H_9N	32366863.35
50	L-Lysine*	$C_6H_{14}N_2O_2$	32277200.09
51	LysoPC 18:0	$C_{26}H_{54}NO_7P$	31817967.88
52	Glycerol 9,11,13-octadecatrienoyl ester*	$C_{21}H_{36}O_4$	31295090.56
53	1-Monolinolenoyl-Rac-Glycerol*	$C_{21}H_{36}O_4$	31151096.02
54	Diisobutyl phthalate*	$C_{16}H_{22}O_4$	31000325.53
55	Leu-Asp	$C_{10}H_{18}N_2O_5$	30424451.58
56	Leucyl-Isoleucine	$C_{12}H_{24}N_2O_3$	30208933.48
57	naphthisoxazol A*	$C_{11}H_9NO_2$	30126842.03
58	1-α-Linolenoyl-glycerol*	$C_{21}H_{36}O_4$	30094895.62
59	Cyanidin 3-O-(6-O-p-coumaroyl)glucoside	$C_{30}H_{27}O_{13}+$	29973463.84
60	Monolinolenin*	$C_{21}H_{36}O_4$	29874017.31
61	2-Oxo-2,3-dihydrofuran-5-acetate*	$C_6H_6O_4$	29447116.55
62	Guanosine-5'-monophosphate	$C_{10}H_{14}N_5O_8P$	29368137.43
63	Nuatigenin-3-O-glucoside	$C_{33}H_{52}O9$	29086067.11
64	[(3r,4r,6r)-4,6-diethyl-6-[(1e,4r,5e)-4-ethyl-2-methylocta-1,5-dien-1-yl]-1,2-dioxan-3-yl]acetic acid*	$C_{21}H_{36}O_4$	29016381.98
65	2-α-Linolenoyl-glycerol*	$C_{21}H_{36}O_4$	28878644.54
66	Kaempferol-3-O-robinobioside(Biorobin)*	$C_{27}H_{30}O_{15}$	28432456.69
67	Kaempferol-3-O-glucoside-7-O-rhamnoside*	$C_{27}H_{30}O_{15}$	28399327.11
68	L-Alanyl-L-Phenylalanine	$C_{12}H_{16}N_2O_3$	27732105.25
69	Ile-Gly-Ile	$C_{14}H_{27}N_3O_4$	27445482.03
70	Norartocarpetin*	$C_{15}H_{10}O_6$	26842909.4
71	Hexadecanal	$C_{16}H_{32}O$	24945173.74

72	Adenosine N1-oxide*	$C_{10}H_{13}N_5O_5$	24676432.07
73	Isoinosine*	$C_{10}H_{12}N_4O_5$	23943084.77
74	N-(3-hydroxy-4-methoxyphenethyl)-4-hydroxybutanamide	$C_{14}H_{21}NO_4$	23783121.04
75	Guanosine*	$C_{10}H_{13}N_5O_5$	23713979.75
76	6-amino-9-[(2R,3R,4S,5R)-3,4-dihydroxy-5-(hydroxymethyl)oxolan-2-yl]-8H-purin-2-one*	$C_{10}H_{13}N_5O_5$	23219538.73
77	Calyxanthone*	$C_{15}H_{10}O_6$	23126835.38
78	L-Tryptophanamide	$C_{11}H_{13}N_3O$	22969976.57
79	2,5-diaminohexanoic acid*	$C_6H_{14}N_2O_2$	22913765.05
80	Spongouridine	$C_9H_{12}N_2O_6$	22669405.89
81	Leu-Thr*	$C_{10}H20N_2O_4$	22401973.75
82	Hexaethylene glycol	$C_{12}H_{26}O_7$	22381860.97
83	8-Hydroxydeoxyguanosine 5'-monophosphate	$C_{10}H_{14}N_5O_8P$	22292243.59
84	1-[3,4-Dihydroxy-5-(hydroxymethyl)oxolan-2-yl]-4-hydroxy hydropyridin-2-one	$C_{10}H_{13}NO_6$	22131791.53
85	Isocitric Acid*	$C_6H_8O_7$	22053760.46
86	1-[3,4-dihydroxy-5-(hydroxymethyl)oxolan-2-yl]pyridine-2,4-dione	$C_{10}H_{13}NO_6$	21609078.98
87	Guanosine 3'-phosphate	$C_{10}H_{14}N_5O_8P$	21431990.56
88	Yamogenin 3-O-neohesperidoside	$C_{39}H_{62}O_{12}$	21188054.15
89	Vidarabine*	$C_{10}H_{13}N_5O_4$	21048499.91
90	Diosgenin-3-O-rhamnosyl(1→3)glucoside (Polyphyllin C)*	$C_{39}H_{62}O_{12}$	21039351.4
91	2-Aminohexadecane-1,4-diol	$C_{16}H_{35}NO_2$	20202572.92
92	Ile-gly	$C_8H_{16}N_2O_3$	20155887.43
93	Hydroxythreonine xyloside	$C_9H_{17}NO_8$	20095345.68
94	9-Arabinosyladenine*	$C_{10}H_{13}N_5O_4$	20022476.97
95	Lauryldiethanolamine	$C_{16}H_{35}NO_2$	20005527.58
96	N2-(1-Carboxyethyl)-L-arginine	$C_9H_{18}N_4O_4$	19810274.66
97	9,10,11-Trihydroxy-12-octadecenoic acid	$C_{18}H_{34}O_5$	19522076.93
98	Spirost-5-en-3-yl-[6-deoxymannosyl(1→2)]glucoside (Polyphyllin V)*	$C_{39}H_{62}O_{12}$	19247633.63
99	Pyroglutamyl-Isoleucine*	$C_{11}H_{18}N_2O_4$	19125883.09
100	9-Alpha-Ribofuranosyladenine*	$C_{10}H_{13}N_5O_4$	18591182.82

[1]*: There are isomers present in this substance.

Table S4. The top 18 upregulated differential metabolites with the maximum absolute Log2FC values between SL-EXO and RP-EXO

**Number**	**Compounds**	**Formula**	**CAS**	**Structure**

1	2'-O-Methyladenosine*	$C_{11}H_{15}N_5O_4$	2140-79-6	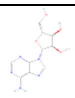
2	N-(3-hydroxy-4-methoxyphenethyl)-4-hydroxybutanamide	$C_{14}H_{21}NO_4$	-	
3	Cyanidin 3-O-(6-O-p-coumaroyl)glucoside	$C_{30}H_{27}O_{13}+$	-	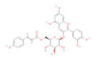
4	Kaempferol-3-O-glucoside-7-O-rhamnoside*	$C_{27}H_{30}O_{15}$	2392-95-2	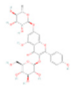
5	BOA-6-O-glucoside	$C_{13}H_{15}NO_8$	-	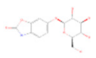
6	chrysophanol-8-o-beta-d-glucoside	$C_{21}H_{20}O_9$	13241-28-6	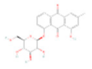
7	5,7,2'-Trihydroxyflavone-3-rhamnoside	$C_{21}H_{20}O_{10}$	-	
8	5'-O-methyladenosine*	$C_{11}H_{15}N_5O_4$	139468-46-5	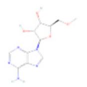
9	5-(((S)-6-amino-1-((4-carboxy-2-oxobutyl)amino)-1-oxohexan-2-yl)amino)-4-((2S)-2-amino-3-hydroxy-4-methylpentanamido)-5-oxopentanoic acid	$C_{22}H_{39}N_5O_9$	-	
10	Guanosine-5'-monophosphate	$C_{10}H_{14}N_5O_8P$	85-32-5	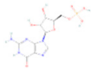
11	Vidarabine*	$C_{10}H_{13}N_5O_4$	5536-17-4	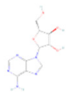
12	HydroxyDiosgenin-rhamnosyl(1→2)glucoside	$C_{39}H_{62}O_{13}$	-	
13	4-p-Coumaroylquinic acid	$C_{16}H_{18}O_8$	93451-44-6	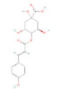
14	1-[4-(2-carboxyeth-1-en-1-yl)phenoxy]-3,4,5-trihydroxycyclohexane-1-carboxylic acid*	$C_{16}H_{18}O_8$	-	
15	2,3-dihydro-1-benzofuran-5-carboxylic acid	$C_9H_8O_3$	672-984-5<br>76429-73-7	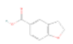
16	Pseudouridine 5'-phosphate*	$C_9H_{13}N_2O_9P$	1157-60-4	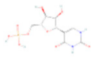
17	1-O-p-Coumaroylquinic acid*	$C_{16}H_{18}O_8$	-	
18	UMP*	$C_9H_{13}N_2O_9P$	58-97-9	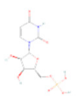

[1]*: There are isomers present in this substance.

Table S5. Lipid subclass distribution of identified lipids in SL-EXO and RP-EXO

Class	SL-EXO	RP-EXO
ADGGA	0	0
Cer	14	15
Cert	23	27
CoQ	1	1
DG	52	49
DGCC	0	1
DGDG	20	12
DGGA	1	1
DGTS	15	11
FFA	18	20
HexCer	14	15
LDGTS	4	3
LPA	5	4
LPC	20	21
LPE	7	9
LPG	5	5
LPI	3	4
LPS	1	3
MG	5	6
MGDG	24	8
PA	11	10
PC	20	12
PE	31	30
PG	22	17
PI	20	11
PMeOH	5	3
PS	7	3
SPH	2	1
SQDG	26	12
TG	202	194

Table S6. Lipid abundance profiles in SL-EXO and RP-EXO

Compounds	Molecular Weight	Ionization model	Formula	SL-EXO	RP-EXO	CAS
ADGGA((O-16:0)18:2_18:2)	1030.76843	[M+NH4]+	$C_{61}H_{106}O_{12}$	N/A	N/A	-
ADGGA((O-16:0)16:0_18:3)	1004.75278	[M+NH4]+	$C_{59}H_{104}O_{12}$	N/A	N/A	-
ADGGA((O-16:0)18:0_18:2)	1034.79973	[M+NH4]+	$C_{61}H_{110}O_{12}$	N/A	N/A	-
Cer(d18:1/26:0)	677.668594	[M+H]+	$C_{44}H_{87}NO_3$	0.0309774837	0.0299030184	-
Cer(d18:2/24:1)	645.605994	[M+H]+	$C_{42}H_{79}NO_3$	2.49424257	4.53563929	-
Cer(d18:2/22:1)	617.574694	[M+H]+	$C_{40}H_{75}NO_3$	0.593404669	1.25978677	-
Cer(d18:2/20:1)	589.543394	[M+H]+	$C_{38}H_{71}NO_3$	0.375643483	0.277055221	-
Cer(d18:1/18:0)	565.543394	[M+H]+	$C_{36}H_{71}NO_3$	0.00642204717	0.00668531823	2304-81-6
Cer(d18:0/22:0(2OH))	639.616559	[M+H]+	$C_{40}H_{81}NO_4$	N/A	0.018315641	-
Cer(d18:0/18:0)	567.559044	[M+H]+	$C_{36}H_{73}NO_3$	N/A	0.00416718786	2304-80-5
Cer(d18:1/25:0)	663.652944	[M+H]+	$C_{43}H_{85}NO_3$	0.0120074264	N/A	67492-18-6
Cer(d18:1/24:0)	649.637294	[M+H]+	$C_{42}H_{83}NO_3$	0.176492928	0.0850676667	34435-05-7
Cer(d18:2/16:1)	533.480794	[M+H]+	$C_{34}H_{63}NO_3$	N/A	0.00681510767	-
Cer(d18:2/18:1)	561.512094	[M+H]+	$C_{36}H_{67}NO_3$	0.471002148	0.677149423	-
Cer(d18:1/24:1)	647.621644	[M+H]+	$C_{42}H_{81}NO_3$	0.0895031426	0.114757555	-
Cer(d18:1/22:1)	619.590344	[M+H]+	$C_{40}H_{77}NO_3$	0.0214642287	0.0180581715	-
Cer(d18:1/22:0)	621.605994	[M+H]+	$C_{40}H_{79}NO_3$	0.180563964	0.063965688	27888-44-4
Cer(d18:1/16:0)	537.512094	[M+H]+	$C_{34}H_{67}NO_3$	0.00921381165	0.0472372169	24696-26-2
Cer(d18:0/16:0(2OH))	555.522659	[M+H]+	$C_{34}H_{69}NO_4$	0.00741188028	0.0201174846	190249-36-6
Cer(d18:1/20:1)	591.559044	[M+H]+	$C_{38}H_{73}NO_3$	0.00471234031	N/A	-
Cer(t18:0/24:0)	667.647859	[M+H]+	$C_{42}H_{85}NO_4$	21.8047085	26.2723785	-
Cer(t18:0/22:0)	639.616559	[M+H]+	$C_{40}H_{81}NO_4$	11.8950592	4.80503061	-
Cer(t17:0/24:0)	653.632209	[M+H]+	$C_{41}H_{83}NO_4$	4.20233564	2.93576492	-
Cer(t18:1/27:0(2OH))	723.674074	[M+H]+	$C_{45}H_{89}NO_5$	0.727886979	0.835960615	-
Cer(t18:1/26:0(2OH))	709.658424	[M+H]+	$C_{44}H_{87}NO_5$	20.097485	65.5616787	-
Cer(t18:1/25:0(2OH))	695.642774	[M+H]+	$C_{43}H_{85}NO_5$	2.61642406	24.3417197	-
Cer(t18:1/24:0(2OH))	681.627124	[M+H]+	$C_{42}H_{83}NO_5$	141.959899	149.853304	-
Cer(t18:1/23:0(2OH))	667.611474	[M+H]+	$C_{41}H_{81}NO_5$	3.0199475	3.24753815	-
Cer(t20:0/24:0(2OH))	711.674074	[M+H]+	$C_{44}H_{89}NO_5$	0.116602124	0.170069829	-
Cer(t18:1/20:0(2OH))	625.564524	[M+H]+	$C_{38}H_{75}NO_5$	1.77080816	0.637707302	-
Cer(t18:0/26:0(2OH))	711.674074	[M+H]+	$C_{44}H_{89}NO_5$	1.12867136	10.2828141	-
Cer(t18:0/25:0(2OH))	697.658424	[M+H]+	$C_{43}H_{87}NO_5$	0.196957375	4.2717851	-
Cer(t18:0/24:0(2OH))	683.642774	[M+H]+	$C_{42}H_{85}NO_5$	4.00725995	37.1154316	-
Cer(t18:0/23:0(2OH))	669.627124	[M+H]+	$C_{41}H_{83}NO_5$	0.152520882	0.773579379	-
Cer(t18:0/22:0(2OH))	655.611474	[M+H]+	$C_{40}H_{81}NO_5$	1.92899372	4.23868171	-

Cer(t18:0/26:0)	695.679159	[M+H]+	$C_{44}H_{89}NO_4$	3.81008992	1.9075583	-
Cer(t18:0/20:0(2OH))	627.580174	[M+H]+	$C_{38}H_{77}NO_5$	0.0659959762	0.0836011944	-
Cer(t18:1/26:1(2OH))	707.642774	[M+H]+	$C_{44}H_{85}NO_5$	N/A	0.0634144966	-
Cer(t17:0/26:0)	681.663509	[M+H]+	$C_{43}H_{87}NO_4$	N/A	0.0337102519	-
Cer(t16:0/24:0(2OH))	655.611474	[M+H]+	$C_{40}H_{81}NO_5$	N/A	0.0252632331	-
Cer(t18:1/22:0(2OH))	653.595824	[M+H]+	$C_{40}H_{79}NO_5$	61.2388376	45.4812434	-
Cer(t18:0/21:0(2OH))	641.595824	[M+H]+	$C_{39}H_{79}NO_5$	0.563550118	0.306960385	-
Cer(t18:0/25:1(2OH))	695.642774	[M+H]+	$C_{43}H_{85}NO_5$	N/A	0.582243933	-
Cer(t18:1/26:0)	693.663509	[M+H]+	$C_{44}H_{87}NO_4$	5.1783283	9.169622	-
Cer(t18:0/26:1(2OH))	709.658424	[M+H]+	$C_{44}H_{87}NO_5$	0.0842580111	0.0884054155	-
Cer(t17:0/24:0(2OH))	669.627124	[M+H]+	$C_{41}H_{83}NO_5$	0.0267112333	0.204297383	-
Cer(t18:1/24:0)	665.632209	[M+H]+	$C_{42}H_{83}NO_4$	23.6684014	67.1616792	-
Coenzyme Q9	794.62131	[M+H]+	$C_{54}H_{82}O_4$	1.66604697	1.50697188	303-97-9
DG(16:0_20:0)	624.569275	[M+NH4]+	$C_{39}H_{76}O_5$	0.194687774	0.352991717	-
DG(18:0_18:0)	624.569275	[M+NH4]+	$C_{39}H_{76}O_5$	12.8030916	15.7663481	-
DG(17:0_18:0)	610.553625	[M+NH4]+	$C_{38}H_{74}O_5$	0.0503868979	0.119568275	-
DG(14:0_20:0)	596.537975	[M+NH4]+	$C_{37}H_{72}O_5$	0.329875902	0.279919468	-
DG(16:0_18:0)	596.537975	[M+NH4]+	$C_{37}H_{72}O_5$	14.8011222	17.1018306	-
DG(16:0_18:2)	592.506675	[M+NH4]+	$C_{37}H_{68}O_5$	263.299451	370.512648	51621-26-2
DG(16:0_16:0)	568.506675	[M+NH4]+	$C_{35}H_{68}O_5$	6.81016114	8.14844927	-
DG(18:1_20:0)	650.584925	[M+NH4]+	$C_{41}H_{78}O_5$	0.944674053	0.529160207	-
DG(18:1_22:0)	678.616225	[M+NH4]+	$C_{43}H_{82}O_5$	0.440667105	0.515191282	-
DG(16:0_18:1)	594.522325	[M+NH4]+	$C_{37}H_{70}O_5$	59.7261592	21.9165445	29541-66-0
DG(17:0_18:1)	608.537975	[M+NH4]+	$C_{38}H_{72}O_5$	0.565548669	0.423919718	-
DG(14:0_18:2)	564.475375	[M+NH4]+	$C_{35}H_{64}O_5$	0.794490146	2.4627371	-
DG(15:0_18:2)	578.491025	[M+NH4]+	$C_{36}H_{66}O_5$	0.290322956	0.901828681	-
DG(14:0_22:0)	624.569275	[M+NH4]+	$C_{39}H_{76}O_5$	0.366476909	0.4034428	-
DG(14:0_18:0)	568.506675	[M+NH4]+	$C_{35}H_{68}O_5$	0.160903743	0.195644257	-
DG(16:1_20:4)	614.491025	[M+NH4]+	$C_{39}H_{66}O_5$	0.23428077	0.0269615281	-
DG(18:0_18:2)	620.537975	[M+NH4]+	$C_{39}H_{72}O_5$	60.2082189	65.7485603	-
DG(18:2_18:3)	614.491025	[M+NH4]+	$C_{39}H_{66}O_5$	39.5269629	6.90209019	-
DG(18:2_20:2)	644.537975	[M+NH4]+	$C_{41}H_{72}O_5$	1.13544273	4.34498268	-
DG(18:2_18:2)	616.506675	[M+NH4]+	$C_{39}H_{68}O_5$	105.625539	113.805465	-
DG(18:3_24:0)	702.616225	[M+NH4]+	$C_{45}H_{82}O_5$	0.742858218	0.559694382	-
DG(18:3_23:0)	688.600575	[M+NH4]+	$C_{44}H_{80}O_5$	0.0999380686	0.136146741	-
DG(18:3_22:0)	646.553625	[M+NH4]+	$C_{41}H_{74}O_5$	2.73707491	0.70575496	-
DG(18:2_20:1)	646.553625	[M+NH4]+	$C_{41}H_{74}O_5$	20.6938771	25.128739	-

DG(16:0_20:2)	620.537975	[M+NH4]+	$C_{39}H_{72}O_5$	0.195678527	1.39053242	-
DG(18:0_18:3)	618.522325	[M+NH4]+	$C_{39}H_{70}O_5$	34.7391812	2.74142474	-
DG(16:0_18:3)	590.491025	[M+NH4]+	$C_{37}H_{66}O_5$	83.2336111	18.0131982	-
DG(14:0_18:3)	562.459725	[M+NH4]+	$C_{35}H_{62}O_5$	0.360234749	0.0367163171	-
DG(18:2_25:0)	718.647525	[M+NH4]+	$C_{46}H_{86}O_5$	0.0461644314	0.835553537	-
DG(18:2_24:0)	704.631875	[M+NH4]+	$C_{45}H_{84}O_5$	2.88201652	15.1363501	-
DG(18:2_23:0)	690.616225	[M+NH4]+	$C_{44}H_{82}O_5$	0.388802456	2.96617501	-
DG(18:2_22:0)	676.600575	[M+NH4]+	$C_{43}H_{80}O_5$	4.10450123	12.6145986	-
DG(18:1_18:1)	620.537975	[M+NH4]+	$C_{39}H_{72}O_5$	8.89145295	0.945177316	-
DG(18:1_18:2)	618.522325	[M+NH4]+	$C_{39}H_{70}O_5$	38.1176823	11.5136243	91125-76-7
DG(17:0_18:2)	606.522325	[M+NH4]+	$C_{38}H_{70}O_5$	3.37795379	10.7395745	-
DG(18:0_18:1)	622.553625	[M+NH4]+	$C_{39}H_{74}O_5$	11.736699	3.30903334	-
DG(18:2_18:4)	612.475375	[M+NH4]+	$C_{39}H_{64}O_5$	8.90735092	0.0432240227	-
DG(18:4_18:5)	606.428425	[M+NH4]+	$C_{39}H_{58}O_5$	0.425871827	0.231188905	-
DG(18:2_20:3)	642.522325	[M+NH4]+	$C_{41}H_{70}O_5$	0.2199613	0.174835568	-
DG(18:3_22:1)	672.569275	[M+NH4]+	$C_{43}H_{76}O_5$	0.223396623	0.0943613719	-
DG(18:1_18:3)	616.506675	[M+NH4]+	$C_{39}H_{68}O_5$	18.1230719	N/A	-
DG(18:1_24:0)	706.647525	[M+NH4]+	$C_{45}H_{86}O_5$	0.224677392	0.378641602	-
DG(15:0_18:1)	580.506675	[M+NH4]+	$C_{36}H_{68}O_5$	0.151489882	0.060049225	-
DG(20:0_18:0)	652.600575	[M+NH4]+	$C_{41}H_{80}O_5$	0.124712574	0.0930036173	-
DG(16:0_20:5)	614.491025	[M+NH4]+	$C_{39}H_{66}O_5$	N/A	0.0553774802	-
DG(15:0_20:5)	600.475375	[M+NH4]+	$C_{38}H_{64}O_5$	N/A	0.0207536583	-
DG(16:0_18:4)	588.475375	[M+NH4]+	$C_{37}H_{64}O_5$	0.24942404	N/A	-
DG(18:1_20:4)	642.522325	[M+NH4]+	$C_{41}H_{70}O_5$	0.0176269781	N/A	-
DG(16:2_18:1)	590.491025	[M+NH4]+	$C_{37}H_{66}O_5$	0.0406007925	N/A	-
DG(16:0_20:1)	622.553625	[M+NH4]+	$C_{39}H_{74}O_5$	1.18532268	2.05175158	-
DG(14:0_18:1)	566.491025	[M+NH4]+	$C_{35}H_{66}O_5$	N/A	0.0465808255	-
DG(16:0_19:1)	608.537975	[M+NH4]+	$C_{38}H_{72}O_5$	N/A	0.088803644	-
DG(12:0_20:4)	560.444075	[M+NH4]+	$C_{35}H_{60}O_5$	0.0952604638	N/A	-
DG(18:3_18:3)	612.475375	[M+NH4]+	$C_{39}H_{64}O_5$	70.9253054	0.175981239	-
DG(16:1_18:1)	592.506675	[M+NH4]+	$C_{37}H_{68}O_5$	0.0850288967	N/A	-
DG(16:0_28:2)	732.663175	[M+NH4]+	$C_{47}H_{88}O_5$	0.115566095	N/A	-
DGCC(14:0_16:1)	697.549269	[M+H]+	$C_{40}H_{75}NO_8$	N/A	0.436239431	-
DGDG(18:0_18:3)	942.627975	[M+NH4]+	$C_{51}H_{90}O_{15}$	2074.857	7.0130429	-
DGDG(18:1_18:3)	940.612325	[M+NH4]+	$C_{51}H_{88}O_{15}$	266.889002	3.44689226	-
DGDG(18:2_18:2)	940.612325	[M+NH4]+	$C_{51}H_{88}O_{15}$	568.196802	9.63930064	-
DGDG(17:0_18:3)	928.612325	[M+NH4]+	$C_{50}H_{88}O_{15}$	70.7882398	0.438146288	-

DGDG(18:3_18:3)	936.581025	[M+NH4]+	$C_{51}H_{84}O_{15}$	4182.81378	9.79914291	-
DGDG(18:1_20:5)	964.612325	[M+NH4]+	$C_{53}H_{88}O_{15}$	0.724677355	N/A	-
DGDG(16:0_18:3)	914.596675	[M+NH4]+	$C_{49}H_{86}O_{15}$	4150.20872	31.847219	-
DGDG(18:0_18:2)	944.643625	[M+NH4]+	$C_{51}H_{92}O_{15}$	1390.15064	9.89964625	-
DGDG(16:0_18:2)	916.612325	[M+NH4]+	$C_{49}H_{88}O_{15}$	3257.3658	29.7033734	-
DGDG(16:0_16:0)	892.612325	[M+NH4]+	$C_{47}H_{88}O_{15}$	49.839625	1.04783894	-
DGDG(18:2_18:3)	938.596675	[M+NH4]+	$C_{51}H_{86}O_{15}$	886.115804	4.53250193	-
DGDG(16:2_20:4)	936.581025	[M+NH4]+	$C_{51}H_{84}O_{15}$	50.0790011	N/A	-
DGDG(18:1_18:2)	942.627975	[M+NH4]+	$C_{51}H_{90}O_{15}$	69.9686411	1.77554516	-
DGDG(18:2_20:4)	964.612325	[M+NH4]+	$C_{53}H_{88}O_{15}$	7.66920884	0.343018192	-
DGDG(18:2_16:3)	910.565375	[M+NH4]+	$C_{49}H_{82}O_{15}$	0.107949279	N/A	-
DGDG(14:0_18:2)	888.581025	[M+NH4]+	$C_{47}H_{84}O_{15}$	12.6540697	N/A	-
DGDG(16:0_16:2)	888.581025	[M+NH4]+	$C_{47}H_{84}O_{15}$	2.11077635	N/A	-
DGDG(16:1_18:1)	916.612325	[M+NH4]+	$C_{49}H_{88}O_{15}$	1.65766054	N/A	-
DGDG(14:0_18:3)	886.565375	[M+NH4]+	$C_{47}H_{82}O_{15}$	6.09633647	N/A	-
DGDG(14:0_20:5)	910.565375	[M+NH4]+	$C_{49}H_{82}O_{15}$	0.0501219401	N/A	-
DGGA(16:0_18:2)	768.538765	[M+NH4]+	$C_{43}H_{76}O_{11}$	0.895461949	0.386740878	-
DGTS(18:1_18:3)	759.601304	[M+H]+	$C_{46}H_{81}NO_7$	7.30383875	2.48340111	-
DGTS(16:0_18:1)	737.616954	[M+H]+	$C_{44}H_{83}NO_7$	0.058689998	N/A	-
DGTS(16:0_18:3)	733.585654	[M+H]+	$C_{44}H_{79}NO_7$	0.701885168	N/A	-
DGTS(16:1_20:4)	757.585654	[M+H]+	$C_{46}H_{79}NO_7$	0.246907913	N/A	-
DGTS(18:5_18:5)	747.507404	[M+H]+	$C_{46}H_{69}NO_7$	0.109148473	N/A	-
DGTS(16:0_20:4)	759.601304	[M+H]+	$C_{46}H_{81}NO_7$	4.13443842	1.63151063	-
DGTS(14:0_18:4)	703.538704	[M+H]+	$C_{42}H_{73}NO_7$	0.0885381677	0.103057719	-
DGTS(18:3_18:3)	755.570004	[M+H]+	$C_{46}H_{77}NO_7$	166.608528	4.81974129	-
DGTS(18:3_20:5)	779.570004	[M+H]+	$C_{48}H_{77}NO_7$	71.4205473	4.66209965	-
DGTS(16:1_20:5)	755.570004	[M+H]+	$C_{46}H_{77}NO_7$	1.33251457	1.46493021	-
DGTS(16:0_20:5)	757.585654	[M+H]+	$C_{46}H_{79}NO_7$	69.8508009	25.7773	-
DGTS(18:2_16:3)	729.554354	[M+H]+	$C_{44}H_{75}NO_7$	3.12681091	1.46895822	-
DGTS(18:2_18:2)	759.601304	[M+H]+	$C_{46}H_{81}NO_7$	15.6857854	5.27649655	-
DGTS(18:2_18:4)	755.570004	[M+H]+	$C_{46}H_{77}NO_7$	2.66872041	3.06909544	-
DGTS(18:2_20:5)	781.585654	[M+H]+	$C_{48}H_{79}NO_7$	172.318487	77.8966531	-
FFA(19:1)	296.27153	[M-H]-	$C_{19}H_{36}O_2$	3.57135034	5.02012151	-
FFA(17:1)	268.24023	[M-H]-	$C_{17}H_{32}O_2$	0.143369237	4.20599644	-
FFA(31:0)	466.47498	[M-H]-	$C_{31}H_{62}O_2$	0.17024631	N/A	38232-01-8
FFA(16:1)	254.22458	[M-H]-	$C_{16}H_{30}O_2$	N/A	10.2174712	373-49-9
FFA(20:1)	310.28718	[M-H]-	$C_{20}H_{38}O_2$	N/A	N/A	5561-99-9

FFA(24:1)	366.34978	[M-H]-	$C_{24}H_{46}O_2$	N/A	0.544461932	80434-82-8
FFA(20:4)	304.24023	[M-H]-	$C_{20}H_{32}O_2$	N/A	0.595160585	506-32-1
FFA(10:0)	172.14633	[M-H]-	$C_{10}H_{20}O_2$	0.592771745	1.13378848	334-48-5
FFA(14:0)	228.20893	[M-H]-	$C_{14}H_{28}O_2$	0.644136673	3.95429547	544-63-8
FFA(18:2)	280.24023	[M-H]-	$C_{18}H_{32}O_2$	35.0328981	91.3615626	60-33-3
FFA(22:1)	338.31848	[M-H]-	$C_{22}H_{42}O_2$	0.812187038	10.7003225	112-86-7
FFA(18:1)	282.25588	[M-H]-	$C_{18}H_{34}O_2$	28.4379336	152.411361	112-80-1
FFA(30:0)	452.45933	[M-H]-	$C_{30}H_{60}O_2$	7.57978577	0.262251632	506-50-3
FFA(28:0)	424.42803	[M-H]-	$C_{28}H_{56}O_2$	4.90972799	0.744622192	506-48-9
FFA(22:0)	340.33413	[M-H]-	$C_{22}H_{44}O_2$	0.550951342	5.39678561	112-85-6
FFA(20:3)	306.25588	[M-H]-	$C_{20}H_{34}O_2$	1.65193018	1.27537607	1783-84-2
FFA(18:3)	278.22458	[M-H]-	$C_{18}H_{30}O_2$	69.3886134	5.75293468	463-40-1
FFA(18:0)	284.27153	[M-H]-	$C_{18}H_{36}O_2$	26.7198606	60.4360449	57-11-4
FFA(17:0)	270.25588	[M-H]-	$C_{17}H_{34}O_2$	0.703317539	9.01168297	506-12-7
FFA(16:0)	256.24023	[M-H]-	$C_{16}H_{32}O_2$	52.0841063	330.403898	57-10-3
FFA(15:0)	242.22458	[M-H]-	$C_{15}H_{30}O_2$	0.234046105	3.30179822	1002-84-2
FFA(20:2)	308.27153	[M-H]-	$C_{20}H_{36}O_2$	0.739732933	17.5463213	5598-38-9
HexCer(t18:1/22:0(2OH))	815.648649	[M+H]+	$C_{46}H_{89}NO_{10}$	25.333029	17.7295077	-
HexCer(t18:1/23:0(2OH))	829.664299	[M+H]+	$C_{47}H_{91}NO_{10}$	1.55379012	1.31340546	-
HexCer(t18:1/27:0(2OH))	885.726899	[M+H]+	$C_{51}H_{99}NO_{10}$	0.399812199	0.480191428	-
HexCer(t18:1/24:0(2OH))	843.679949	[M+H]+	$C_{48}H_{93}NO_{10}$	54.1333192	45.9249501	-
HexCer(d16:2/26:0(2OH))	981.660004	[M+H]+	$C_{48}H_{91}NO_9$	N/A	0.0129339275	-
HexCer(t18:1/26:0(2OH))	871.711249	[M+H]+	$C_{50}H_{97}NO_{10}$	34.9817172	38.7084411	-
HexCer(d18:1/24:1)	809.674469	[M+H]+	$C_{48}H_{91}NO_8$	0.302344921	0.659558242	-
HexCer(d16:1/18:0)	699.564919	[M+H]+	$C_{40}H_{77}NO_8$	0.0832028611	0.129728589	-
HexCer(d18:2/20:0)	753.611869	[M+H]+	$C_{44}H_{83}NO_8$	0.14881097	0.120527319	-
HexCer(d18:2/22:0)	781.643169	[M+H]+	$C_{46}H_{87}NO_8$	0.294821626	0.687438372	-
HexCer(d18:2/24:0)	809.674469	[M+H]+	$C_{48}H_{91}NO_8$	1.15931488	2.21747592	-
HexCer(d18:2/24:1)	807.658819	[M+H]+	$C_{48}H_{89}NO_8$	16.1354165	34.0558252	-
HexCer(t18:1/26:1(2OH))	869.695599	[M+H]+	$C_{50}H_{95}NO_{10}$	0.24062709	0.089611402	-
HexCer(d18:1/20:1)	753.611869	[M+H]+	$C_{44}H_{83}NO_8$	0.0296032411	0.0211253309	-
HexCer(t18:1/28:0(2OH))	899.742549	[M+H]+	$C_{52}H_{101}NO_{10}$	1.82538944	1.10409837	-
LDGTS(20:0)	529.434239	[M+H]+	$C_{30}H_{59}NO_6$	0.139441259	N/A	-
LDGTS(16:2)	469.340339	[M+H]+	$C_{26}H_{47}NO_6$	N/A	0.16226938	-
LDGTS(18:2)	497.371639	[M+H]+	$C_{28}H_{51}NO_6$	0.701078184	0.170842235	-
LDGTS(18:0)	501.402939	[M+H]+	$C_{28}H_{55}NO_6$	0.0549641275	0.0933079154	-
LDGTS(18:3)	495.355989	[M+H]+	$C_{28}H_{49}NO_6$	0.466492023	N/A	-

LPA(16:3)	404.196392	[M-H]-	$C_{19}H_{33}O_7P$	0.123667148	N/A	-
LPA(18:3)	432.227692	[M-H]-	$C_{21}H_{37}O_7P$	8.85232238	N/A	-
LPA(18:0)	438.27464	[M-H]-	$C_{21}H_{43}O_7P$	2.75318103	1.09699867	-
LPA(16:0)	410.24334	[M-H]-	$C_{19}H_{39}O_7P$	2.631458	1.37794023	-
LPA(18:1)	436.258993	[M-H]-	$C_{21}H_{41}O_7P$	N/A	0.303820368	-
LPA(18:2)	434.243343	[M-H]-	$C_{21}H_{39}O_7P$	N/A	9.19557981	-
LPA(22:6)	482.243343	[M-H]-	$C_{25}H_{39}O_7P$	108.605151	N/A	-
LPC(12:0)	439.269891	[M+H]+	$C_{20}H_{42}NO_7P$	0.0367301163	0.0284486114	-
LPC(15:1)	479.301191	[M+H]+	$C_{23}H_{46}NO_7P$	0.0887279747	0.0771279164	-
LPC(16:1)	493.316841	[M+H]+	$C_{24}H_{48}NO_7P$	N/A	1.9339497	76790-27-7
LPC(20:1)	549.379441	[M+H]+	$C_{28}H_{56}NO_7P$	23.8929431	18.0439977	-
LPC(20:2)	547.363791	[M+H]+	$C_{28}H_{54}NO_7P$	1.38458753	2.99273994	-
LPC(20:4)	543.332491	[M+H]+	$C_{28}H_{50}NO_7P$	N/A	0.309741496	60701-99-7
LPC(24:1)	605.442041	[M+H]+	$C_{32}H_{64}NO_7P$	0.0977946825	N/A	-
LPC(17:0)	509.348141	[M+H]+	$C_{25}H_{52}NO_7P$	3.25620232	3.83666303	50930-23-9
LPC(22:6)	567.332491	[M+H]+	$C_{30}H_{50}NO_7P$	0.0603303197	0.0368894872	162440-05-3
LPC(20:3)	545.348141	[M+H]+	$C_{28}H_{52}NO_7P$	0.263774606	0.308515372	1199257-41-4
LPC(18:3)	517.316841	[M+H]+	$C_{26}H_{48}NO_7P$	255.722502	14.96442	-
LPC(18:2)	519.332491	[M+H]+	$C_{26}H_{50}NO_7P$	729.45072	247.160639	-
LPC(18:1)	521.348141	[M+H]+	$C_{26}H_{52}NO_7P$	309.667069	22.3519645	-
LPC(24:0)	607.457691	[M+H]+	$C_{32}H_{66}NO_7P$	2.89991002	16.1130279	-
LPC(18:0)	523.363791	[M+H]+	$C_{26}H_{54}NO_7P$	60.2391468	34.7654064	-
LPC(22:5)	569.348141	[M+H]+	$C_{30}H_{52}NO_7P$	N/A	0.0150759319	-
LPC(22:4)	571.363791	[M+H]+	$C_{30}H_{54}NO_7P$	0.0533555164	0.0227823242	-
LPC(15:0)	481.316841	[M+H]+	$C_{23}H_{48}NO_7P$	0.91996052	0.553037931	-
LPC(14:0)	467.301191	[M+H]+	$C_{22}H_{46}NO_7P$	1.34590034	0.512830903	20559-16-4
LPC(20:0)	551.395091	[M+H]+	$C_{28}H_{58}NO_7P$	2.08447776	N/A	108341-80-6
LPC(22:0)	579.426391	[M+H]+	$C_{30}H_{62}NO_7P$	0.37374556	N/A	-
LPC(14:1)	465.285541	[M+H]+	$C_{22}H_{44}NO_7P$	N/A	0.0758777146	-
LPC(22:1)	577.410741	[M+H]+	$C_{30}H_{60}NO_7P$	N/A	N/A	-
LPC(22:2)	575.395091	[M+H]+	$C_{30}H_{58}NO_7P$	0.750544122	0.338496791	-
LPC(16:0)	495.332491	[M+H]+	$C_{24}H_{50}NO_7P$	356.521055	120.407173	-
LPE(20:1)	507.332491	[M+H]+	$C_{25}H_{50}NO_7P$	5.45485082	6.62579663	-
LPE(18:1)	479.301191	[M+H]+	$C_{23}H_{46}NO_7P$	11.1089911	4.30232413	-
LPE(20:2)	505.316841	[M+H]+	$C_{25}H_{48}NO_7P$	0.0922017221	0.703071159	-
LPE(18:3)	475.269891	[M+H]+	$C_{23}H_{42}NO_7P$	16.9393512	1.37749324	-
LPE(18:0)	481.316841	[M+H]+	$C_{23}H_{48}NO_7P$	8.72166767	10.9440434	-

LPE(18:2)	477.285541	[M+H]+	$C_{23}H_{44}NO_7P$	86.2762846	57.7669914	-
LPE(17:0)	467.301191	[M+H]+	$C_{22}H_{46}NO_7P$	N/A	0.649135734	-
LPE(16:1)	451.269891	[M+H]+	$C_{21}H_{42}NO_7P$	N/A	1.00094756	-
LPE(16:0)	453.285541	[M+H]+	$C_{21}H_{44}NO_7P$	49.6810677	82.498844	-
LPG(18:0)	512.311422	[M-H]-	$C_{24}H_{49}O_9P$	0.231056198	0.0855562548	-
LPG(20:0)	540.342722	[M-H]-	$C_{26}H_{53}O_9P$	0.224166092	0.531873613	-
LPG(18:1)	510.295772	[M-H]-	$C_{24}H_{47}O_9P$	1.42346396	0.25228471	-
LPG(18:2)	508.280122	[M-H]-	$C_{24}H_{45}O_9P$	12.0761013	3.24953225	-
LPG(16:0)	484.280122	[M-H]-	$C_{22}H_{45}O_9P$	N/A	1.14886865	-
LPG(16:1)	482.264472	[M-H]-	$C_{22}H_{43}O_9P$	4.80157651	N/A	-
LPI(18:3)	596.296167	[M-H]-	$C_{27}H_{49}O_{12}P$	0.0564100244	N/A	-
LPI(16:1)	570.280517	[M-H]-	$C_{25}H_{47}O_{12}P$	N/A	0.2226721	-
LPI(18:1)	598.311817	[M-H]-	$C_{27}H_{51}O_{12}P$	0.370463242	0.519413238	-
LPI(18:2)	596.296167	[M-H]-	$C_{27}H_{49}O_{12}P$	N/A	8.96154535	-
LPI(16:0)	572.296167	[M-H]-	$C_{25}H_{49}O_{12}P$	17.0621829	5.74984937	-
LPS(22:0)	581.369271	[M-H]-	$C_{28}H_{56}NO_9P$	N/A	0.342769259	-
LPS(16:1)	495.259721	[M-H]-	$C_{22}H_{42}NO_9P$	0.500543135	3.86013223	-
LPS(18:2)	521.275371	[M-H]-	$C_{24}H_{44}NO_9P$	N/A	2.6168225	-
MG(15:0)	316.26136	[M+NH4]+	$C_{18}H_{36}O_4$	N/A	0.257313969	-
MG(18:3)	354.27701	[M+NH4]+	$C_{21}H_{36}O_4$	44.751353	8.48990385	-
MG(18:2)	354.27701	[M+NH4]+	$C_{21}H_{38}O_4$	18.0838463	58.594783	67968-46-1
MG(18:1)	356.29266	[M+NH4]+	$C_{21}H_{40}O_4$	6.80293149	5.93979949	-
MG(18:0)	358.30831	[M+NH4]+	$C_{21}H_{42}O_4$	69.0698229	28.7499598	22610-61-3
MG(16:0)	330.27701	[M+NH4]+	$C_{19}H_{38}O_4$	60.4257168	27.1530442	32899-41-5
MGDG(16:0_18:3)	752.54385	[M+NH4]+	$C_{43}H_{76}O_{10}$	2166.32828	4.16813363	-
MGDG(18:2_18:3)	776.54385	[M+NH4]+	$C_{45}H_{76}O_{10}$	7014.67848	10.7385778	-
MGDG(18:2_18:2)	778.5595	[M+NH4]+	$C_{45}H_{78}O_{10}$	3976.68909	12.1819562	-
MGDG(16:0_18:2)	754.5595	[M+NH4]+	$C_{43}H_{78}O_{10}$	599.563928	7.85018783	-
MGDG(18:2_18:4)	774.5282	[M+NH4]+	$C_{45}H_{74}O_{10}$	5771.99109	24.3783031	-
MGDG(18:2_16:4)	746.4969	[M+NH4]+	$C_{43}H_{70}O_{10}$	1.4829483	N/A	-
MGDG(18:1_20:5)	802.5595	[M+NH4]+	$C_{47}H_{78}O_{10}$	0.636945028	N/A	-
MGDG(16:3_18:4)	744.48125	[M+NH4]+	$C_{43}H_{68}O_{10}$	3.41858821	4.31062193	-
MGDG(18:3_18:3)	774.5282	[M+NH4]+	$C_{45}H_{74}O_{10}$	N/A	N/A	-
MGDG(18:1_18:4)	776.54385	[M+NH4]+	$C_{45}H_{76}O_{10}$	361.106899	N/A	-
MGDG(16:1_20:4)	776.54385	[M+NH4]+	$C_{45}H_{76}O_{10}$	33.3048337	N/A	-
MGDG(16:1_16:4)	720.48125	[M+NH4]+	$C_{41}H_{68}O_{10}$	0.383818734	N/A	-
MGDG(16:0_20:4)	778.5595	[M+NH4]+	$C_{45}H_{78}O_{10}$	4.89831981	N/A	-

MGDG(17:1_18:3)	764.54385	[M+NH4]+	$C_{44}H_{76}O_{10}$	26.135993	N/A	-
MGDG(16:3_18:3)	746.4969	[M+NH4]+	$C_{43}H_{70}O_{10}$	18.8677728	N/A	-
MGDG(18:1_16:3)	750.5282	[M+NH4]+	$C_{43}H_{74}O_{10}$	1.22399403	N/A	-
MGDG(18:0_18:2)	782.5908	[M+NH4]+	$C_{45}H_{82}O_{10}$	12.1619418	N/A	-
MGDG(16:0_16:2)	726.5282	[M+NH4]+	$C_{41}H_{74}O_{10}$	1.23029811	N/A	-
MGDG(16:0_16:0)	730.5595	[M+NH4]+	$C_{41}H_{78}O_{10}$	1.99706685	N/A	-
MGDG(16:0_16:1)	728.54385	[M+NH4]+	$C_{41}H_{76}O_{10}$	2.24819183	1.71387566	-
MGDG(16:0_16:3)	724.51255	[M+NH4]+	$C_{41}H_{72}O_{10}$	10.2078908	6.9258642	-
MGDG(16:1_18:3)	750.5282	[M+NH4]+	$C_{43}H_{74}O_{10}$	96.7540996	N/A	-
MGDG(16:2_18:2)	750.5282	[M+NH4]+	$C_{43}H_{74}O_{10}$	10.2011456	N/A	-
MGDG(14:0_18:2)	726.5282	[M+NH4]+	$C_{41}H_{74}O_{10}$	3.54709416	N/A	-
MGDG(18:4_20:5)	796.51255	[M+NH4]+	$C_{47}H_{72}O_{10}$	6.01132022	N/A	-
PA(16:0_18:1)	674.488657	[M-H]-	$C_{37}H_{71}O_8P$	137.749921	5.55415843	-
PA(16:0_18:2)	672.473007	[M-H]-	$C_{37}H_{69}O_8P$	505.443302	128.215373	-
PA(18:1_20:0)	730.551257	[M-H]-	$C_{41}H_{79}O_8P$	5.21740402	N/A	-
PA(18:1_22:0)	758.582557	[M-H]-	$C_{43}H_{83}O_8P$	2.63493337	N/A	-
PA(16:1_16:1)	644.441707	[M-H]-	$C_{35}H_{65}O_8P$	N/A	0.459065918	-
PA(18:3_22:0)	754.551257	[M-H]-	$C_{43}H_{79}O_8P$	N/A	3.70385931	-
PA(16:1_18:3)	668.441707	[M-H]-	$C_{37}H_{65}O_8P$	2.23582507	N/A	-
PA(18:2_18:2)	696.473007	[M-H]-	$C_{39}H_{69}O_8P$	689.236615	348.851929	-
PA(18:1_22:6)	746.488657	[M-H]-	$C_{43}H_{71}O_8P$	1306.11493	49.5865273	-
PA(16:0_14:0)	620.441707	[M-H]-	$C_{33}H_{65}O_8P$	N/A	N/A	-
PA(18:1_18:3)	696.473007	[M-H]-	$C_{39}H_{69}O_8P$	116.340535	5.86823068	-
PA(18:3_16:0)	670.457357	[M-H]-	$C_{37}H_{67}O_8P$	233.369829	10.2022502	-
PA(18:2_20:0)	728.535607	[M-H]-	$C_{41}H_{77}O_8P$	178.858156	1.27187331	-
PA(18:2_18:3)	694.457357	[M-H]-	$C_{39}H_{67}O_8P$	539.357366	17.9278643	-
PC(18:1_18:2)	783.577806	[M+H]+	$C_{44}H_{82}NO_8P$	47.461281	52.6737573	-
PC(18:1_24:1)	869.687356	[M+H]+	$C_{50}H_{96}NO_8P$	2.41447429	5.19859711	-
PC(18:1_22:1)	841.656056	[M+H]+	$C_{48}H_{92}NO_8P$	2.04370997	3.7261914	-
PC(16:0_18:1)	759.577806	[M+H]+	$C_{42}H_{82}NO_8P$	159.823911	16.4334347	26853-31-6
PC(18:3_18:1)	781.562156	[M+H]+	$C_{44}H_{80}NO_8P$	47.3456712	N/A	-
PC(16:1_18:3)	753.530856	[M+H]+	$C_{42}H_{76}NO_8P$	1.65643056	N/A	-
PC(16:1_18:2)	755.546506	[M+H]+	$C_{42}H_{78}NO_8P$	6.99888036	N/A	182820-31-1
PC(18:1_20:1)	813.624756	[M+H]+	$C_{46}H_{88}NO_8P$	0.328328333	N/A	-
PC(16:1_18:1)	757.562156	[M+H]+	$C_{42}H_{80}NO_8P$	2.08703757	N/A	-
PC(24:0_18:1)	871.703006	[M+H]+	$C_{50}H_{98}NO_8P$	0.514037015	N/A	-
PC(20:4_20:5)	827.546506	[M+H]+	$C_{48}H_{78}NO_8P$	1.05894481	N/A	-

PC(14:0_18:1)	731.546506	[M+H]+	$C_{40}H_{78}NO_8P$	51.933025	227.691466	-
PC(16:3_18:3)	749.499556	[M+H]+	$C_{42}H_{72}NO_8P$	4.77052265	N/A	-
PC(18:2_16:3)	751.515206	[M+H]+	$C_{42}H_{74}NO_8P$	N/A	14.6870239	-
PC(18:2_18:2)	781.562156	[M+H]+	$C_{44}H_{80}NO_8P$	138.471779	159.800591	998-06-1
PC(16:0_18:2)	757.562156	[M+H]+	$C_{42}H_{80}NO_8P$	232.498818	478.866342	17708-90-6
PC(18:1_18:1)	785.593456	[M+H]+	$C_{44}H_{84}NO_8P$	13.0633735	7.74285614	68737-67-7
PC(18:0_18:2)	785.593456	[M+H]+	$C_{44}H_{84}NO_8P$	100.698449	64.3018296	27098-24-4
PC(16:0_18:3)	755.546506	[M+H]+	$C_{42}H_{78}NO_8P$	126.826456	34.6597018	203393-39-9
PC(18:0_18:1)	787.609106	[M+H]+	$C_{44}H_{86}NO_8P$	0.990244685	N/A	-
PC(18:2_18:3)	779.546506	[M+H]+	$C_{44}H_{78}NO_8P$	111.459567	23.0405491	85046-19-1
PE(18:3_20:1)	767.546506	[M-H]-	$C_{43}H_{78}NO_8P$	41.0279991	15.0979474	-
PE(18:0_18:0)	747.577806	[M-H]-	$C_{41}H_{82}NO_8P$	0.476361244	N/A	1069-79-0
PE(18:3_18:1)	739.515206	[M-H]-	$C_{41}H_{74}NO_8P$	23.9429135	3.50892288	-
PE(16:1_17:1)	701.499556	[M-H]-	$C_{38}H_{72}NO_8P$	N/A	1.31376539	-
PE(16:0_16:3)	685.468256	[M-H]-	$C_{37}H_{68}NO_8P$	0.103947869	N/A	-
PE(16:1_20:4)	737.499556	[M-H]-	$C_{41}H_{72}NO_8P$	N/A	0.610137549	-
PE(18:0_14:0)	691.515206	[M-H]-	$C_{37}H_{74}NO_8P$	0.0557676163	N/A	-
PE(18:3_16:1)	711.483906	[M-H]-	$C_{39}H_{70}NO_8P$	5.54235903	3.89955706	-
PE(16:1_18:0)	717.530856	[M-H]-	$C_{39}H_{76}NO_8P$	0.130452777	0.690302102	-
PE(14:0_18:3)	685.468256	[M-H]-	$C_{37}H_{68}NO_8P$	4.72134225	0.50660109	-
PE(18:2_16:0)	715.515206	[M-H]-	$C_{39}H_{74}NO_8P$	708.014681	2094.2795	-
PE(18:2_22:0)	799.609106	[M-H]-	$C_{45}H_{86}NO_8P$	121.134347	435.897001	-
PE(18:3_16:0)	713.499556	[M-H]-	$C_{39}H_{72}NO_8P$	308.998533	96.579107	-
PE(17:1_18:2)	727.515206	[M-H]-	$C_{40}H_{74}NO_8P$	12.4872966	8.43102653	-
PE(18:1_18:2)	741.530856	[M-H]-	$C_{41}H_{76}NO_8P$	45.9257428	76.4338927	1188-56-3
PE(18:2_14:0)	687.483906	[M-H]-	$C_{37}H_{70}NO_8P$	13.701142	137.340097	-
PE(16:1_18:2)	713.499556	[M-H]-	$C_{39}H_{72}NO_8P$	9.00581853	179.79362	-
PE(16:0_18:1)	717.530856	[M-H]-	$C_{39}H_{76}NO_8P$	226.489262	54.7364135	-
PE(16:1_16:0)	689.499556	[M-H]-	$C_{37}H_{72}NO_8P$	8.71243705	144.34545	-
PE(16:0_16:0)	691.515206	[M-H]-	$C_{37}H_{74}NO_8P$	0.945531567	3.44174898	923-61-5
PE(16:0_14:0)	663.483906	[M-H]-	$C_{35}H_{70}NO_8P$	0.381831591	1.87007632	-
PE(18:0_18:3)	741.530856	[M-H]-	$C_{41}H_{76}NO_8P$	63.9213337	10.8986775	-
PE(18:2_18:2)	739.515206	[M-H]-	$C_{41}H_{74}NO_8P$	603.716273	1654.27159	20707-71-5
PE(16:2_18:3)	709.468256	[M-H]-	$C_{39}H_{68}NO_8P$	1.08852009	0.25798609	-
PE(18:2_18:3)	737.499556	[M-H]-	$C_{41}H_{72}NO_8P$	157.550905	44.2186359	-
PE(16:3_18:3)	707.452606	[M-H]-	$C_{39}H_{66}NO_8P$	1.3225328	0.0894369031	-
PE(14:0_18:1)	689.499556	[M-H]-	$C_{37}H_{72}NO_8P$	1.03553353	1.33799536	-

PE(20:1_18:0)	773.593456	[M-H]-	$C_{43}H_{84}NO_{8}P$	0.803518812	1.70586509	-
PE(16:0_16:2)	687.483906	[M-H]-	$C_{37}H_{70}NO_{8}P$	0.453182955	2.48791496	-
PE(17:0_18:2)	729.530856	[M-H]-	$C_{40}H_{76}NO_{8}P$	10.0463264	34.7992911	-
PE(20:1_18:1)	771.577806	[M-H]-	$C_{43}H_{82}NO_{8}P$	9.63392575	3.30902189	-
PE(18:1_16:1)	715.515206	[M-H]-	$C39H_{74}NO_{8}P$	1.92416028	46.0482922	-
PE(16:1_16:1)	687.483906	[M-H]-	$C_{37}H_{7}0NO_{8}P$	0.103337446	51.0245177	-
PG(14:0_22:5)	740.462837	[M-H]-	$C40H_{6}9O_{1}0P$	0.0275864827	N/A	-
PG(16:2_18:3)	740.462837	[M-H]-	$C40H_{6}9O_{10}P$	0.0504868574	N/A	-
PG(18:1_20:2)	800.556737	[M-H]-	$C_{44}H_{81}O_{10}P$	N/A	0.316606467	-
PG(16:1_16:1)	718.478487	[M-H]-	$C_{38}H_{71}O_{10}P$	N/A	0.0589221744	-
PG(18:0_18:0)	778.572387	[M-H]-	$C_{42}H_{83}O_{10}P$	0.154114437	N/A	-
PG(18:2_22:6)	818.509787	[M-H]-	$C_{46}H_{75}O_{10}P$	0.0246045798	N/A	-
PG(18:3_18:4)	764.462837	[M-H]-	$C_{42}H_{6}9O_{10}P$	33.94461	0.175941989	-
PG(18:2_18:3)	768.494137	[M-H]-	$C_{42}H_{73}O_{10}P$	1.23485879	1.24667115	-
PG(18:2_18:1)	772.525437	[M-H]-	$C_{42}H_{77}O_{10}P$	19.2202897	0.705504845	-
PG(18:0_18:1)	776.556737	[M-H]-	$C_{42}H_{81}O_{10}P$	1.83772474	1.63860971	-
PG(18:2_16:0)	746.509787	[M-H]-	$C40H_{75}O_{10}P$	1030.04156	105.214625	-
PG(18:1_18:1)	774.541087	[M-H]-	$C_{42}H_{7}9O_{10}P$	1.74796665	0.81875089	-
PG(14:0_18:3)	716.462837	[M-H]-	$C_{38}H_{6}9O_{10}P$	3.8780516	0.0613677577	-
PG(18:3_16:0)	744.494137	[M-H]-	$C40H_{73}O_{10}P$	454.26847	5.24427997	-
PG(16:0_18:1)	748.525437	[M-H]-	$C40H_{77}O_{10}P$	688.231001	5.7735491	-
PG(18:2_18:2)	770.509787	[M-H]-	$C_{42}H_{75}O_{10}P$	49.4196938	5.12934058	-
PG(16:0_20:4)	770.509787	[M-H]-	$C_{42}H_{75}O_{10}P$	0.211020503	0.0487880398	-
PG(16:0_16:1)	720.494137	[M-H]-	$C_{38}H_{73}O_{10}P$	11.8351437	6.56669002	-
PG(18:0_18:2)	774.541087	[M-H]-	$C_{42}H_{7}9O_{10}P$	1.52784366	N/A	-
PG(15:0_16:0)	708.494137	[M-H]-	$C_{37}H_{73}O_{10}P$	0.709274828	N/A	-
PG(18:3_18:3)	766.478487	[M-H]-	$C_{42}H_{71}O_{10}P$	N/A	0.0123373798	-
PG(16:1_18:3)	742.478487	[M-H]-	$C40H_{71}O_{10}P$	551.149998	N/A	-
PG(20:1_18:1)	802.572387	[M-H]-	$C_{44}H_{83}O_{10}P$	10.6640387	1.47148993	-
PG(18:1_14:0)	720.494137	[M-H]-	$C_{38}H_{73}O_{10}P$	1.08732436	0.198236778	-
PG(18:1_16:1)	746.509787	[M-H]-	$C40H_{75}O_{10}P$	118.311792	N/A	-
PI(16:0_16:1)	808.510182	[M-H]-	$C_{41}H_{77}O_{13}P$	11.4972828	7.43071999	-
PI(18:2_20:4)	882.525832	[M-H]-	$C_{47}H_{7}9O_{13}P$	0.224678771	N/A	-
PI(18:1_20:1)	890.588432	[M-H]-	$C_{47}H_{87}O_{13}P$	0.739414194	N/A	-
PI(16:1_17:1)	820.510182	[M-H]-	$C_{42}H_{77}O_{13}P$	1.60783191	N/A	-
PI(18:1_20:2)	888.572782	[M-H]-	$C_{47}H_{85}O_{13}P$	0.13478579	N/A	-
PI(18:1_19:2)	874.557132	[M-H]-	$C_{46}H_{83}O_{13}P$	0.0841732457	N/A	-

PI(16:0_20:3)	860.541482	[M-H]-	$C_{45}H_{81}O_{13}P$	0.51838079	N/A	-
PI(17:0_18:1)	850.557132	[M-H]-	$C_{44}H_{83}O_{13}P$	2.96463326	N/A	-
PI(20:2_18:3)	884.541482	[M-H]-	$C_{47}H_{81}O_{13}P$	0.704299443	N/A	-
PI(16:0_18:1)	836.541482	[M-H]-	$C_{43}H_{81}O_{13}P$	914.962657	44.9926828	-
PI(20:3_20:5)	906.525832	[M-H]-	$C_{49}H_{79}O_{13}P$	0.121441312	N/A	-
PI(18:2_16:0)	834.525832	[M-H]-	$C_{43}H_{79}O_{13}P$	3813.0059	657.401653	-
PI(18:1_18:1)	862.557132	[M-H]-	$C_{45}H_{83}O_{13}P$	20.9823015	0.920482987	-
PI(18:3_16:0)	832.510182	[M-H]-	$C_{43}H_{77}O_{13}P$	2920.48306	26.6483655	-
PI(18:1_18:2)	860.541482	[M-H]-	$C_{45}H_{81}O_{13}P$	126.15513	10.4626221	-
PI(18:2_18:2)	858.525832	[M-H]-	$C_{45}H_{79}O_{13}P$	223.391686	17.4815094	182509-36-0
PI(18:2_18:3)	856.510182	[M-H]-	$C_{45}H_{77}O_{13}P$	221.025564	3.13822684	-
PI(18:3_18:3)	854.494532	[M-H]-	$C_{45}H_{75}O_{13}P$	35.5000607	0.16235066	-
PI(16:0_16:0)	810.525832	[M-H]-	$C_{41}H_{79}O_{13}P$	33.2491844	8.18861998	-
PI(16:0_18:0)	838.557132	[M-H]-	$C_{43}H_{83}O_{13}P$	69.1415091	2.76658259	182509-29-1
PMeOH(14:0_20:5)	680.441707	[M-H]-	$C_{38}H_{65}O_{8}P$	0.035649041	N/A	-
PMeOH(16:0_20:5)	708.473007	[M-H]-	$C_{40}H_{69}O_{8}P$	0.0681636699	N/A	-
PMeOH(18:2_18:2)	710.488657	[M-H]-	$C_{40}H_{71}O_{8}P$	4.38940654	9.42061302	-
PMeOH(16:0_18:3)	684.473007	[M-H]-	$C_{38}H_{69}O_{8}P$	3.64365906	1.41826707	-
PMeOH(16:0_18:2)	686.488657	[M-H]-	$C_{38}H_{71}O_{8}P$	19.0892045	18.5732141	-
PS(16:0_20:5)	781.489386	[M-H]-	$C_{42}H_{72}NO_{10}P$	23.2217799	N/A	-
PS(18:1_19:0)	803.567636	[M-H]-	$C_{43}H_{82}NO_{10}P$	161.661666	18.2083149	-
PS(18:0_17:1)	775.536336	[M-H]-	$C_{41}H_{78}NO_{10}P$	232.006743	22.9622042	-
PS(16:0_16:1)	733.489386	[M-H]-	$C_{38}H_{72}NO_{10}P$	N/A	4.91746018	-
PS(16:0_18:1)	761.520686	[M-H]-	$C_{40}H_{76}NO_{10}P$	11.5740006	N/A	-
PS(16:2_16:0)	731.473736	[M-H]-	$C_{38}H_{70}NO_{10}P$	4.42065493	N/A	-
PS(18:2_16:0)	759.505036	[M-H]-	$C_{40}H_{74}NO_{10}P$	32.0269683	N/A	-
PS(16:2_16:1)	729.458086	[M-H]-	$C_{38}H_{68}NO_{10}P$	1.12178277	N/A	-
SPH(d16:1)	271.251129	[M+H]+	$C_{16}H_{33}NO_{2}$	1.63029731	1.06034437	-
PhytoSph(d18:1)	315.277344	[M+H]+	$C_{18}H_{37}NO_{3}$	0.137058179	N/A	-
SQDG(16:0_16:2)	790.4901	[M+NH4]+	$C_{41}H_{74}O_{12}S$	0.917299975	N/A	-
SQDG(16:0_18:2)	818.5214	[M+NH4]+	$C_{43}H_{78}O_{12}S$	321.708842	1.57933131	-
SQDG(14:0_18:3)	788.47445	[M+NH4]+	$C_{41}H_{72}O_{12}S$	0.746498085	N/A	-
SQDG(16:0_18:4)	814.4901	[M+NH4]+	$C_{43}H_{74}O_{12}S$	0.959946674	N/A	-
SQDG(18:2_18:4)	838.4901	[M+NH4]+	$C_{45}H_{74}O_{12}S$	5.76886095	0.131943766	-
SQDG(18:4_18:4)	834.4588	[M+NH4]+	$C_{45}H_{70}O_{12}S$	1.36409806	N/A	-
SQDG(18:3_18:4)	836.47445	[M+NH4]+	$C_{45}H_{72}O_{12}S$	0.556326881	N/A	-
SQDG(16:1_20:5)	838.4901	[M+NH4]+	$C_{45}H_{74}O_{12}S$	0.367290552	N/A	-

SQDG(16:1_18:2)	816.50575	[M+NH4]+	$C_{43}H_{76}O_{12}S$	5.41525245	N/A	-
SQDG(16:1_18:3)	814.4901	[M+NH4]+	$C_{43}H_{74}O_{12}S$	2.43151703	N/A	-
SQDG(18:1_18:2)	844.53705	[M+NH4]+	$C_{45}H_{80}O_{12}S$	46.952307	N/A	-
SQDG(16:0_17:1)	806.5214	[M+NH4]+	$C_{42}H_{78}O_{12}S$	1.6056972	0.109532694	-
SQDG(18:1_18:1)	846.5527	[M+NH4]+	$C_{45}H_{82}O_{12}S$	2.67333583	N/A	-
SQDG(16:0_18:0)	822.5527	[M+NH4]+	$C_{43}H_{82}O_{12}S$	20.0604201	N/A	-
SQDG(15:0_16:1)	778.4901	[M+NH4]+	$C_{40}H_{74}O_{12}S$	19.679668	N/A	-
SQDG(16:1_18:1)	818.5214	[M+NH4]+	$C_{43}H_{78}O_{12}S$	1.92942774	N/A	-
SQDG(16:0_20:5)	840.50575	[M+NH4]+	$C_{45}H_{76}O_{12}S$	0.527469608	N/A	-
SQDG(16:0_16:0)	794.5214	[M+NH4]+	$C_{41}H_{78}O_{12}S$	134.875722	0.425558092	-
SQDG(16:0_16:1)	792.50575	[M+NH4]+	$C_{41}H_{76}O_{12}S$	7.66589923	0.260867943	-
SQDG(16:0_18:1)	820.53705	[M+NH4]+	$C_{43}H_{80}O_{12}S$	232.23879	0.603679132	-
SQDG(14:0_18:2)	790.4901	[M+NH4]+	$C_{41}H_{74}O_{12}S$	1.50928494	0.330531736	-
SQDG(16:0_18:3)	816.50575	[M+NH4]+	$C_{43}H_{76}O_{12}S$	303.373886	2.43373121	-
SQDG(18:1_18:3)	842.5214	[M+NH4]+	$C_{45}H_{78}O_{12}S$	42.120319	0.406585938	-
SQDG(18:2_18:2)	842.5214	[M+NH4]+	$C_{45}H_{78}O_{12}S$	69.8898408	0.784989108	-
SQDG(18:2_18:3)	840.50575	[M+NH4]+	$C_{45}H_{76}O_{12}S$	89.0182058	0.254176082	-
SQDG(14:0_16:0)	766.4901	[M+NH4]+	$C_{39}H_{74}O_{12}S$	3.30480933	0.396857559	-
TG(20:1_22:1_18:2)	966.86154	[M+NH4]+	$C_{63}H_{114}O_6$	0.0648028223	0.0409471231	-
TG(18:0_18:0_20:2)	914.83024	[M+NH4]+	$C_{59}H_{110}O_6$	0.0722617761	N/A	-
TG(12:0_16:1_18:1)	774.67374	[M+NH4]+	$C_{49}H_{90}O_6$	N/A	0.0716017734	-
TG(16:0_17:0_18:1)	846.76764	[M+NH4]+	$C_{54}H_{102}O_6$	0.152704284	N/A	-
TG(13:0_16:0_18:1)	790.70504	[M+NH4]+	$C_{50}H_{94}O_6$	N/A	0.0348157623	-
TG(16:0_16:0_23:0)	904.84589	[M+NH4]+	$C_{58}H_{112}O_6$	N/A	0.00974769374	-
TG(12:0_13:0_18:1)	734.64244	[M+NH4]+	$C_{46}H_{86}O_6$	0.0275229796	N/A	-
TG(12:0_14:0_16:1)	720.62679	[M+NH4]+	$C_{45}H_{84}O_6$	N/A	0.0446638987	-
TG(16:0_18:1_24:1)	942.86154	[M+NH4]+	$C_{61}H_{114}O_6$	N/A	0.0363570324	-
TG(15:0_16:0_18:0)	820.75199	[M+NH4]+	$C_{52}H_{100}O_6$	N/A	0.0236745931	-
TG(12:0_16:0_16:1)	748.65809	[M+NH4]+	$C_{47}H_{88}O_6$	N/A	0.21171805	-
TG(10:0_18:1_18:2)	772.65809	[M+NH4]+	$C_{49}H_{88}O_6$	0.0552878115	N/A	-
TG(16:0_17:1_18:3)	840.72069	[M+NH4]+	$C_{54}H_{96}O_6$	0.291537833	N/A	-
TG(18:0_18:2_22:1)	940.84589	[M+NH4]+	$C_{61}H_{112}O_6$	N/A	0.0199642864	-
TG(16:0_16:3_18:1)	826.70504	[M+NH4]+	$C_{53}H_{94}O_6$	0.158031662	N/A	-
TG(16:0_16:0_17:0)	820.75199	[M+NH4]+	$C_{52}H_{100}O_6$	N/A	0.150306599	-
TG(16:0_16:0_20:4)	854.73634	[M+NH4]+	$C_{55}H_{98}O_6$	N/A	0.133529544	-
TG(16:0_18:3_20:1)	882.76764	[M+NH4]+	$C_{57}H_{102}O_6$	2.03044305	N/A	-
TG(18:2_20:0_20:2)	938.83024	[M+NH4]+	$C_{61}H_{110}O_6$	N/A	0.0227215958	-

TG(13:0_15:0_21:5)	810.67374	[M+NH4]+	$C_{52}H_{90}O_6$	0.0304307396	N/A	-
TG(16:0_16:2_18:3)	824.68939	[M+NH4]+	$C_{53}H_{92}O_6$	N/A	0.0806805855	-
TG(16:0_17:1_20:4)	866.73634	[M+NH4]+	$C_{56}H_{98}O_6$	0.0236266156	N/A	-
TG(17:1_17:2_19:2)	866.73634	[M+NH4]+	$C_{56}H_{98}O_6$	0.0251237748	N/A	-
TG(16:0_18:3_20:2)	880.75199	[M+NH4]+	$C_{57}H_{100}O_6$	0.251473373	N/A	-
TG(20:0_18:1_18:2)	884.78329	[M+NH4]+	$C_{57}H_{104}O_6$	0.52969659	N/A	-
TG(15:0_16:0_17:0)	806.73634	[M+NH4]+	$C_{51}H_{98}O_6$	0.0406471607	N/A	-
TG(16:0_16:2_18:2)	826.70504	[M+NH4]+	$C_{53}H_{94}O_6$	12.6721669	17.3667936	-
TG(12:0_14:0_18:0)	750.67374	[M+NH4]+	$C_{47}H_{90}O_6$	N/A	0.0129642914	-
TG(17:0_18:1_18:1)	872.78329	[M+NH4]+	$C_{56}H_{104}O_6$	0.0662634436	0.050358458	-
TG(16:0_16:1_17:1)	816.72069	[M+NH4]+	$C_{52}H_{96}O_6$	2.56172349	2.45025672	-
TG(12:0_16:0_18:2)	774.67374	[M+NH4]+	$C_{49}H_{90}O_6$	0.387117801	0.358459606	-
TG(16:0_20:0_20:1)	916.84589	[M+NH4]+	$C_{59}H_{112}O_6$	0.138218429	0.0347579774	-
TG(18:0_18:1_20:0)	916.84589	[M+NH4]+	$C_{59}H_{112}O_6$	0.204019891	0.0919360465	-
TG(16:0_18:0_20:1)	888.81459	[M+NH4]+	$C_{57}H_{108}O_6$	0.0375045955	0.0118275858	-
TG(17:0_18:0_18:1)	874.79894	[M+NH4]+	$C_{56}H_{106}O_6$	0.0723515451	0.0515582249	-
TG(16:0_16:0_20:1)	860.78329	[M+NH4]+	$C_{55}H_{104}O_6$	0.262169996	0.159248649	-
TG(12:0_16:0_18:1)	776.68939	[M+NH4]+	$C_{49}H_{92}O_6$	0.318710004	0.0846077643	-
TG(15:0_16:0_24:0)	904.84589	[M+NH4]+	$C_{58}H_{112}O_6$	0.0189072644	0.0312574229	-
TG(13:0_14:0_16:0)	736.65809	[M+NH4]+	$C_{46}H_{88}O_6$	0.072344781	N/A	-
TG(16:0_18:1_18:2)	856.75199	[M+NH4]+	$C_{55}H_{100}O_6$	12.7741262	5.70333718	-
TG(16:1_18:1_18:1)	856.75199	[M+NH4]+	$C_{55}H_{100}O_6$	1.133561	1.34154703	-
TG(18:1_18:1_18:1)	884.78329	[M+NH4]+	$C_{57}H_{104}O_6$	0.619016132	0.875431331	122-32-7
TG(18:0_18:1_18:2)	884.78329	[M+NH4]+	$C_{57}H_{104}O_6$	13.8438603	12.2384199	-
TG(18:0_18:0_18:3)	884.78329	[M+NH4]+	$C_{57}H_{104}O_6$	3.62983747	1.35664493	-
TG(16:0_18:3_20:0)	884.78329	[M+NH4]+	$C_{57}H_{104}O_6$	0.78354441	0.0814090344	-
TG(18:1_18:1_20:1)	912.81459	[M+NH4]+	$C_{59}H_{108}O_6$	0.266347815	0.150960404	-
TG(18:0_18:3_20:0)	912.81459	[M+NH4]+	$C_{59}H_{108}O_6$	0.964134264	0.315339315	-
TG(24:0_18:1_18:2)	968.87719	[M+NH4]+	$C_{63}H_{116}O_6$	0.177566704	0.155653325	-
TG(14:0_18:1_18:3)	826.70504	[M+NH4]+	$C_{53}H_{94}O_6$	1.10445536	0.454236697	-
TG(18:2_18:2_22:1)	936.81459	[M+NH4]+	$C_{61}H_{108}O_6$	0.110411661	N/A	-
TG(16:0_18:2_18:2)	854.73634	[M+NH4]+	$C_{55}H_{98}O_6$	83.8925176	79.1524646	-
TG(13:0_15:0_16:0)	750.67374	[M+NH4]+	$C_{47}H_{90}O_6$	0.1508975	N/A	-
TG(12:0_16:3_18:3)	766.61114	[M+NH4]+	$C_{53}H_{90}O_6$	0.0324578148	N/A	-
TG(16:1_18:1_20:5)	876.72069	[M+NH4]+	$C_{57}H_{96}O_6$	N/A	0.160215944	-
TG(17:0_18:3_18:3)	864.72069	[M+NH4]+	$C_{56}H_{96}O_6$	0.148599573	N/A	-
TG(18:1_18:2_18:2)	880.75199	[M+NH4]+	$C_{57}H_{100}O_6$	20.2190769	10.879137	-

TG(18:0_18:2_18:3)	880.75199	[M+NH4]+	$C_{57}H_{100}O_6$	8.36246317	0.664693448	-
TG(18:1_18:1_18:3)	880.75199	[M+NH4]+	$C_{57}H_{100}O_6$	12.3718386	6.57391414	-
TG(18:2_18:3_20:0)	908.78329	[M+NH4]+	$C_{59}H_{104}O_6$	1.42274433	0.414184018	-
TG(18:2_18:3_22:0)	936.81459	[M+NH4]+	$C_{61}H_{108}O_6$	0.572183148	0.153590548	-
TG(16:0_16:1_20:5)	850.70504	[M+NH4]+	$C_{55}H_{94}O_6$	18.1914003	0.629242406	-
TG(16:0_18:2_18:4)	850.70504	[M+NH4]+	$C_{55}H_{94}O_6$	1.57168897	0.139211657	-
TG(16:2_18:1_18:3)	850.70504	[M+NH4]+	$C_{55}H_{94}O_6$	0.442565713	0.839293844	-
TG(18:1_18:2_18:3)	878.73634	[M+NH4]+	$C_{57}H_{98}O_6$	16.5342104	4.86983714	-
TG(18:2_18:2_18:2)	878.73634	[M+NH4]+	$C_{57}H_{98}O_6$	28.2543939	42.805619	-
TG(18:1_18:1_18:4)	878.73634	[M+NH4]+	$C_{57}H_{98}O_6$	7.46801641	2.49635798	-
TG(18:0_18:3_18:3)	878.73634	[M+NH4]+	$C_{57}H_{98}O_6$	6.57681238	0.0612959967	-
TG(18:2_18:3_20:1)	906.76764	[M+NH4]+	$C_{59}H_{102}O_6$	4.45050046	1.74807813	-
TG(16:0_20:3_20:3)	906.76764	[M+NH4]+	$C_{59}H_{102}O_6$	0.0695244525	0.121893915	-
TG(18:2_18:3_22:1)	934.79894	[M+NH4]+	$C_{61}H_{106}O_6$	0.26879731	0.429929876	-
TG(18:2_18:2_18:3)	876.72069	[M+NH4]+	$C_{57}H_{96}O_6$	43.2628105	17.6378762	-
TG(18:1_18:2_18:4)	876.72069	[M+NH4]+	$C_{57}H_{96}O_6$	6.25878547	0.824714347	-
TG(18:2_18:3_18:3)	874.70504	[M+NH4]+	$C_{57}H_{94}O_6$	22.147054	1.31982896	-
TG(16:0_18:3_20:5)	874.70504	[M+NH4]+	$C_{57}H_{94}O_6$	0.915642601	0.279875203	-
TG(18:1_18:3_18:4)	874.70504	[M+NH4]+	$C_{57}H_{94}O_6$	0.425035854	0.0324530352	-
TG(14:0_20:2_22:6)	902.73634	[M+NH4]+	$C_{59}H_{98}O_6$	0.259366582	0.0784626519	-
TG(18:3_18:3_18:3)	872.68939	[M+NH4]+	$C_{57}H_{92}O_6$	6.56916832	0.0455749498	-
TG(17:1_17:3_21:5)	884.68939	[M+NH4]+	$C_{58}H_{92}O_6$	0.187048614	0.307453272	-
TG(17:0_18:2_18:3)	866.73634	[M+NH4]+	$C_{56}H_{98}O_6$	0.569941476	0.206776255	-
TG(16:0_18:1_18:4)	852.72069	[M+NH4]+	$C_{55}H_{96}O_6$	1.4737558	0.250315314	-
TG(16:0_18:2_18:3)	852.72069	[M+NH4]+	$C_{55}H_{96}O_6$	54.7820034	27.4016915	-
TG(15:1_18:2_18:2)	838.70504	[M+NH4]+	$C_{54}H_{94}O_6$	8.41412123	11.2205046	-
TG(24:0_18:3_18:3)	962.83024	[M+NH4]+	$C_{63}H_{110}O_6$	0.033304913	N/A	-
TG(14:0_14:0_16:0)	750.67374	[M+NH4]+	$C_{47}H_{90}O_6$	0.201840833	0.146154836	-
TG(16:2_16:3_18:3)	818.64244	[M+NH4]+	$C_{53}H_{86}O_6$	0.104609013	N/A	-
TG(16:2_18:3_18:3)	846.67374	[M+NH4]+	$C_{55}H_{90}O_6$	0.0623657152	N/A	-
TG(16:0_20:4_20:5)	900.72069	[M+NH4]+	$C_{59}H_{96}O_6$	N/A	0.247922174	-
TG(18:1_18:3_20:5)	900.72069	[M+NH4]+	$C_{59}H_{96}O_6$	N/A	0.0319026483	-
TG(18:3_18:3_18:4)	870.67374	[M+NH4]+	$C_{57}H_{90}O_6$	N/A	0.0185564075	-
TG(16:0_18:4_22:6)	898.70504	[M+NH4]+	$C_{59}H_{94}O_6$	N/A	0.449092152	-
TG(18:2_18:2_22:6)	926.73634	[M+NH4]+	$C_{61}H_{98}O_6$	N/A	0.0384540541	-
TG(18:1_18:3_22:6)	926.73634	[M+NH4]+	$C_{61}H_{98}O_6$	N/A	0.0265922764	-
TG(14:0_20:5_22:6)	896.68939	[M+NH4]+	$C_{59}H_{92}O_6$	0.116441522	N/A	-

TG(16:1_16:1_20:4)	850.70504	[M+NH4]+	$C_{55}H_{94}O_6$	2.48896137	N/A	-
TG(16:1_20:5_20:5)	896.68939	[M+NH4]+	$C_{59}H_{92}O_6$	N/A	0.0687770721	-
TG(16:0_18:1_18:3)	854.73634	[M+NH4]+	$C_{55}H_{98}O_6$	17.3307691	4.63914236	-
TG(17:0_18:2_18:2)	868.75199	[M+NH4]+	$C_{56}H_{100}O_6$	0.62715761	1.80864852	-
TG(18:1_18:1_18:2)	882.76764	[M+NH4]+	$C_{57}H_{102}O_6$	6.85390953	4.18567409	-
TG(18:1_18:3_20:0)	910.79894	[M+NH4]+	$C_{59}H_{106}O_6$	2.6934958	4.49440356	-
TG(20:0_18:2_18:2)	910.79894	[M+NH4]+	$C_{59}H_{106}O_6$	1.28340497	2.9777628	-
TG(18:1_18:3_22:0)	938.83024	[M+NH4]+	$C_{61}H_{110}O_6$	0.951312883	1.05134027	-
TG(18:1_24:0_18:3)	966.86154	[M+NH4]+	$C_{63}H_{114}O_6$	0.534088469	0.898317546	-
TG(18:2_24:0_18:2)	966.86154	[M+NH4]+	$C_{63}H_{114}O_6$	0.233375202	0.392306754	-
TG(14:0_18:2_18:3)	824.68939	[M+NH4]+	$C_{53}H_{92}O_6$	6.28380344	3.25966943	-
TG(14:0_14:0_22:5)	824.68939	[M+NH4]+	$C_{53}H_{92}O_6$	2.12490007	0.916683472	-
TG(15:0_18:2_18:3)	838.70504	[M+NH4]+	$C_{54}H_{94}O_6$	5.79283062	8.79028488	-
TG(18:1_18:4_22:6)	924.72069	[M+NH4]+	$C_{61}H_{96}O_6$	N/A	0.0444012386	-
TG(23:0_18:2_18:2)	952.84589	[M+NH4]+	$C_{62}H_{112}O_6$	0.0449613179	0.183006683	-
TG(14:0_16:0_16:0)	778.70504	[M+NH4]+	$C_{49}H_{94}O_6$	0.414858812	0.296865222	-
TG(16:0_16:0_18:0)	834.76764	[M+NH4]+	$C_{53}H_{102}O_6$	8.19806129	5.53026508	-
TG(16:3_18:3_22:6)	894.67374	[M+NH4]+	$C_{61}H_{94}O_6$	0.151527895	0.15963079	-
TG(16:0_16:0_18:3)	828.72069	[M+NH4]+	$C_{53}H_{96}O_6$	8.18510172	0.922841112	-
TG(14:0_16:0_18:3)	800.68939	[M+NH4]+	$C_{51}H_{92}O_6$	2.47190109	0.233303336	-
TG(16:0_24:0_18:2)	942.86154	[M+NH4]+	$C_{61}H_{114}O_6$	0.576230451	1.04388567	-
TG(22:0_18:1_18:1)	942.86154	[M+NH4]+	$C_{61}H_{114}O_6$	0.0378310436	0.0433144491	-
TG(16:0_18:2_22:0)	914.83024	[M+NH4]+	$C_{59}H_{110}O_6$	0.7289114	0.384454868	-
TG(18:0_18:2_20:0)	914.83024	[M+NH4]+	$C_{59}H_{110}O_6$	0.125408016	0.117106222	-
TG(16:1_18:0_20:1)	886.79894	[M+NH4]+	$C_{57}H_{106}O_6$	1.9573741	0.718371371	-
TG(18:0_18:1_18:1)	886.79894	[M+NH4]+	$C_{57}H_{106}O_6$	0.452769013	0.19366644	-
TG(16:0_16:0_20:2)	858.76764	[M+NH4]+	$C_{55}H_{102}O_6$	1.34578226	0.868893717	-
TG(16:0_18:0_18:2)	858.76764	[M+NH4]+	$C_{55}H_{102}O_6$	14.9434781	9.28659135	-
TG(20:1_20:1_18:2)	938.83024	[M+NH4]+	$C_{61}H_{110}O_6$	0.405077555	0.634379062	-
TG(16:0_17:0_18:2)	844.75199	[M+NH4]+	$C_{54}H_{100}O_6$	0.531629487	0.733673834	-
TG(18:2_18:2_22:0)	938.83024	[M+NH4]+	$C_{61}H_{110}O_6$	0.480643548	0.525414009	-
TG(18:1_22:1_18:2)	938.83024	[M+NH4]+	$C_{61}H_{110}O_6$	0.114663626	0.0450381028	-
TG(18:2_21:0_18:2)	924.81459	[M+NH4]+	$C_{60}H_{108}O_6$	0.0149316749	0.0457329969	-
TG(18:1_18:1_20:2)	910.79894	[M+NH4]+	$C_{59}H_{106}O_6$	1.66339153	1.31059174	-
TG(16:0_18:2_22:2)	910.79894	[M+NH4]+	$C_{59}H_{106}O_6$	0.129461787	0.0960107862	-
TG(16:0_16:0_18:2)	830.73634	[M+NH4]+	$C_{53}H_{98}O_6$	45.3984651	34.7035192	-
TG(16:0_16:1_18:1)	830.73634	[M+NH4]+	$C_{53}H_{98}O_6$	3.90082354	3.82671415	-

TG(15:0_16:0_18:2)	816.72069	[M+NH4]+	$C_{52}H_{96}O_6$	1.58175423	2.03807107	-
TG(14:0_16:0_18:2)	802.70504	[M+NH4]+	$C_{51}H94O_6$	8.69700188	4.58251168	-
TG(16:0_16:1_16:1)	802.70504	[M+NH4]+	$C_{51}H9_4O_6$	12.9489904	9.03397818	-
TG(16:0_18:1_20:0)	888.81459	[M+NH4]+	$C_{57}H_{108}O_6$	0.669813449	0.244100846	-
TG(16:0_20:0_18:1)	888.81459	[M+NH4]+	$C_{57}H_{108}O_6$	0.634919567	0.185577276	-
TG(16:3_18:2_22:5)	898.70504	[M+NH4]+	$C_{59}H_{94}O_6$	0.230355936	0.6431838	-
TG(18:0_18:0_18:1)	888.81459	[M+NH4]+	$C_{57}H_{108}O_6$	0.333643923	0.18361167	-
TG(18:2_18:4_18:4)	870.67374	[M+NH4]+	$C_{57}H_{90}O_6$	2.84361789	8.60079352	-
TG(18:2_18:3_18:4)	872.68939	[M+NH4]+	$C_{57}H_{92}O_6$	0.55693837	N/A	-
TG(25:0_18:2_18:2)	980.87719	[M+NH4]+	$C_{64}H_{116}O_6$	0.00726953863	0.0336419563	-
TG(20:1_24:1_18:2)	994.89284	[M+NH4]+	$C_{65}H_{118}O_6$	0.0411808994	0.101123579	-
TG(14:1_18:2_18:2)	824.68939	[M+NH4]+	$C_{53}H_{92}O_6$	1.65512064	1.15978167	-
TG(14:0_16:0_22:5)	852.72069	[M+NH4]+	$C_{55}H_{96}O_6$	1.19725145	0.25754553	-
TG(17:1_18:2_18:2)	866.73634	[M+NH4]+	$C_{56}H_{98}O_6$	0.734912969	1.77652909	-
TG(17:1_18:1_18:3)	866.73634	[M+NH4]+	$C_{56}H_{98}O_6$	0.167086144	0.215583401	-
TG(16:0_18:1_20:4)	880.75199	[M+NH4]+	$C_{57}H_{100}O_6$	0.323515536	0.348075228	-
TG(18:0_18:3_20:2)	908.78329	[M+NH4]+	$C_{59}H_{104}O_6$	0.576995417	1.29612419	-
TG(18:2_18:2_20:1)	908.78329	[M+NH4]+	$C_{59}H_{104}O_6$	5.54632524	12.4883762	-
TG(18:1_18:3_20:1)	908.78329	[M+NH4]+	$C_{59}H_{104}O_6$	9.23427506	18.3794509	-
TG(18:1_20:2_20:2)	936.81459	[M+NH4]+	$C_{61}H_{108}O_6$	0.0890540243	0.644271504	-
TG(22:1_18:2_20:2)	964.84589	[M+NH4]+	$C_{63}H_{112}O_6$	0.154990661	0.0830964787	-
TG(24:0_18:2_18:3)	964.84589	[M+NH4]+	$C_{63}H_{112}O_6$	0.340194029	0.149924286	-
TG(14:0_14:0_22:6)	822.67374	[M+NH4]+	$C_{53}H_{90}O_6$	0.696228265	0.0260938922	-
TG(14:0_18:3_18:3)	822.67374	[M+NH4]+	$C_{53}H_{90}O_6$	2.12923687	N/A	-
TG(16:0_16:3_18:3)	822.67374	[M+NH4]+	$C_{53}H_{90}O_6$	0.117791156	N/A	-
TG(15:0_18:3_18:3)	836.68939	[M+NH4]+	$C_{54}H_{92}O_6$	0.870189601	0.391408225	-
TG(16:0_18:3_18:3)	850.70504	[M+NH4]+	$C_{55}H_{94}O_6$	17.117214	N/A	-
TG(18:3_18:3_20:0)	906.76764	[M+NH4]+	$C_{59}H_{102}O_6$	0.756680132	0.0409746886	-
TG(18:3_20:1_20:2)	934.79894	[M+NH4]+	$C_{61}H_{106}O_6$	0.0624906938	0.159693089	-
TG(16:2_16:3_18:2)	820.65809	[M+NH4]+	$C_{53}H_{88}O_6$	0.214603491	0.0462195746	-
TG(20:1_18:3_18:3)	904.75199	[M+NH4]+	$C_{59}H_{100}O_6$	1.86130996	0.206881088	-
TG(18:2_18:3_20:2)	904.75199	[M+NH4]+	$C_{59}H_{100}O_6$	0.959451769	1.54805643	-
TG(14:0_16:3_20:5)	818.64244	[M+NH4]+	$C_{53}H_{86}O_6$	0.117061478	0.378210436	-
TG(18:3_18:3_20:2)	902.73634	[M+NH4]+	$C_{59}H_{98}O_6$	0.286787583	0.101419442	-
TG(18:1_18:4_18:4)	872.68939	[M+NH4]+	$C_{57}H_{92}O_6$	0.440467738	N/A	-
TG(16:0_16:0_16:0)	806.73634	[M+NH4]+	$C_{51}H_{98}O_6$	4.16076922	4.34457319	555-44-2
TG(16:0_18:0_18:1)	860.78329	[M+NH4]+	$C_{55}H_{104}O_6$	4.07016615	0.984705327	-

TG(15:0_16:0_18:1)	818.73634	[M+NH4]+	$C_{52}H_{98}O_6$	0.387968454	0.389120753	-
TG(14:0_18:1_20:2)	856.75199	[M+NH4]+	$C_{55}H_{100}O_6$	0.0884008483	0.273212459	-
TG(16:0_17:0_18:3)	842.73634	[M+NH4]+	$C_{54}H_{98}O_6$	0.191068996	0.187666111	-
TG(16:0_17:1_18:2)	842.73634	[M+NH4]+	$C_{54}H_{98}O_6$	0.521896631	1.12293498	-
TG(15:0_18:1_18:2)	842.73634	[M+NH4]+	$C_{54}H_{98}O_6$	0.309231232	0.26893503	-
TG(14:0_18:1_18:2)	828.72069	[M+NH4]+	$C_{53}H_{96}O_6$	4.2540726	1.24328511	-
TG(16:0_16:2_18:1)	828.72069	[M+NH4]+	$C_{53}H_{96}O_6$	0.286355121	0.125612608	-
TG(16:0_15:1_18:2)	814.70504	[M+NH4]+	$C_{52}H_{94}O_6$	5.08350523	3.40459375	-
TG(14:0_16:1_18:2)	800.68939	[M+NH4]+	$C_{51}H_{92}O_6$	1.62338115	N/A	-
TG(13:0_16:0_18:3)	786.67374	[M+NH4]+	$C_{50}H_{90}O_6$	0.168557602	0.276214787	-
TG(10:0_16:0_18:3)	744.62679	[M+NH4]+	$C_{47}H_{84}O_6$	0.080188427	0.016224882	-
TG(18:0_20:0_20:2)	942.86154	[M+NH4]+	$C_{61}H_{114}O_6$	0.0671403842	0.0622334143	-
TG(18:2_20:0_20:0)	942.86154	[M+NH4]+	$C_{61}H_{114}O_6$	0.0252286027	0.0216445681	-
TG(16:0_18:0_20:0)	890.83024	[M+NH4]+	$C_{57}H_{110}O_6$	0.0958813053	0.0817714197	-
TG(17:0_17:0_19:0)	876.81459	[M+NH4]+	$C_{56}H_{108}O_6$	0.996208436	0.769717044	-
TG(16:0_16:0_20:0)	862.79894	[M+NH4]+	$C_{55}H_{106}O_6$	3.55141673	2.74080102	-
TG(18:1_20:0_20:1)	942.86154	[M+NH4]+	$C_{61}H_{114}O_6$	0.0535799114	0.0268013059	-
TG(16:0_23:0_18:2)	928.84589	[M+NH4]+	$C_{60}H_{112}O_6$	0.063214054	0.114521182	-
TG(18:1_18:1_20:0)	914.83024	[M+NH4]+	$C_{59}H_{110}O_6$	0.255215781	0.141617135	-
TG(16:0_18:1_22:1)	914.83024	[M+NH4]+	$C_{59}H_{110}O_6$	0.0575062913	0.0404652695	-
TG(18:0_18:1_20:1)	914.83024	[M+NH4]+	$C_{59}H_{110}O_6$	0.320462669	0.174566926	-
TG(16:0_18:2_20:0)	886.79894	[M+NH4]+	$C_{57}H_{106}O_6$	2.44737483	1.2161135	-
TG(16:0_18:1_20:1)	886.79894	[M+NH4]+	$C_{57}H_{106}O_6$	1.52188707	0.884842073	-
TG(17:0_18:0_18:2)	872.78329	[M+NH4]+	$C_{56}H_{104}O_6$	0.141647494	0.119104365	-
TG(16:0_18:0_18:0)	862.79894	[M+NH4]+	$C_{55}H_{106}O_6$	5.98456864	4.7238971	-
TG(15:0_17:0_18:0)	834.76764	[M+NH4]+	$C_{53}H_{1}0_{2}O_{6}$	0.181478729	0.159212882	-
TG(16:0_18:0_18:3)	856.75199	[M+NH4]+	$C_{55}H_{100}O_6$	3.6551187	N/A	-
TG(16:0_16:0_18:1)	832.75199	[M+NH4]+	$C_{53}H_{100}O_6$	13.5937931	5.43721507	-
TG(17:0_18:1_18:2)	870.76764	[M+NH4]+	$C_{56}H_{102}O_6$	0.337720288	0.164765972	-
TG(16:0_18:2_20:1)	884.78329	[M+NH4]+	$C_{57}H_{104}O_6$	10.4034295	14.7751119	-
TG(14:0_16:0_18:1)	804.72069	[M+NH4]+	$C_{51}H_{96}O_6$	2.29818185	0.887399815	-
TG(16:0_16:0_16:1)	804.72069	[M+NH4]+	$C_{51}H_{96}O_6$	2.36150014	1.73375926	-
TG(14:1_16:0_16:0)	776.68939	[M+NH4]+	$C_{49}H_{92}O_6$	0.113276228	0.0711280217	-
TG(14:0_16:0_16:1)	776.68939	[M+NH4]+	$C_{49}H_{92}O_6$	1.07415272	0.963578634	-
TG(14:0_18:0_22:0)	890.83024	[M+NH4]+	$C_{57}H_{110}O_6$	0.055071638	0.0261751819	-
TG(18:0_18:3_20:1)	910.79894	[M+NH4]+	$C_{59}H_{106}O_6$	4.59111942	4.23483246	-
TG(16:0_18:1_20:3)	882.76764	[M+NH4]+	$C_{57}H_{102}O_6$	0.12211289	0.244303707	-

TG(16:0_18:2_20:2)	882.76764	[M+NH4]+	$C_{57}H_{102}O_6$	1.39730367	5.17917345	-
TG(17:1_18:1_18:2)	868.75199	[M+NH4]+	$C_{56}H_{100}O_6$	0.131029064	0.164632487	-
TG(16:1_16:2_18:1)	826.70504	[M+NH4]+	$C_{53}H_{94}O_6$	0.5250363	0.452564126	-
TG(14:0_18:2_18:2)	826.70504	[M+NH4]+	$C_{53}H_{94}O_6$	6.37352906	7.60516743	-
TG(13:0_16:0_20:4)	812.68939	[M+NH4]+	$C_{52}H_{92}O_6$	0.459702449	0.092290671	-
TG(13:0_18:1_18:3)	812.68939	[M+NH4]+	$C_{52}H_{92}O_6$	0.331493268	0.134958237	-
TG(16:0_16:1_16:3)	798.67374	[M+NH4]+	$C_{51}H_{90}O_6$	0.656777726	0.325778671	-
TG(14:0_14:0_20:4)	798.67374	[M+NH4]+	$C_{51}H_{90}O_6$	0.0815862165	0.0372488166	-
TG(12:0_18:2_18:2)	798.67374	[M+NH4]+	$C_{51}H_{90}O_6$	0.29983248	0.342831956	-
TG(18:2_20:1_22:0)	968.87719	[M+NH4]+	$C_{63}H_{116}O_6$	0.14813948	0.158123939	-
TG(20:1_20:1_20:1)	968.87719	[M+NH4]+	$C_{63}H_{116}O_6$	0.0160522779	0.0224070277	-
TG(18:1_22:0_20:2)	968.87719	[M+NH4]+	$C_{63}H_{116}O_6$	0.0626879131	0.0877280635	-
TG(23:0_18:1_18:2)	954.86154	[M+NH4]+	$C_{62}H_{114}O_6$	0.0230906098	0.0434044739	-
TG(18:2_20:0_20:1)	940.84589	[M+NH4]+	$C_{61}H_{112}O_6$	0.17878549	0.169596217	-
TG(18:1_18:1_22:1)	940.84589	[M+NH4]+	$C_{61}H_{112}O_6$	0.109492259	0.0618659552	-
TG(18:1_18:2_22:0)	940.84589	[M+NH4]+	$C_{61}H_{112}O_6$	0.324639998	0.148643202	-
TG(16:0_18:3_22:0)	912.81459	[M+NH4]+	$C_{59}H_{108}O_6$	0.275537379	0.0487674831	-
TG(20:1_18:2_18:0)	912.81459	[M+NH4]+	$C_{59}H_{108}O_6$	0.769327791	0.50486889	-
TG(16:0_18:1_20:2)	884.78329	[M+NH4]+	$C_{57}H_{104}O_6$	7.91589856	10.9937848	-
TG(16:0_18:1_18:1)	858.76764	[M+NH4]+	$C_{55}H_{102}O_6$	2.87934615	1.93072196	-

Table S7. LC-MS/MS identification of proteins in SL-EXO and RP-EXO

Description	Gene	SL-EXO	RP-EXO
Ribulose bisphosphate carboxylase large chain OS=Paris marmorata OX=221257 GN=rbcL PE=3 SV=1	rbcL	973.3927978	0.756848596
ATP synthase subunit alpha OS=Paris verticillata OX=55185 GN=atpA PE=3 SV=1	atpA	254.5815474	0
ATP synthase subunit beta OS=Paris verticillata OX=55185 GN=atpB PE=3 SV=1	atpB	149.7103363	0
Photosystem I iron-sulfur center OS=Paris verticillata OX=55185 GN=psaC PE=3 SV=1	psaC	95.08179454	0
Photosystem I P700 chlorophyll a apoprotein A1 OS=Paris polyphylla var. yunnanensis OX=221260 GN=psaA PE=3 SV=1	psaA	91.74114196	0
Photosystem I P700 chlorophyll a apoprotein A2 OS=Paris polyphylla var. yunnanensis OX=221260 GN=psaB PE=3 SV=1	psaB	77.74046136	0
ATP synthase epsilon chain, chloroplastic OS=Paris polyphylla var. yunnanensis OX=221260 GN=atpE PE=3 SV=1	atpE	51.94710701	0
ATP synthase subunit b, chloroplastic OS=Paris cronquistii	atpF	43.49589609	0

OX=220640 GN=atpF PE=3 SV=1			
Sterol glucosyltransferase (Fragment) OS=Paris polyphylla var. yunnanensis OX=221260 GN=UGT1 PE=2 SV=1	UGT1	41.18126058	0
Cytochrome b559 subunit alpha OS=Paris verticillata OX=55185 GN=psbE PE=3 SV=1	psbE	39.18651099	0
ATP-dependent Clp protease proteolytic subunit OS=Paris delavayi OX=374957 GN=clpP PE=3 SV=1	clpP	38.64218637	0
Cytochrome b559 subunit beta OS=Paris verticillata OX=55185 GN=psbF PE=3 SV=1	psbF	30.83714825	0
Cytochrome f OS=Paris polyphylla var. yunnanensis OX=221260 GN=petA PE=3 SV=1	petA	27.17588054	0
Photosystem II D2 protein OS=Paris verticillata OX=55185 GN=psbD PE=3 SV=1	psbD	19.7850412	0
Photosystem II CP47 reaction center protein OS=Paris verticillata OX=55185 GN=psbB PE=3 SV=1	psbB	18.98095107	0
Photosystem II reaction center protein H OS=Paris verticillata OX=55185 GN=psbH PE=3 SV=1	psbH	18.50881955	0
Photosystem II protein D1 OS=Paris verticillata OX=55185 GN=psbA PE=3 SV=1	psbA	16.60449364	0
Photosystem II CP43 reaction center protein OS=Paris fargesii OX=83858 GN=psbC PE=3 SV=1	psbC	15.67578736	0
Cytochrome b6-f complex subunit 4 OS=Paris verticillata OX=55185 GN=petD PE=3 SV=1	petD	10.01417934	0
Cytochrome b6 OS=Paris polyphylla var. yunnanensis OX=221260 GN=petB PE=3 SV=1	petB	7.376060412	0
Ribulose bisphosphate carboxylase large chain (Fragment) OS=Paris japonica OX=49668 GN=rbcL PE=3 SV=1	rbcL	7.362423938	0
Ribulose bisphosphate carboxylase large chain (Fragment) OS=Paris axialis OX=221258 GN=rbcL PE=3 SV=1	rbcL	7.178367998	0
Cytochrome P450 CYP94D109 OS=Paris polyphylla OX=49666 GN=CYP94D109 PE=1 SV=1	CYP94 D109	4.990463381	0
NAD(P)H-quinone oxidoreductase subunit 5, chloroplastic OS=Paris stigmatosa OX=2735601 GN=ndhF PE=3 SV=1	ndhF	4.782318768	0
Small ribosomal subunit protein uS4c OS=Paris polyphylla var. yunnanensis OX=221260 GN=rps4 PE=3 SV=1	rps4	4.554412206	0
Ribulose bisphosphate carboxylase large chain OS=Paris dulongensis OX=374960 GN=rbcL PE=3 SV=1	rbcL	4.064033901	0
Acetyl-coenzyme A carboxylase carboxyl transferase subunit beta, chloroplastic OS=Paris polyphylla var. yunnanensis OX=221260 GN=accD PE=3 SV=1	accD	3.723081535	0

Small ribosomal subunit protein uS19c OS=Paris polyphylla var. yunnanensis OX=221260 GN=rps19 PE=3 SV=1	rps19	3.44737034	0
ATP synthase subunit a, chloroplastic OS=Paris verticillata OX=55185 GN=atpI PE=3 SV=1	atpI	3.294703409	0
Ribosomal protein OS=Paris verticillata OX=55185 GN=rpl36 PE=3 SV=1	rpl36	3.045138917	0
Maturase K OS=Paris tetraphylla OX=49670 GN=matK PE=3 SV=1	matK	2.778097376	0
Photosystem I assembly protein Ycf4 OS=Paris polyphylla var. yunnanensis OX=221260 GN=ycf4 PE=3 SV=1	ycf4	2.759307724	0
Protein TIC 214 OS=Paris polyphylla var. yunnanensis OX=221260 GN=ycf1 PE=3 SV=1	ycf1	2.744342443	0
Ribulose bisphosphate carboxylase large chain (Fragment) OS=Paris verticillata OX=55185 GN=rbcL PE=3 SV=1	rbcL	2.566987255	0
Large ribosomal subunit protein uL14c OS=Paris verticillata OX=55185 GN=rpl14 PE=3 SV=1	rpl14	2.286633338	0
Small ribosomal subunit protein bS16c OS=Paris stigmatosa OX=2735601 GN=rps16 PE=3 SV=1	rps16	2.262447435	0
Small ribosomal subunit protein uS8c OS=Paris verticillata OX=55185 GN=rps8 PE=3 SV=1	rps8	1.931566452	0
50S ribosomal protein L23 OS=Paris verticillata OX=55185 GN=rpl23 PE=3 SV=1	rpl23	1.746481498	0
Small ribosomal subunit protein uS2c OS=Paris polyphylla var. yunnanensis OX=221260 GN=rps2 PE=3 SV=1	rps2	1.730462895	0
Ribulose bisphosphate carboxylase large chain OS=Paris bashanensis OX=374954 GN=rbcL PE=3 SV=1	rbcL	1.67399671	0
NADH-quinone oxidoreductase subunit D OS=Paris verticillata OX=55185 GN=ndhH PE=3 SV=1	ndhH	1.647323346	0
Maturase K (Fragment) OS=Paris polyphylla OX=49666 GN=matK PE=3 SV=1	matK	1.638402515	0
Terpene cyclase/mutase family member OS=Paris polyphylla var. yunnanensis OX=221260 GN=CAS PE=2 SV=1	CAS	1.588312983	0
Methyltransferase OS=Paris polyphylla OX=49666 GN=SMT1 PE=2 SV=1	SMT1	1.560489066	0
Cytochrome P450 CYP94D108 OS=Paris polyphylla OX=49666 GN=CYP94D108 PE=1 SV=1	CYP94 D108	1.534220825	0
Cytochrome b6-f complex subunit 5 OS=Paris verticillata OX=55185 GN=petG PE=3 SV=1	petG	1.521661981	0
Large ribosomal subunit protein bL20c OS=Paris polyphylla var. yunnanensis OX=221260 GN=rpl20 PE=3 SV=1	rpl20	1.490038001	0
DNA-directed RNA polymerase subunit beta OS=Paris fargesii	rpoB	1.347037328	0

OX=83858 GN=rpoB PE=3 SV=1			
sterol 3beta-glucosyltransferase OS=Paris polyphylla var. chinensis OX=221254 GN=UGT45 PE=2 SV=1	UGT45	1.300498493	1.243151404
Cholesterol 16,22-dihydroxylase CYP90G4 OS=Paris polyphylla OX=49666 GN=CYP90G4 PE=1 SV=1	CYP90G4	1.275678785	0
Cytochrome P450 CYP72A616 OS=Paris polyphylla OX=49666 GN=CYP72A616 PE=1 SV=1	CYP72A616	1.222072776	0
Large ribosomal subunit protein uL16c OS=Paris polyphylla var. yunnanensis OX=221260 GN=rpl16 PE=3 SV=1	rpl16	1.212803539	0
Small ribosomal subunit protein uS3c OS=Paris polyphylla var. yunnanensis OX=221260 GN=rps3 PE=3 SV=1	rps3	1.153704049	0
NADH-quinone oxidoreductase subunit C OS=Paris verticillata OX=55185 GN=ndhJ PE=3 SV=1	ndhJ	1.142644163	0
Large ribosomal subunit protein bL33c OS=Paris verticillata OX=55185 GN=rpl33 PE=3 SV=1	rpl33	1.040650143	0
Small ribosomal subunit protein uS12c OS=Paris verticillata OX=55185 GN=rps12 PE=3 SV=1	rps12	1	0
Small ribosomal subunit protein uS19c OS=Paris verticillata OX=55185 GN=rps19 PE=3 SV=1	rps19	0.956635526	0
DNA-directed RNA polymerase subunit beta' OS=Paris polyphylla var. yunnanensis OX=221260 GN=rpoC1 PE=3 SV=1	rpoC1	0.940527796	0
Small ribosomal subunit protein uS15c OS=Paris fargesii OX=83858 GN=rps15 PE=3 SV=1	rps15	0.897009375	0
NAD(P)H-quinone oxidoreductase subunit I, chloroplastic OS=Paris polyphylla var. yunnanensis OX=221260 GN=ndhI PE=3 SV=1	ndhI	0.874500685	0
MADS-box transcription factor (Fragment) OS=Paris verticillata OX=55185 GN=PverAGL6b PE=2 SV=1	PverAGL6b	0.870165858	0
Ribulose bisphosphate carboxylase large chain (Fragment) OS=Paris thibetica OX=83859 GN=rbcL PE=3 SV=1	rbcL	0.861528614	0
Ribulose bisphosphate carboxylase large chain (Fragment) OS=Paris nitida OX=2497474 GN=rbcL PE=3 SV=1	rbcL	0.851902867	0
GA20ox1 (Fragment) OS=Paris polyphylla var. yunnanensis OX=221260 GN=GA20ox1 PE=2 SV=1	GA20ox1	0.849682788	0
Ribulose bisphosphate carboxylase large chain (Fragment) OS=Paris quadrifolia OX=231488 GN=rbcL PE=3 SV=1	rbcL	0.836240773	0
Ribulose bisphosphate carboxylase large chain OS=Paris rugosa OX=374966 GN=rbcL PE=3 SV=1	rbcL	0.803240182	0
NADH-quinone oxidoreductase subunit H OS=Paris verticillata OX=55185 GN=ndhA PE=3 SV=1	ndhA	0.785412295	0
Large ribosomal subunit protein uL2c OS=Paris polyphylla var.	rpl2	0.771532746	0

yunnanensis OX=221260 GN=rpl2 PE=3 SV=1			
Protein TIC 214 OS=Paris mairei OX=374964 GN=ycf1 PE=3 SV=1	ycf1	0.765606592	0
Photosystem I assembly protein Ycf3 OS=Paris polyphylla var. yunnanensis OX=221260 GN=ycf3 PE=3 SV=1	ycf3	0.758309499	0
Large ribosomal subunit protein uL22c OS=Paris verticillata OX=55185 GN=rpl22 PE=3 SV=1	rpl22	0.737761609	0
NADH-quinone oxidoreductase subunit B OS=Paris verticillata OX=55185 GN=ndhK PE=3 SV=1	ndhK	0.736583508	0
Glycosyltransferase OS=Paris polyphylla var. yunnanensis OX=221260 GN=UGT6 PE=2 SV=1	UGT6	0.680124616	0
DNA-directed RNA polymerase subunit alpha OS=Paris polyphylla var. yunnanensis OX=221260 GN=rpoA PE=3 SV=1	rpoA	0.60163346	0
ATP-dependent Clp protease proteolytic subunit OS=Paris stigmatosa OX=2735601 GN=clpP PE=3 SV=1	clpP	0.538149718	0
Small ribosomal subunit protein uS13m OS=Paris quadrifolia OX=231488 GN=rps13 PE=3 SV=1	rps13	0.534135749	0
Squalene synthase OS=Paris polyphylla var. yunnanensis OX=221260 PE=2 SV=1	--	0.520916553	0
Small ribosomal subunit protein uS7c OS=Paris polyphylla var. yunnanensis OX=221260 GN=rps7 PE=3 SV=1	rps7	0.44145715	0
Chalcone-flavonone isomerase family protein OS=Paris polyphylla var. yunnanensis OX=221260 GN=CHI PE=2 SV=1	CHI	0.424672052	0
Large ribosomal subunit protein uL16c OS=Paris tetraphylla OX=49670 GN=rpl16 PE=3 SV=1	rpl16	0.413712637	0
Protein TIC 214 OS=Paris quadrifolia OX=231488 GN=ycf1 PE=3 SV=1	ycf1	0.410079485	0
MADS-box transcription factor (Fragment) OS=Paris japonica OX=49668 GN=KjapAG1-2 PE=2 SV=1	KjapAG1-2	0.404953856	0
DNA-directed RNA polymerase subunit beta" OS=Paris polyphylla var. yunnanensis OX=221260 GN=rpoC2 PE=3 SV=1	rpoC2	0.395700014	0
Ribulose bisphosphate carboxylase large chain (Fragment) OS=Paris polyphylla OX=49666 GN=rbcL PE=3 SV=2	rbcL	0.372692211	0
Cholesterol 22-hydroxylase CYP90B27 OS=Paris polyphylla OX=49666 GN=CYP90B27 PE=1 SV=1	CYP90B27	0.372079664	0
Protein Ycf2 OS=Paris japonica OX=49668 GN=ycf2 PE=3 SV=1	ycf2	0.315768237	0
Ribulose bisphosphate carboxylase large chain (Fragment) OS=Paris polyphylla OX=49666 GN=rbcL PE=3 SV=1	rbcL	0.291955047	0
Small ribosomal subunit protein uS11c OS=Paris polyphylla var. yunnanensis OX=221260 GN=rps11 PE=3 SV=1	rps11	0.289763327	0
gibberellin 2beta-dioxygenase OS=Paris polyphylla var. yunnanensis	GA2ox	0.270255795	0

OX=221260 GN=GA2ox1 PE=2 SV=1	1		
Small ribosomal subunit protein uS11c OS=Paris verticillata OX=55185 GN=rps11 PE=3 SV=1	rps11	0.248198414	0
Small ribosomal subunit protein bS18c OS=Paris verticillata OX=55185 GN=rps18 PE=3 SV=1	rps18	0.246119317	0
Small ribosomal subunit protein uS14c OS=Paris verticillata OX=55185 GN=rps14 PE=3 SV=1	rps14	0.227878204	0
DNA-directed RNA polymerase subunit beta OS=Paris polyphylla var. yunnanensis OX=221260 GN=rpoB PE=3 SV=1	rpoB	0.192168953	0
Ribulose bisphosphate carboxylase large chain (Fragment) OS=Paris verticillata OX=55185 GN=rbcL PE=3 SV=1	rbcL	0.166148648	0
Glycosyltransferase OS=Paris polyphylla var. chinensis OX=221254 PE=2 SV=1	--	0.159122177	0
NADH-quinone oxidoreductase subunit N OS=Paris verticillata OX=55185 GN=ndhB PE=3 SV=1	ndhB	0.14871049	0
Cytochrome c biogenesis protein CcsA OS=Paris polyphylla var. yunnanensis OX=221260 GN=ccsA PE=3 SV=1	ccsA	0.132682164	0
Protein Ycf2 OS=Paris polyphylla var. yunnanensis OX=221260 GN=ycf2 PE=3 SV=1	ycf2	0.1202878	0
UDP-glycosyltransferase (Fragment) OS=Paris polyphylla var. yunnanensis OX=221260 GN=UGT7 PE=2 SV=1	UGT7	0.120248908	0
Terpene cyclase/mutase family member OS=Paris polyphylla var. yunnanensis OX=221260 GN=CAS PE=2 SV=1	CAS	0.118214375	0
Ribulose bisphosphate carboxylase large chain (Fragment) OS=Paris mairei OX=374964 GN=rbcL PE=3 SV=1	rbcL	0.099391504	0
Cytochrome c oxidase subunit 3 OS=Paris quadrifolia OX=231488 GN=cox3 PE=3 SV=1	cox3	0.096565359	0
Large ribosomal subunit protein bL32c OS=Paris verticillata OX=55185 GN=rpl32 PE=3 SV=1	rpl32	0.091138317	0
DNA-directed RNA polymerase (Fragment) OS=Paris polyphylla var. chinensis OX=221254 GN=rpoB PE=3 SV=1	rpoB	0.082499453	0
DNA-directed RNA polymerase subunit alpha OS=Paris japonica OX=49668 GN=rpoA PE=3 SV=1	rpoA	0.082487299	0
Hypothetical chloroplast RF1 OS=Paris verticillata OX=55185 GN=ycf1 PE=4 SV=1	ycf1	0.079915896	0
Protein TIC 214 OS=Paris dulongensis OX=374960 GN=ycf1 PE=3 SV=1	ycf1	0.049258623	0
Protein TIC 214 OS=Paris luquanensis OX=221256 GN=ycf1 PE=3 SV=1	ycf1	0.038043332	0
ATP-dependent Clp protease proteolytic subunit OS=Paris verticillata	clpP	0.034463575	0

OX=55185 GN=clpP PE=3 SV=1			
NAD(P)H-quinone oxidoreductase chain 4 OS=Paris verticillata OX=55185 GN=ndhD PE=3 SV=1	ndhD	0.026571111	0
ATP synthase CF0 B chain subunit I OS=Paris verticillata OX=55185 GN=atpF PE=3 SV=1	atpF	0.025684619	0

[1]The table provides protein descriptions, gene names, and MS peak areas for each identified protein in both PDNVs samples.

Table S8. Evaluation of Key Topological Parameters in the Protein-Protein Interaction Network (The top 12 Degrees).

Rank	Name	Betweenness	Closeness	Degree
1	GAPDH	0.12102038	0.67924528	61
2	AKT1	0.08739265	0.66666667	60
3	TNF	0.06738802	0.64285714	58
4	SRC	0.13379595	0.64285714	55
5	EGFR	0.07855393	0.63157895	51
6	ESR1	0.04915069	0.58695652	44
7	PTGS2	0.02958487	0.58378378	41
8	GSK3B	0.02071819	0.56842105	39
9	MMP9	0.01764047	0.57446809	37
10	IL2	0.01967365	0.55384615	33
11	MAPK1	0.01372684	0.55102041	33
12	PTPRC	0.00986004	0.54545455	30

Table S9. Specific primer sequences used for RT-qPCR analysis.

Gene Name	Forward Primer (5' → 3')	Reverse Primer (5' → 3')
MITF	TACGTTGGCCTGTCTCCAAG	CAGGGCACATTTGGGATACT
TYR	CGGCCCAAATTGTACAGAGAAGC	CTGCCAGGAGAAGAAGGATTG
TRP-1	AAGCGCCACCCTCCACTAT	TGAACTAAGGACGGGGTACAC
β-ACTIN	AGAAGATCTGGCACCACACC	TACGACCAGAGGCATACAGG

Table S10. Docking Analysis of the Active Ligands with Core Receptor Targets

Active Compounds	Docking Score (kcal/mol)
4-p-Coumaroylquinic acid	-8.2
BOA-6-O-glucoside	-7.6
chrysophanol-8-o-beta-d-glucoside	-8.7
Cyanidin 3-O-(6-O-p-coumaroyl)glucoside	-10.9
Kaempferol-3-O-glucoside-7-O-rhamnoside	-8.9
Uridine Monophosphate	-7.3
Cogformer	-9.4
